# Incidence of resistance to ALS and ACCase inhibitors in *Echinochloa* species and soil microbial composition in Northern Italy

**DOI:** 10.1038/s41598-024-59856-0

**Published:** 2024-05-08

**Authors:** Carlo Maria Cusaro, Enrica Capelli, Anna Maria Picco, Maura Brusoni

**Affiliations:** Department of Earth and Environmental Sciences, University of Pavia, 27100 Pavia, Italy

**Keywords:** Next-generation sequencing, Archaea, Bacteria, Microbial communities, Fungi, Soil microbiology, Plant sciences

## Abstract

The increasing amount of weeds surviving herbicide represents a very serious problem for crop management. The interaction between microbial community of soil and herbicide resistance, along with the potential evolutive consequences, are still poorly known and need to be investigated to better understand the impact on agricultural management. In our study, we analyzed the microbial composition of soils in 32 farms, located in the Northern Italy rice-growing area (Lombardy) with the aim to evaluate the relationship between the microbial composition and the incidence of resistance to acetolactate synthase (ALS) and acetyl-CoA carboxylase (ACCase) inhibiting herbicides in *Echinochloa* species. We observed that the coverage of weeds survived herbicide treatment was higher than 60% in paddy fields with a low microbial biodiversity and less than 5% in those with a high microbial biodiversity. Fungal communities showed a greater reduction in richness than *Bacteria*. In soils with a reduced microbial diversity, a significant increase of some bacterial and fungal orders (i.e. *Lactobacillales*,* Malasseziales* and *Diaporthales*) was observed. Interestingly, we identified two different microbial profiles linked to the two conditions: high incidence of herbicide resistance (H-HeR) and low incidence of herbicide resistance (L-HeR). Overall, the results we obtained allow us to make hypotheses on the greater or lesser probability of herbicide resistance occurrence based on the composition of the soil microbiome and especially on the degree of biodiversity of the microbial communities.

## Introduction

Weed management is one of the most critical aspects in agriculture. Each year considerable worldwide yield losses are estimated, due to the negative impact of weeds on crop production^1^. The annual worldwide cost of crop losses caused by weed infestation is estimated around 32 billions of USD^2^. To date, chemical control has represented the most efficient tool for managing weeds. However, herbicide resistance (HeR) represents an up-and-coming phytosanitary threat to worldwide agricultural systems and is an example of the adaptive evolution of weeds in response to human selective pressures^3^. The increasing amount of weeds surviving herbicide represents a very serious problem particularly for those territories traditionally suited to rice cultivation such as Po Valley in Northern Italy. In this area, a survey on the spread of herbicide resistance has been underway for a long time through monitoring studies and laboratory testing with the aim of highlighting the causes favoring the phenomenon. It was seen that resistance can persist for several years due to the seed stock in the soil^4^.

In addition, artificial selection of agronomic traits in rice (*Oryza sativa* L.), which are useful to humans, has unintentionally promoted the evolution of crop-like weed biotypes. As a result, the weeds can evade chemical control and eradication from fields, allowing them to spread throughout the agroecosystem. In fact, weeds has evolved an adaptive phenomenon (*Vavilovian mimicry*) which allowed them to resemble domesticated crops at specific stages in their life history. This phenotypic adaptation results in a morphological similarity of weeds to crops, making difficult for farmers to distinguish them^5,6^.

A lot of studies have demonstrated that herbicide resistance can be ascribed either to a DNA missense mutation in genes expressing specific proteins targeted by herbicides (target site resistance – TSR), or to herbicides detoxification processes (non-target site resistance – NTSR)^7^. Moreover, the increasing repeated field applications of an increasingly narrow range of herbicides, as a consequence of the withdrawal of many plant protection products (PPPs) from the EU market due to strict regulation (Reg EC/1107/2009)^8^, and the lack of herbicides with new modes of action (MoAs), determine a continuous rise and spread of the resistance^9–12^. Furthermore, other factors may influence herbicide resistance, such as epigenetic mechanisms (i.e. miRNAs) regulating the expression of genes encoding for enzymes involved in xenobiotic detoxification^13^. Environmental traits may also be related to the occurrence of herbicide resistance, such as the rise of temperature^14^ which could affect chemical control efficacy. New information are emerging on the effects of herbicides on soil microbial communities. However, the interaction between microbial community and herbicide application, along with the potential consequences, are still poorly known and need to be investigated to better understand the possible relapses on agricultural management. Paddy soil microbiota was discovered to be critical to the wellness and fitness of rice, but also of herbicide resistant weeds. It has been discovered that different biomass allocation and root traits resulted for rice in the presence of herbicide-resistant *Echinochloa crus-galli* (L.) P. Beauv (barnyardgrass), since this weed recruited a distinct microbial consortium in the rhizosphere soil through its exudates^15^. It is known that root systems releases various plant metabolites through root exudation. Weeds and crops exudates may be selective for specific soil microbial groups, stimulating their development in the soil. This may favor symbiotic and associative interactions between plants and microorganisms, which could be important to plants in the uptake of nutrients and water, but also against phytopathogens^16^.

As a matter of facts, even if herbicides are thought to be specific to plants, it has been recently reported that they can affect other types of organisms by targeting evolutionarily conserved pathways. For instance, the growth of *Stenotrophomonas maltophilia* (*Xanthomonadales* order), an aerobic non-fermentative gram-negative bacillus, sometimes present in rice fields, resulted negatively affected by herbicides^17^. Moreover, an unsuspected impact on diversity and composition of microbial communities has been revealed by glyphosate, a common broad-spectrum herbicide, since its targets enzyme (shikimate pathway, 5-enolpyruvylshikimate-3-phosphate synthase – EPSPS) is present in many prokaryotes and fungi^18^.

Hence, herbicide exposure can change microbial communities because microbes differ in their intrinsic susceptibility. As a consequence, differences in sensitivity can lead to changes in the abundance of certain microbes under herbicide exposure. Moreover, many microbes can metabolize herbicides and some can use them as sources of nutrients^19^. Therefore, herbicide residues may increase the abundance of herbicide-metabolizing microbes with a cascade effect on the community. Thus, changes due to herbicide exposures can impact microbial composition inducing changes on community functionality. Further, the physical-chemical characteristics of the soils and climatic conditions influence soil microbiome, which results consequently variable^19,20^.

The aim of the present study was to evaluate the relationship between the soil microbial (*Bacteria*, *Archaea* and *Fungi*) composition of paddy fields in the Po Valley, an area traditionally vocated to rice production, and the incidence of herbicide-resistant weeds. *E. crus-galli* and *Echinochloa oryzicola* (Vasinger) Vasinger (late-watergrass) are the most problematic weeds infesting Italian rice fields due to their high ability to develop resistance to the most commonly used herbicides^21^. Among the most widespread and noxious weeds infesting rise cultivation, the species of the genus *Echinochloa* P. Beauv., tribe *Paniceae* R. Br. subfamily *Panicoideae* A. Br., family *Gramineae* Juss (= *Poaceae* Barnh.), are the worst due to their wide ecological success and ability to mimic the crops^5,6,22^.

Soil microorganisms have an important role in the metabolization of macronutrients and in the production of bioactive molecules that play a pivotal role at the rhizosphere level and in the interaction between plants. A recent study reported that the relationships between microbial communities and plants are controlled by the type of plants and the microorganisms involved. Plant microbiota could be beneficial or pathogenic to the plant^23^. Weeds can mitigate soil microbiota that colonize the rhizosphere and acquire nutrient sources unavailable to the crop, thereby creating soil conditions that favor weeds increasing their competitive ability^24–26^. Moreover, the composition of soil microbiota is impacted by herbicide treatments^27–29^.

Systemic herbicides released through weed roots may select microorganisms that mediate detrimental processes such as nutrient immobilization or serve as opportunistic pathogens. Kremer^16^ highlighted the implications of herbicide resistance on soil biology and ecology, pointing out that herbicides may compound effects of weeds on soil microorganisms. Some studies have reported that repeated herbicide exposure during weed control favors increased herbicide tolerance in *Bacteria*^30^. Herbicide tolerance can be achieved via genetic changes in the herbicide target gene^31^ or non-target genes linked with generalized stress tolerance^32,33^. However, the relationship among weeds, soil properties and soil microbial communities has not been fully explored. Soil organisms are an integral component of ecosystems, but their activities receive little recognition in agricultural management strategies. Soil organisms have the potential to enhance ecosystem service delivery and soil biodiversity promotes multiple ecosystem functions simultaneously (i.e. ecosystem multifunctionality).

The data obtained could be useful to implement more effective weed control strategies and to apply more sustainable methods of intervention in farming practices.

## Results

### Identification of farms affected by herbicide resistance phenomena

For the present research we have considered 32 rice farms, located in the provinces of Pavia and Milano (Lombardy region, Italy) and managed according to the Directive 2009/128 EC^34^ that establishes a framework to achieve a sustainable use of pesticides promoting the use of integrated pest management (IPM) that aims to keep the use of pesticides and other forms of intervention only to levels that are economically and ecologically justified and which reduce or minimize risk to human health and the environment. The principles on which it is based concern seed selection, fertilization, the use of plant protection products and strategies to reduce herbicide resistance. IPM is mandatory since 1 January 2014.

**Figure 1:**
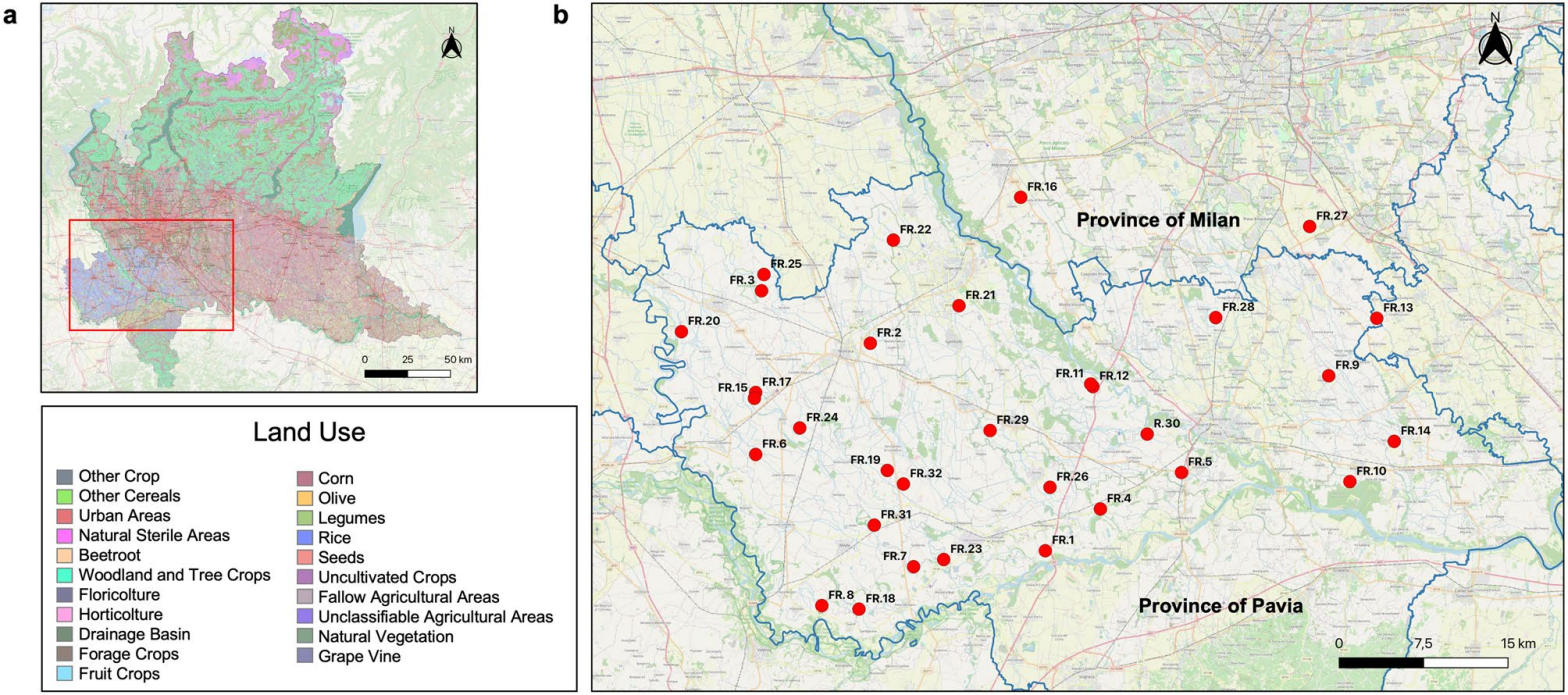
(**a**) map of the land use in the Lombardy region (SIARL 2019; DUSAF 7.0, 2023). The rice cropping area, in the provinces of Pavia and Milano is bordered in red. (**b**) distribution of the rice farms (FR) where *E. crus-galli* and *E. oryzicola* survived herbicide were detected. The maps were generated through Q-GIS software, version 3.32 Lima (http://qgis.osgeo.org).

In the Figure 1 is displayed the rice cropping area of the Lombardy region (Figure 1a) within the provinces of Pavia and Milan where *Echinochloa* spp. specimens survived after the application of acetyl-CoA carboxylase (ACCase – HRAC group: A) and acetolactate synthase (ALS – HRAC group: B) inhibitors were identified (Figure 1b). The distribution of farms where herbicide resistant *Echinochloa* were observed are indicated with red marks. The map obtained following our survey indicates that this phoenomenon is widely spread-out within the Lombardy region, including areas where the presence of *Echinochloa* HeR was not reported (GIRE)^4^.

### *Echinochloa* spp. herbicide resistance (HeR) incidence

Two species of *Echinochloa* were identified in the rice fields investigated for this study: *E. crus-galli* was recorded in 15 out of 32 of the surveyed farms, while *E. oryzicola* in 21 out of 32 of the farms. Both species were found only in 4 farms (Table 1).

**Table 1:** Values of abundance (% coverage of the plant-area projection on the paddy area) of the *E. crus-galli* and *E. oryzicola* specimens survived herbicide administration in the surveyed rice farms.

Farm	*E. crus-galli*	*E. oryzicola*	Total HeR incidence
FR.1	1.00	4.00	5.00
FR.2	5.00	0.00	5.00
FR.3	0.00	37.50	37.50
FR.4	17.50	0.00	17.50
FR.5	5.00	32.50	37.50
FR.6	37.50	0.00	37.50
FR.7	62.50	0.00	62.50
FR.8	0.00	87.50	87.50
FR.9	0.00	62.50	62.50
FR.10	1.00	5.00	5.00
FR.11	0.00	17.50	17.50
FR.12	0.00	17.50	17.50
FR.13	2.00	2.00	5.00
FR.14	0.00	37.50	37.50
FR.15	0.00	5.00	5.00
FR.16	0.00	5.00	5.00
FR.17	62.50	0.00	62.50
FR.18	0.00	62.50	62.50
FR.19	62.50	0.00	62.50
FR.20	0.00	17.50	17.50
FR.21	37.50	0.00	37.50
FR.22	0.00	17.50	17.50
FR.23	0.00	17.50	17.50
FR.24	17.50	0.00	17.50
FR.25	0.00	17.50	17.50
FR.26	0.00	17.50	17.50
FR.27	5.00	0.00	5.00
FR.28	0.00	5.00	5.00
FR.29	17.50	0.00	17.50
FR.30	17.50	0.00	17.50
FR.31	0.00	5.00	5.00
FR.32	0.00	5.00	5.00

Total HeR incidence: midpoint of cover range (according to Braun-Blanquet scale) of the sum of *E. crus-galli* and *E. oryzicola* % coverage.

The incidence of specimens surviving herbicide treatment (HeR incidence) was assessed as percent coverage. Based on the values in Table 1, it is possible to differentiate farms with a low incidence (coverage less than 5%), farms with a medium incidence (coverage between 5 and 60%) and farms with a high incidence (coverage higher than 60%).

Values of low HeR incidence were reported in 9 rice farms (FR.1, FR.2, FR.10, FR.13, FR.15, FR.16, FR.27, FR.28, FR.31 and FR.32). Values of medium HeR incidence were recorded in 17 rice farms (FR.3, FR.4, FR.5, FR.6, FR.11, FR.12, FR.14, FR.20, FR.21, FR.22, FR.23, FR.24, FR.25, FR.26, FR.29 and FR.30). Values of high HeR incidence were recorded in 6 rice farm (FR.7, FR.8, FR.9, FR.17, FR.18 and FR.19).

### Microbial biodiversity analysis

The sequencing of amplicons on the Illumina MiSeq Platform produced a total of 1,989,664 raw reads for *Bacteria* and *Archaea* (on average 62,177.00 per sample) and a total of 3,000,941 raw reads for *Fungi* (on average 93,779.41 per sample).

After quality filtering, 362,811.00 sequences remained (on average 11,337.84 per sample) for *Bacteria* and *Archaea* and 699,877.00 sequences (on average 21,871.16 per sample) for *Fungi*.

The Greengenes 16S and the UNITE ITS reference datasets were used for determining operational taxonomic units (OTUs) at the 99% level. A total of 51 phyla, 141 orders, 238 genera and 127 species was identified for *Bacteria* and *Archaea*. A total of 4 phyla, 16 orders, 16 genera and 15 species was identified for *Fungi*. Of these, the amount of unassigned and unidentified OTUs was 81% and 68% respectively. In Supplementary Table 1 are listed the identified bacterial, archaeal and fungal orders. Only taxa with a prevalence at phylum level equal or higher than 5% were considered for the analysis.

Microbial biodiversity (α-diversity) was evaluated at the taxonomic rank of orders by computing Margalef (richness), Shannon (diversity), Simpson (dominance) and Pielou (evenness) indexes (Table 2).

**Table 2:** Margalef richness, Shannon diversity, Simpson dominance and Pielou evenness indexes at the taxonomic rank of orders for *Bacteria* plus *Archaea* and for *Fungi.*

ID	*Bacteria* plus *Archaea*				*Fungi*			
	Margalef	Shannon	Simpson	Pielou	Margalef	Shannon	Simpson	Pielou
FR.1	14.73	3.47	0.06	0.81	4.01	0.87	0.51	0.34
FR.2	15.13	3.51	0.05	0.81	4.67	0.87	0.65	0.32
FR.3	15.34	3.40	0.06	0.78	3.67	1.18	0.45	0.47
FR.4	13.72	3.36	0.05	0.79	4.01	1.24	0.36	0.48
FR.5	13.12	3.23	0.06	0.77	3.00	0.94	0.50	0.41
FR.6	15.13	3.53	0.04	0.82	4.67	0.82	0.71	0.30
FR.7	11.70	2.90	0.09	0.71	1.00	0.57	0.71	0.41
FR.8	10.90	3.40	0.05	0.85	1.34	0.36	0.85	0.23
FR.9	18.36	3.59	0.05	0.79	2.00	0.97	0.51	0.50
FR.10	16.34	3.65	0.04	0.83	2.67	1.59	0.26	0.72
FR.11	16.55	2.80	0.15	0.63	3.00	1.70	0.24	0.74
FR.12	13.52	3.06	0.07	0.73	4.01	1.68	0.25	0.66
FR.13	14.33	3.39	0.05	0.79	2.34	0.67	0.71	0.32
FR.14	11.50	3.31	0.05	0.82	3.34	1.87	0.19	0.78
FR.15	17.15	3.52	0.05	0.79	3.34	1.59	0.31	0.66
FR.16	9.28	3.12	0.06	0.81	3.67	1.10	0.42	0.44
FR.17	3.83	2.57	0.10	0.86	1.34	0.63	0.65	0.39
FR.18	19.57	3.68	0.04	0.80	2.00	0.72	0.65	0.37
FR.19	4.44	1.75	0.29	0.56	1.34	0.63	0.67	0.39
FR.20	13.92	3.38	0.06	0.80	4.67	0.85	0.67	0.31
FR.21	14.93	3.49	0.05	0.81	2.00	1.55	0.27	0.80
FR.22	17.76	3.56	0.04	0.79	4.34	1.19	0.49	0.45
FR.23	14.53	3.43	0.06	0.80	4.67	1.73	0.28	0.64
FR.24	15.13	3.57	0.04	0.82	4.01	1.25	0.45	0.49
FR.25	14.12	3.51	0.05	0.82	4.34	1.59	0.28	0.60
FR.26	18.56	3.52	0.05	0.78	3.67	1.44	0.34	0.58
FR.27	13.92	3.26	0.07	0.77	3.67	1.30	0.45	0.52
FR.28	15.94	3.27	0.07	0.75	4.67	1.70	0.26	0.63
FR.29	16.34	3.63	0.04	0.82	3.34	1.00	0.54	0.42
FR.30	14.33	3.42	0.05	0.80	4.67	1.90	0.22	0.70
FR.31	14.53	3.47	0.05	0.81	4.67	1.28	0.44	0.47
FR.32	17.15	3.41	0.06	0.77	3.00	1.73	0.23	0.75

ID: identification code.

In the soil samples analyzed, the bacterial and archaeal communities showed higher average values of richness, diversity and evenness and lower average values of dominance compared to the fungal ones.

As concerns bacterial and archaeal communities, the highest values of the Margalef index were observed in samples FR.9, FR.15, FR.18, FR.22, FR.26 and FR.32 while the lowest were observed in FR.17 and FR.19. Shannon index showed high values (> 3) except for the FR.7, FR.11, FR.17 and FR.19 soil communities. Simpson index values were low in all samples except for FR.11 and FR.19, that represent poor communities characterized by the dominance of one order (*Actinomycetales* with 32% in FR.11; *Actinomycetales* and *Rickettsiales* both with 35% in FR.19). The Pielou index, which ranges from 0 to 1, showed high values except in FR.11 and FR.19, which were characterized by communities with dominance of a single or few orders. The high evenness recorded in all samples is an indicator that communities draw on resources equally.

As concerns fungal communities, samples FR.2, FR.6, FR.20, FR.23, FR.28, FR.30 and FR.31 were characterized by the highest values of the Margalef richness index, while the soil communities sampled in farms FR.7, FR.8, FR.17 and FR.19 were the poorest. Shannon index showed in general low values except in FR.14 and FR.30 soil communities. Simpson index showed low values in samples FR.10, FR.11, FR.12, FR.14, FR.28, FR.30 and FR.32, communities characterized by high values of Pielou evenness index. The highest value of Simpson index and the lowest value of Pielou index were registered in FR.8 soil community, which is characterized by the dominance of the *Malasseziales* order (with 92%).

### Herbicide resistance and microbial composition of soils

**Figure 2:**
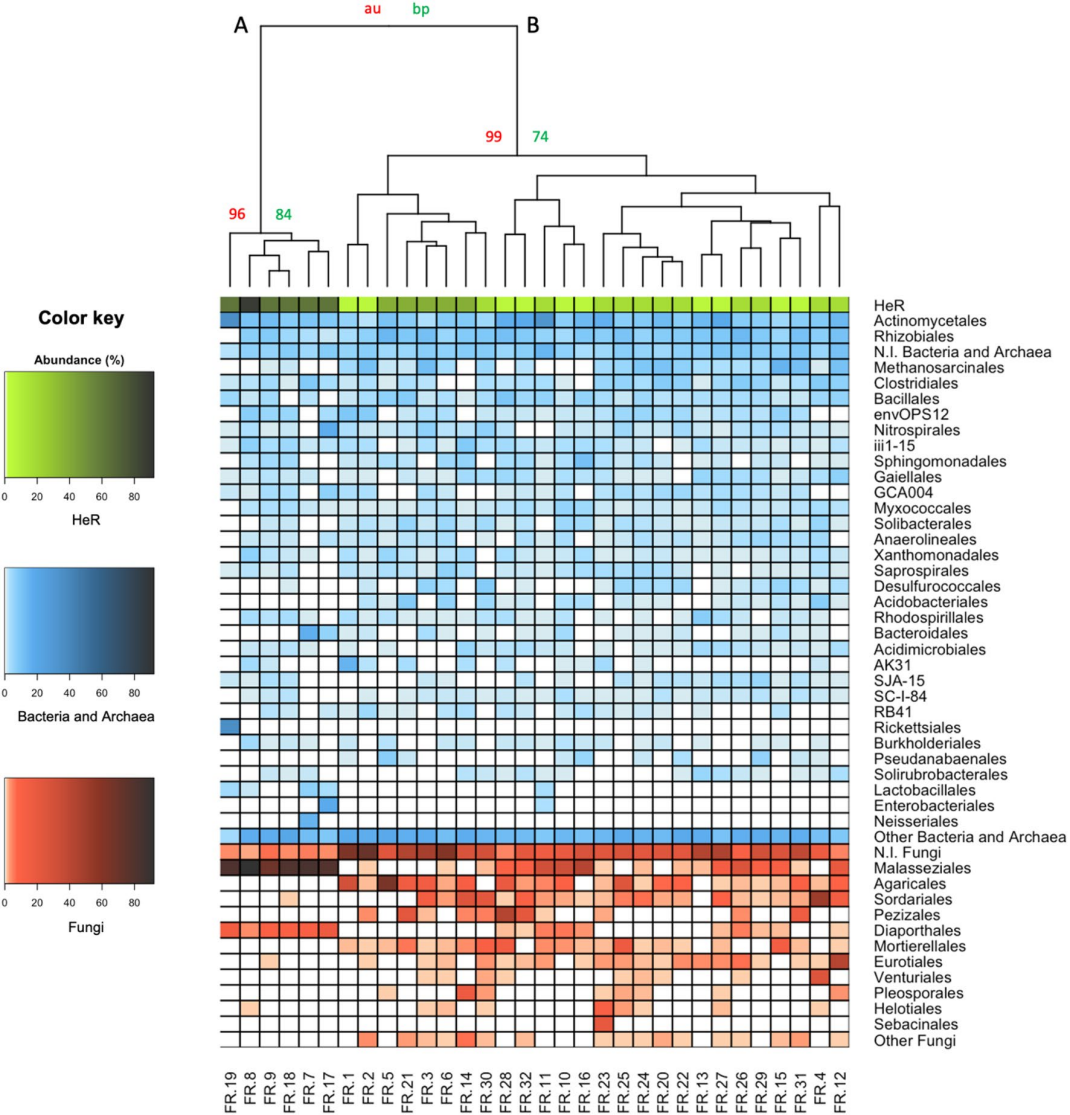
Heatmap and bootstrap-based hierarchical clustering based on “Bray-Curtis” distance and “ward.D2” algorithm. The incidence of resistant *Echinochloa* spp. (HeR-green), the prevalence of bacterial and archaeal (blue) and of fungal (red) orders were considered. FR.: rice farm. N.I.: taxa not identified. Other: all orders with a prevalence value < 5%. au: approximately unbiased. bp: bootstrap probability.

We searched for a relationship between the microbial composition of soils and the incidence of herbicide resistance. Figure 2 shows the heatmap and dendrogram obtained from the bootstrap-based hierarchical clustering of the paddy soils considering both the incidence of resistant *Echinochloa* spp. (HeR) and the prevalence of microbial orders.

Two main clusters were identified. Cluster A grouped the soils in which a higher incidence of resistant *Echinochloa* spp. and a lower microbial diversity were recorded. Otherwise, cluster B included the soils in which a lower incidence of resistant *Echinochloa* spp. and a higher microbial diversity were observed. It was also noted that some bacterial, archaeal and fungal orders possessed higher prevalence in cluster A than in cluster B, and vice versa. For example, *Rickettsiales*,* Enterobacteriales*,* Lactobacillales*, *Neisseriales*, *Malasseziales* and *Diaporthales* were more abundant in soils of cluster A, while *Rhizobiales*,* Methanosarcinales*, *Pseudanabaenales*, *Agaricales*,* Sordariales*,* Pezizales* and *Mortirellales* were more represented in cluster B. These results suggested a possible relationship between certain bacterial and fungal orders and the presence of resistant *Echinochloa* spp.

Envfit analysis revealed that *Actinomycetales*, *Rhizobiales*, *Methanosarcinales*, *Gaiellales*, *Solibacterales*, *Desulfurococcales*, *Pseudanabaenales*, *Solirubrobacterales*, *Lactobacillales*, *Malasseziales*, *Agaricales*, *Sordariales*, *Diaporthales* and *Venturiales* were the bacterial, archaeal and fungal orders which significantly contribute to the classification of farms within the two clusters A and B (Supplementary Table 2).

**Figure 3:**
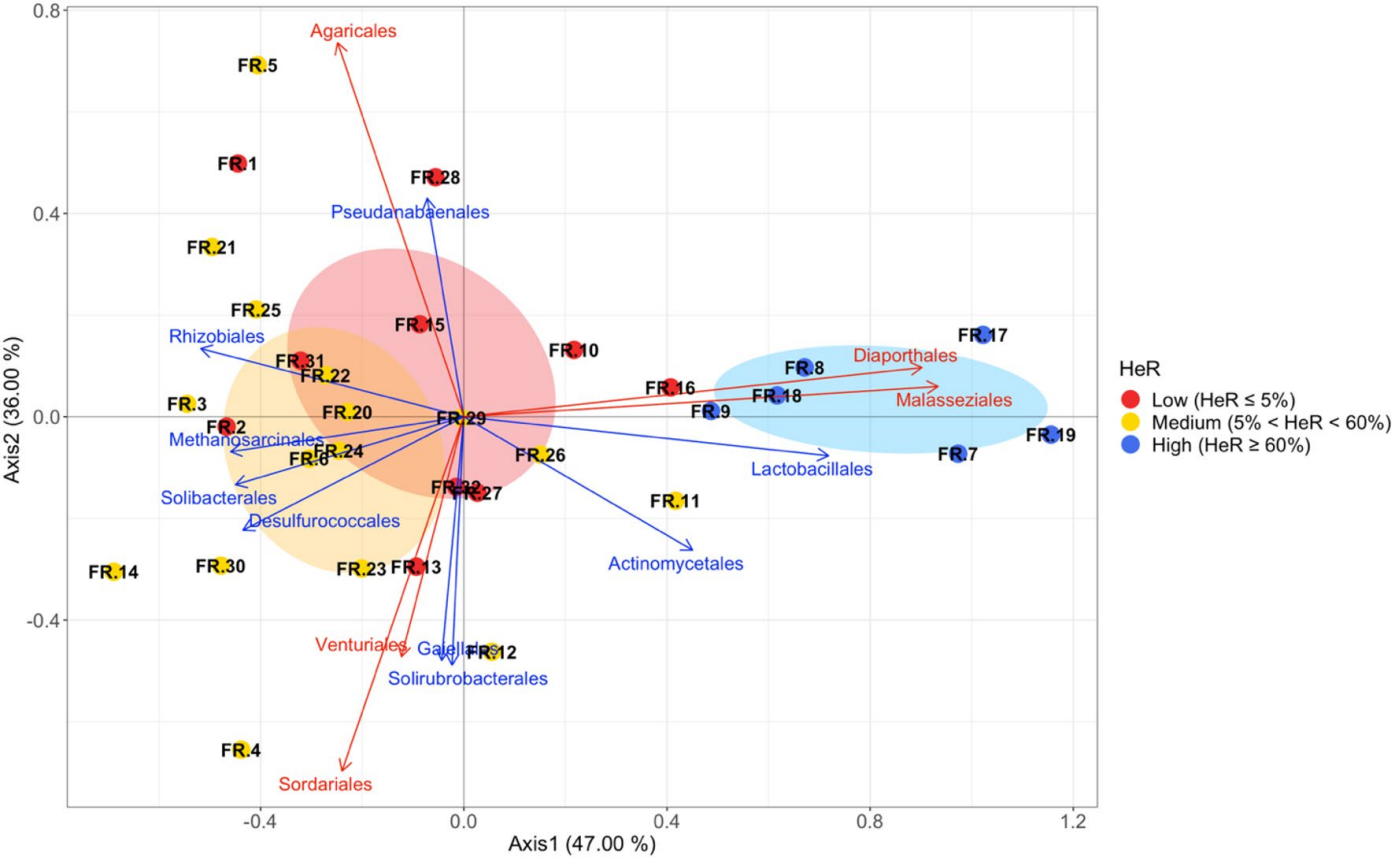
Principal Coordinates Analysis (PCoA) of the 32 soil samples collected. Values on brackets represent the percent variation explained by coordinate 1 and coordinate 2 respectively. For microbial communities, the taxonomic rank of order was considered. Blue arrows: bacterial and archaeal orders. Red arrows: fungal orders. Red ellipse: L-HeR soils group. Yellow ellipse: M-HeR soils group. Blue ellipse: H-HeR soils group. Ellipses assumed a multivariate normal distribution. Confidence level: 0.99.

From the Principal Coordinates Analysis (PCoA), three distinct groups of soils were identified in relation to the incidence of herbicide resistance and the hosted microbial communities (Figure 3): soils with low incidence (L-HeR – red ellipse), soils with medium incidence (M-HeR – yellow ellipse) and soils with high incidence (H-HeR – blue ellipse). The analysis revealed that in the H-HeR group of soils, *Actinomycetales*, *Lactobacillales*, *Diaporthales* and *Malasseziales* are the prevalent orders. Otherwise, in the L-HeR and M-HeR groups, *Rhizobiales*,* Methanosarcinales*,* Gaiellales*,* Solibacterales*,* Desulfurococcales*,* Pseudanabaenales*,* Solirubrobacterales*, *Agaricales*,* Sordariales* and *Venturiales* are the mainly present orders.

The contribution of microbial communities composition in the variations of the incidence of resistant *E. crus-galli* and *E. oryzicola* was tested by redundancy analysis (RDA).

**Figure 4:**
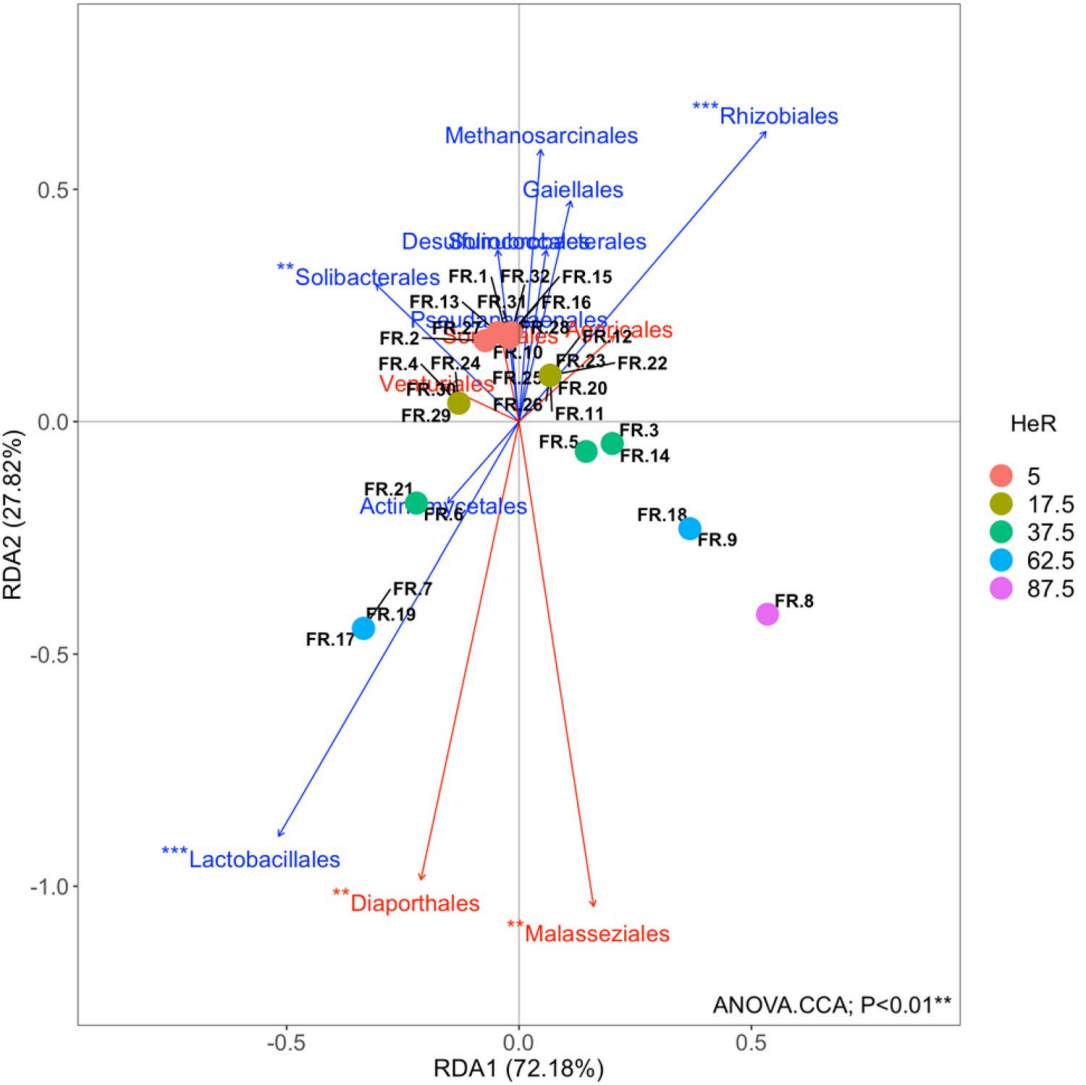
Redundancy analysis (RDA) ordination diagram of the first two axes for the incidence of herbicide resistance (HeR). Values on brackets represent the percent variation explained by axis 1 and axis 2 respectively. The constrained sets of bacterial, archaeal and fungal orders analyzed are indicated as vectors. *Lactobacillales*,* Rhizobiales*,* Solibacterales*,* Malasseziales* and *Diaporthales* resulted in a significant relation with HeR incidence in the RDA (anova.cca, *P* < 0.01**).

A significant contribution was recorded by bacterial and archaeal communities (anova.cca, *P* < 0.01), mainly explained by *Lactobacillales*,* Rhizobiales* and *Solibacterales* orders and by fungal communities (anova.cca, *P* < 0.01), mainly explained by *Malasseziales* and *Diaporthales* orders (Figure 4 – Supplementary Table 3). In particular, *Lactobacillales*,* Malasseziales* and *Diaporthales* resulted in a positive relation with the high incidence of HeR, otherwise *Rhizobiales* and *Solibacterales* resulted in a positive relation with the low incidence of HeR.

The values of abundance of microbial taxa resulting the most discriminant between the two extreme scenarios H-HeR and L-HeR soils (*Actinomycetales*,* Rhizobiales*,* Methanosarcinales*,* Gaiellales*,* Solibacterales*,* Desulfurococcales*,* Pseudanabaenales*,* Solirubrobacterales*, *Lactobacillales*, *Malasseziales*,* Agaricales*,* Sordariales*,* Diaporthales* and *Venturiales*) were compared (Supplementary Tables 4 and 5).

**Figure 5:**
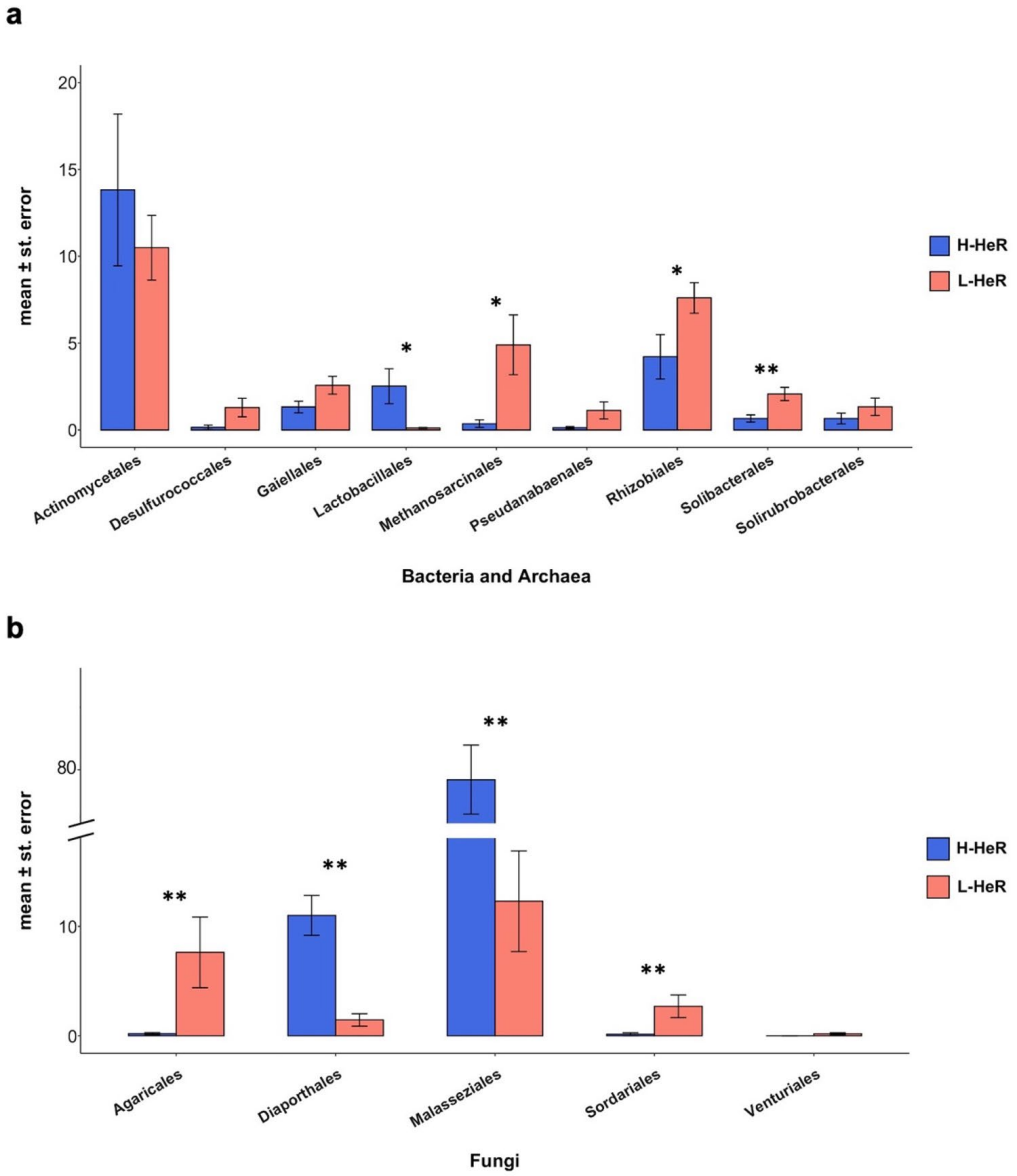
Microbial orders (mean ± standard error) with different abundance in H-HeR and L-HeR paddies. (**a**) *Bacteria* plus *Archaea*. (**b**) *Fungi*. Mann–Whitney test (two tailed); *P < 0.05; **P < 0.01.

Three bacterial taxa (*Lactobacillales*,* Rhizobiales* and *Solibacterales*), one archaeal taxon (*Methanosarcinales*) and four fungal taxa (*Agaricales*,* Diaporthales*,* Malasseziales* and *Sordariales*) recorded significantly (two tailed Mann-Whitney test, *P* < 0.05), different abundance values (Figure 5 and Supplementary Table 6).

Comparing paddies with a low resistance incidence (L-HeR) with those recording a high resistance incidence (H-HeR), it was observed that the microbial composition is different both from a quantitative and qualitative point of view (Supplementary Tables 4 and 5).

In fact, considering Bacteria and Archaea, a major abundance of *Rhizobiales*, *Methanosarcinales*, *Bacillales*, *Desulfurococcales*, *Acidobacteriales*, *Solibacterales*, *Saprospirales*, *Pseudanabaenales* (on average, 7.60%, 4.90%, 3.51%, 1.28%, 1.49%, 2.07%, 1.55% and 1.11% respectively) was generally observed in L-HeR soils, otherwise high levels of *Rickettsiales*, *Bacteroidales*, *Nitrospirales*, *Enterobacteriales*, *Neisseriales* and *Lactobacillales* (on average, 5.98%, 3.94%, 3.85%, 2.88%, 2.54% and 2.52% respectively) were found in H-HeR soils (Supplementary Table 1).

At genus/species level, *Porphyromonas* (*Bacteroidales*), was detected in most H-HeR soils, with the highest prevalence in FR.7 (7.87%), where *P. endodontalis* was the most represented species (5.91%). *Corynebacterium* and *Micrococcus* (*Actinomycetales*) were never detected in L-HeR soils, while recorded an abundance equal to 9.08% and 10.95% in FR.19 (H-HeR group) soil respectively. *Propionibacterium* recorded high abundance in 83% of the H-HeR soils (16.95%), particularly in FR.17 and FR.19, where is mainly represented by *Propionibacterium acnes* which assumed abundance values equal to 6.14% and 8.20% respectively. *Bacillus fumarioli* (*Bacillales*) was the prevalent species, especially in FR.16 and FR.28 soils (L-HeR). *Prevotella* genus (*Bacteroidales*) was prevalent in H-HeR paddies, with the highest abundance value recorded in FR.7 (6.92%), in which *P. tannerae*,* P. pallens* and *P. intermedia* were recognized. *Aeropyrum* genus (*Desulfurococcales*) was more abundant in L-HeR soils, with the highest abundance recorded in FR.15 and FR.31 soils. *Streptococcus infantis* (*Lactobacillales*) was highly recorded in FR.7, FR.17 and FR.19 soils (H-HeR) while its presence is negligible in L-HeR soils, where it was identified only in FR.28 with an abundance equal to 0.03%. *Methanosarcina* genus (*Methanosarcinales*) was abundant in almost all L-HeR soils, with the highest prevalence assumed in FR.15 (12.62%). *Neisseria* (*Neisseriales*) showed a high abundance in H-HeR soils, especially in FR.7, where *N. subflava* recorded 5.60%. *Bradyrhizobium* and *Methylosinus* were the most abundant genera among the *Rhizobiales*, recording the highest abundances mainly in L-HeR soils. *Kaistobacter* (*Sphingomonadales*) recorded the highest abundance (11.47%) in FR.16 soil (L-HeR) (Supplementary Table 4).

Considering *Fungi*, the comparison between the two groups showed that *Malasseziales* and *Diaporthales* were the most prevalent in H-HeR paddies (on average, 80.04% and 11.00% respectively), otherwise a high prevalence of *Pezizales*, *Agaricales*,* Mortierellales* and *Sordariales* (on average 8.52%, 7.62%, 3.83% and 2.70% respectively) was recorded in L-HeR soils (Supplementary Table 1).

At genus/species level, *Malassezia*, represented by the species *M. restricta* and *M. globosa*, recorded the highest prevalence values in H-HeR soils (Supplementary Table 5).

According to European and Mediterranean Plant Protection Organization (EPPO) Global Database^35^, providing basic information for species of interest to agriculture, forestry and plant protection and detailed information for pest species that are of regulatory interest (EPPO and EU listed pests, as well as pests regulated in other parts of the world), out of the micro-organisms identified and listed in Supplementary Tables 4 and 5, only *Rhodococcus fascians*, order *Actinomycetales*, is of phytosanitary relevance but EU regulated as no quarantine pest.

### Physical–chemical properties of soils

The physical-chemical parameters of the different paddy soils are listed in the Supplementary Table 7. All the soils analyzed possessed a sandy/silty texture. Most of the soils resulted acidic and slightly acidic and with a low or medium nitrogen content (N). Considering C/N ratio, 8 soils (FR.6, FR.8, FR.16, FR.19, FR.20, FR.21, FR.23 and FR.25) resulted with a low value, 13 soils (FR.3, FR.4, FR.7, FR.10, FR.11, FR.12, FR.13, FR.14, FR.18, FR.22, FR.24, FR.28 and FR.29) with a balanced value, 10 soils (FR.2, FR.5, FR.9, FR.15, FR.17, FR.26, FR.27, FR.30, FR.31 and FR.32) with a high value. Limestone (CaCO_3_) resulted absent in all soils except FR.1 and FR.14 which resulted however not very calcareous. Content of organic matter resulted low in 13 soils (FR.1, FR.4, FR.6, FR.8, FR.16, FR.19, FR.20, FR.21, FR.26, FR.28, FR.29, FR.30 and FR.31), medium in 8 soils (FR.2, FR.5, FR.9, FR.15, FR.17, FR.23, FR.24 and FR.32), high in 11 soils (FR.3, FR.7, FR.10, FR.11, FR.12, FR.13, FR.14, FR.18, FR.22, FR.25, FR.27). The majority of soils (25/32) resulted with a high or very high content of assimilable phosphorus (P) and the remaining soils with a low or very low content. Only one of the soils studied (FR.7) showed physical and chemical characters different from all others, with the highest content of silt and clay, calcium (Ca), magnesium (Mg) and with a high nitrogen (N) content.

### Physical–chemical properties of soils and microbial composition

We also wanted to verify whether soil microbial biodiversity and composition could be related to soil physical-chemical properties. From the redundancy analysis (RDA) none of the analyzed physical-chemical characteristics recorded significant effect (anova.cca, *P* > 0.05) on the composition of bacterial, archaeal and fungal communities (Supplementary Table 8).

### Herbicide resistance and physical–chemical properties of soils

**Figure 6:**
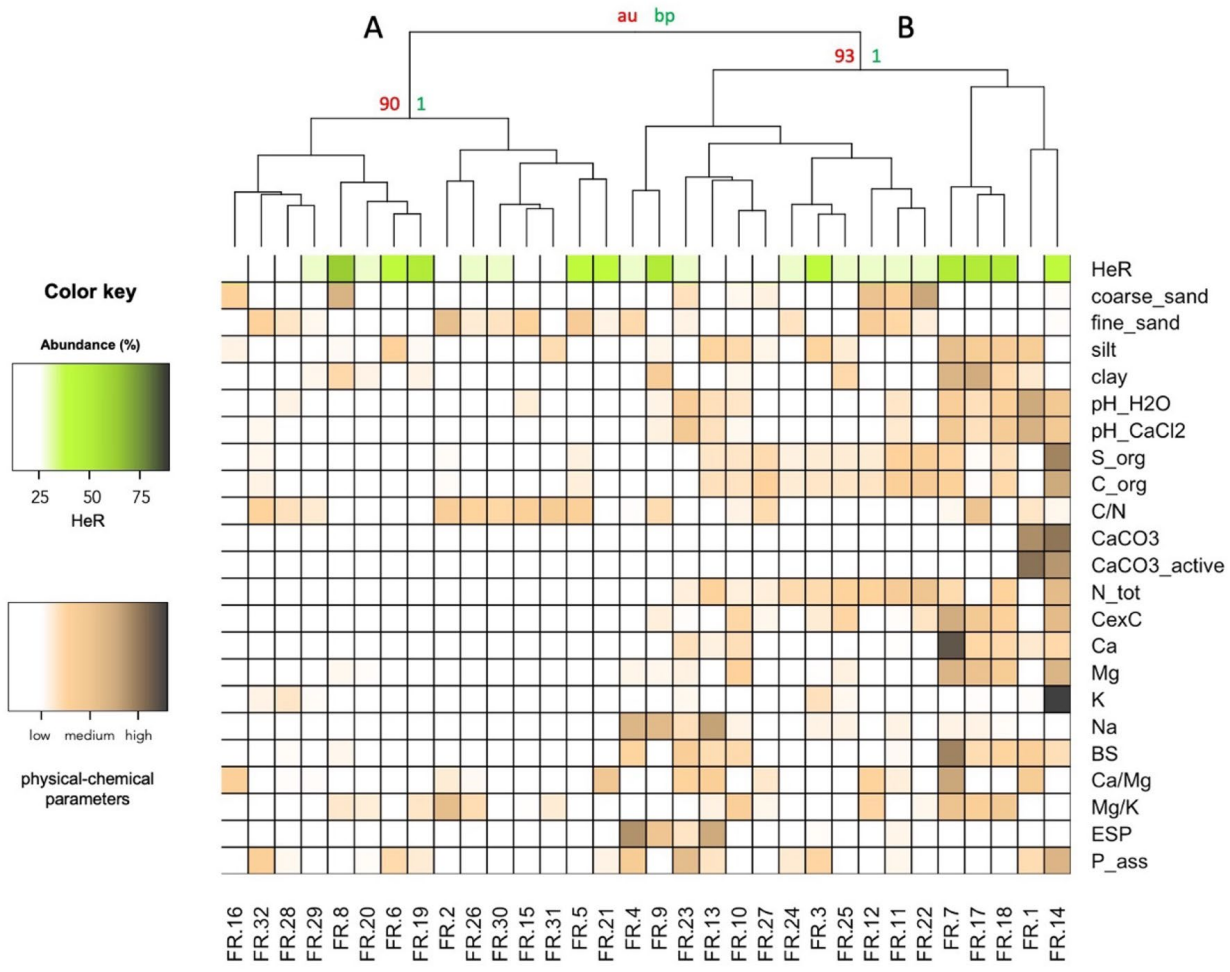
Heatmap and bootstrap-based hierarchical clustering based on “Canberra” distance and “ward.D2” algorithm. The incidence of resistant *Echinochloa* spp. (HeR) (green) and of the physical–chemical properties of soils (brown) were considered. au: approximately unbiased. bp: bootstrap probability.

The relationship between the incidence of resistant *Echinochloa* spp. (HeR) and the physical-chemical properties of soils was analyzed. The obtained heatmap and bootstrap-based dendrogram are reported in Figure 6.

Two main clusters were identified: cluster A characterized by soils with thick texture (coarse and fine sand), low content of organic matter, organic carbon and macronutrients, cluster B including soils with fine texture (silt and clay), high content of organic matter, organic carbon and macronutrients. From the heatmap and hierarchical clustering results, it can be noticed that soils with high incidence of herbicide resistance (intense green color) are equally distributed in both clusters.

Envfit analysis revealed that soil texture, pH, organic matter content, CaCO_3_, N, Ca, Mg, K and Na content, cationic exchange capacity, base saturation, were the physical-chemical edaphic properties which significantly contribute to the classification of farms within the two clusters A and B (Supplementary Table 9).

The contribution of edaphic physical-chemical characteristics in the variations of the incidence of resistant *Echinochloa* spp. (HeR) was tested by redundancy analysis (RDA) that revealed no significant association (anova.cca, *P* > 0.05) (Supplementary Table 10).

## Discussion

For the present study, we analyzed the microbial composition in the soils of 32 farms located in the Lombardy rice-growing area, all managed according to Directive 2009/128 EC^34^, in which specimens of *E. crus-galli* and *E. oryzicola* resistant to herbicide treatments were detected.

We examined the incidence of resistant *Echinochloa* spp. specimens in the paddies of the considered rice farms, highlighting that herbicide resistance phenomenon is particularly spread within the rice cropping territory of the Lombardy region, also in areas where it resulted underestimated. Various incidence values of herbicide resistance were observed, however this variability did not result related to the different types of herbicide used and their MoAs. Weed infestation is likely to be generated from a highly persistent soil seed bank, as a result of seed dormancy and resistance to conventional weed control strategies, as reported by previous studies^36^.

Microbial communities-diversity analysis highlighted a variability in the richness, diversity, dominance and evenness indexes assessed in the considered soils for *Bacteria*, *Archaea* and *Fungi*. Also in this case no relation with herbicide treatment and their modes of action was revealed. In fact, it has been noticed that bacterial and fungal communities characterized by very differing indexes were detected also in rice farms where the same type of herbicide has been applied. For example, FR.17 and FR.18 paddies, treated both with clethodim (ACCase inhibitor), recorded respectively the lowest and the highest value in bacterial richness. Moreover, the highest richness and diversity of fungal communities were recorded in FR.2, FR.20, FR.23, FR.28, FR.30 and FR.31 paddies, which were treated with different herbicides.

Concerning soil physical-chemical properties, our findings revealed that, according to the Italian standards (see Methods), the majority of the analyzed soils possess a prevalent sandy/silty texture, an acid or slightly acid pH and a medium or low content of macronutrient. Most of the considered soils possess a low or medium content of organic matter, a balanced mineralization degree (C/N), a medium nitrogen content, a low or medium cationic exchange capacity, a very low content of Ca, Mg, K and Na and a medium or high content of assimilable P. All soils are extremely poor in CaCO_3_. In general, all soils possess a quite similar chemistry, hence the variation in microbial composition cannot be related to physical-chemical parameters. These results were expected because all the rice fields are located in the Po Valley, a homogeneous area from a climatic and geological point of view and furthermore all the considered farms perform the same type of agronomic management.

The abundance of weeds that survived herbicides was greater in paddy fields where a low microbial biodiversity was recorded. Fungal communities appeared to be more involved in the phenomenon, showing a greater reduction in richness than *Bacteria* and *Archaea*. Together with a general reduction of microbial diversity, we observed a significant increase of some orders, particularly *Malasseziales*, *Diaporthales* and *Lactobacillales*.

A differential microbial composition was observed in H-HeR soils and L-HeR soils. H-HeR paddies showed the highest abundance of *Actinomycetales*, *Lactobacillales*, *Diaporthales* and *Malasseziales.* Otherwise, *Rhizobiales*, *Methanosarcinales*, *Gaiellales*, *Solibacterales*, *Desulfurococcales*, *Pseudanabaenales*, *Solirubrobacterales*, *Agaricales*, *Sordariales* and *Venturiales* resulted higher in L-HeR paddies.

From RDA analysis, *Lactobacillales*, *Malasseziales* and *Diaporthales* resulted in a positive relation with the high incidence of HeR, otherwise *Rhizobiales* and *Solibacterales* resulted in a positive relation with the low incidence of HeR.

*Lactobacillales* are an order of gram-positive, acid-tolerant bacteria, usually found in decomposing plants and milk products. They produce lactic acid as the major metabolic end-product of carbohydrate fermentation, giving them the common name lactic acid bacteria (LAB). Proteinaceous bacteriocins are produced by several LAB strains and provide an additional hurdle for spoilage and pathogenic microorganisms. Bacteriocin metabolites are toxic to microbes^37^. In addition, organic acids are the prominent secondary metabolites that exhibit antifungal activity and preservative effects in fermented food and silage^38^. Recent studies reported LAB strains as promising candidates for sustainable agriculture, since they promote soil health and fertility^39^. Considering the results of our research, it would be useful to conduct further analysis to deepen the relation between high abundance of *Lactobacillales* in soils and high incidence of herbicide resistant weeds, that forces the farmer to perform more frequent treatments, in contrast to agricultural sustainability.

*Malasseziales* is a heterogeneous group of species, and several species comprise multiple genotypes associated with mammalian hosts, but using culture-independent techniques they were also retrieved from much wider-spread habitats, including various terrestrial and marine ecosystems and even deep-sea sediments. Furthermore, *Malassezia* DNA was detected from soil nematodes in Central European forests, and it has been hypothesized that nematodes may serve as a vector for *Malassezia* species. *M. restricta* and *M. globosa* were associated with the nematode genus *Malenchus* spp., whereas another nematode, *Tylolaimophorus typicus* hosted only *M. restricta*^40,41^.

In our study we identified at species level *M. restricta* and *M. globosa* with the highest prevalence in H-HeR soils. Moreover, H-HeR paddies were characterized by the absence of some genera and species that were found in L-HeR ones (i.e. *Lycoperdon pratense* (*Agaricales*) recovered in 80% of soils and *Bolbitius coprophilus* (*Agaricales*) recovered in 40% of soils). *Mortierella* (*Mortierellales*) observed in both groups, but showed the lowest values in H-HeR soils.

The order *Diaporthales* includes plant pathogens, plant endophytes, saprobes, human-animal pathogens, and soil inhabitants, some of which have extensive host ranges and geographical distributions^42^. As endophytes, they live in medicinal plants and are used for studies that investigate antimicrobial activities, e.g., *Diaporthe* spp., which were isolated from the hosts *Copaifera langsdorffii* and *C. pubiflora*^43^. Antibacterial activity has been demonstrated using extracts of two unidentified *Diaporthe* spp. and *D. miriciae*^44^.

The high prevalence of *Diaporthales* and *Malasseziales* we observed in association with the high incidence of herbicide-resistant weeds and with the reduced microbial biodiversity, suggests a possible influence of environmental conditions (i.e. herbicide treatments) causing the selective growth of stress tolerant microorganisms, with a high capacity to regulate gene expression in response to changes in temperature, saltiness, nutrient content, acidity fluctuations as reported for different *Bacteria* and *Fungi* (i.e. *Malasseziales*)^45–48^.

*Rhizobiales* are well-known beneficial partners in plant-microbe interactions. They commonly exert beneficial functions for their hosts by providing various nutrients, phytohormons as well as precursors for essential plant metabolites^49–51^. The order contains many genera of nitrogen-fixing, methanotrophic, legume-nodulating and microsymbiotic bacteria^52,53^.

The order *Solibacterales* produces enzymes to metabolize complex organic compounds or macromolecules available in its environment for metabolism and participates in nitrate and nitrite reduction. Some studies have shown that *Solibacterales* thrives in soils rich in phosphorus. In fact, *Solibacterales* is a proficient P miner and a bioindicator of the organic P nutrient‐use economy, as its relative abundance is strongly related to phosphatase activity^54^. This order might play a role in making phosphorous bioavailable in ecosystems with differing nutrient levels^55,56^.

One of the soils studied (FR.7) showed a microbial composition clearly differing from all the others, with increased abundance of *Porphyromonas* and *Prevotella* (*Bacteroidales*) and *Neisseria* (*Neisseriales*). Strains of the family *Porphyromonadaceae* were isolated from temperate deciduous forest soil exposed to pesticide^57^. *Prevotella* is a genus of bacteria that commonly associate with humans in various body sites and is spread in various environmental niches^58^. *Neisseria* is a large genus of human and animal commensal bacteria within the normal microbiota of the human and animal nasopharynx. It comprises opportunistic pathogens and only two strains are recognized as pathogenic (*N. gonorreae* and *N. meningitidis*). *Neisseria* was recovered also in environment from soil and water. The ability of *Neisseria* to degrade organic pollutants has been confirmed in different contexts. *Neisseria* have been also found in sites closely associated with humans^59^. Recent reports evidenced the presence of *Neisseria* in soil treated with herbicides^60,61^. This fact could explain the higher abundance of *Neisseriales* in a soil characterized by a high incidence of HeR.

The different microbial profiles that characterize H-HeR and L-HeR soils can be explained as a possible selection of certain microbes due to the capacity of weeds to release in soil, through root exudation, metabolites able to drive the composition of microbial communities in accordance with previously published studies^16^.

Plants can modify the rhizosphere microbiota to affect the growth of conspecific and interspecific plants, displaying a variety of effects on each other. Plant–plant interactions can shape soil bacterial and fungal communities via root exudates. The root exudates from resistant *E. crus-galli* are responsible for the assembly and establishment of the root microbial structure. In particular, resistant *E. crus galli* is able to assemble more *Proteobacteria* and *Ascomycota* to enhance plant stress tolerance^15^.

Another possible explanation is the selection, due to repeated weeding, of stress-tolerant microorganisms with a high capacity to regulate gene expression, as reported by some researchers. It is known that herbicide treatments induce modifications in the transcription activity (RNA metabolism) and in the carbohydrate metabolism of the bacteria present in the soil. For example, an increase in the production of the F0F1 ATP synthase subunit, along with increased expression of a cytochrome c-553-related transcript, was observed in bacteria exposed to glyphosate^29,33,48,62^.

In our condition, the differential microbial profiles observed cannot be related to the different type of management, because all farms are conducted with the same strategy, as mentioned above. *Oryza sativa* L. cover cannot be a factor influencing the variability of soil microbial composition since it is homogeneous in all the rice fields investigated as found in intensive crops.

The relationship that we highlighted between the incidence of herbicide resistance in *E. crus-galli* and *E. oryzicola* and the composition and biodiversity of the microbial communities of paddy soils represents an innovative result. Recent studies have demonstrated the ability of herbicide-resistant specimens of *E. crus-galli* to influence the microbial diversity and composition of paddy fields by means of root exudates^15^. Others have highlighted how seed microbiome assembly is associated with the herbicide resistance evolution in barnyardgrass^63,64^. To date, no study had yet shown differences in soil microbial communities related with higher or lower incidence of herbicide resistant *E. crus-galli* and *E. oryzicola* specimens. Considering that plant-associated soil microbial communities have an important role not only in soil nutrient cycling, soil enzyme activity, plant growth promotion, disease suppression but also in abiotic stress tolerance, the assessment of soil microbial composition can represent an important indicator for predicting the development of herbicide resistance phenomena.

## Concluding remarks

Understanding complex interactions of weeds with soil microorganisms and improving the biodiversity of soil microbial communities could be strategic in developing more effective approaches to herbicide-resistant weed management and plant protection, with the final aim of optimizing precision weed management (PWM) technologies.

The relation between HeR incidence variability and different microbial communities allows us to make hypotheses on the greater or lesser probability of herbicide resistance occurrence, based on the composition and α-diversity of the soil microbiome. The type of weed affects the composition of soil microbiome, favoring the growth of some microbial groups to the detriment of others. The repeated treatments with the same type of herbicide can favor both the selection of resistant weeds and microbial groups capable of greater proliferation after chemical treatments. It is therefore necessary in the future, to investigate in all these directions to better understand the resistance phenomenon and therefore to implement more targeted control strategies.

Management practices that cause beneficial changes in soil community composition are likely to increase agricultural sustainability. Minimum tillage, cover cropping, organic fertilization and the use of bio-stimulants able to sustain benefit microorganisms can be useful tools for enhancing the biodiversity of soil microbial communities and keeping the problem of herbicide resistance under control. Furthermore, following the assessment of different microbial profiles in soils with different incidences of herbicide resistance, the enrichment of the soil with microorganisms inhibiting the spread of weeds could be of benefit in the context of weed management.

The selection of microorganisms suitable to be associated and the composition of an effective microbial consortium is not easy. It must be based on the understanding of the functionality of the different species and the potential interactions between the different components of the microbial consortium.

The methodology applied in this study and the results obtained can be a useful support for the development of predictive models of the possible evolution of herbicide resistance in relation to microbial composition/diversity in other types of crops, including the vineyard, where recent years have witnessed the evolution of weed populations resistant to the most commonly used herbicides.

Our findings can contribute to planning more targeted and sustainable weed management strategies considering the preservation and enhancement of soil biodiversity, through conservative and regenerative agriculture practices adoption, reducing chemical inputs, improving food health and protecting the environment and human health. In compliance with the Farm to Fork strategy, that provides for a reduction and more sustainable use of pesticides and inputs, regardless of the distribution tool used, the management of resistance phenomena, that can reduce the effectiveness of treatments, is a strategic aspect to be considered.

## Methods

### Study area

The study area is located in the North-western Italy in the Lombardy region (Po Valley) in one of the most important rice-growing vocation territories, characterized by homogeneous climatic and geological conditions with debris, alluvial, fluviolacustrine and fluvioglacial deposits (Supplementary Figure 1).

### Sampling

Soil samples and *E. crus-galli* and *E. oryzicola* specimens that survived herbicide treatments were collected from 32 rice farms managed according to the Directive 2009/128 EC^34^. Samples collection was conducted during summer 2023.

Soil collection was conducted according to the non-systematic X scheme (~ 1 kg of soil obtained from 12 equidistant collections of equal amount along the diagonals of the sampling unit) by Lambkin^65^. Soil samples were aseptically taken from 10 to 25 cm depth. Three different samples, recovered from the same experimental site were pooled and subdivided into aliquots of about 350 mg for metagenomic analysis and stored at − 30°C. Aliquots of about 1 kg for the analysis of physical-chemical characteristics were kept at 4°C.

The presence of *E. crus-galli* and *E. oryzicola* specimens survived herbicide application was recorded by means of cover/abundance values in accordance to Braun-Blanquet^66^ at an equal and random defined sampling unit of about 100 m^2^ extension within each paddy (% coverage of the plant-area projection on the paddy area). The total HeR incidence value was assigned as the midpoint of cover range (according to Braun-Blanquet scale) of the sum of *E. crus-galli* and *E. oryzicola* % coverage.

Sampling units were signed with an identification code. Herbicides applied, classified as mechanism of action (MoA) and following the *Herbicide Resistance Action Committee* (HRAC)^67^, and *Echinochloa* spp. survived chemical control are listed in Table 3. Herbicide resistance was tested according to Cusaro^13^: (i) controlled green-house growth trials were performed applying the same herbicides used in fields; (ii) plants were maintained in a growth chamber with a mean temperature of 20 °C, a relative humidity of 70% and a photoperiod of 14/10 h (day/night); (iii) three weeks after treatment, the sensitivity/resistance of plants to herbicides was tested following European and Mediterranean Plant Protection Organization (EPPO) standards^68^.

**Table 3:** List of herbicides applied and *Echinochloa* spp. survived chemical control in each surveyed paddy.

Farm ID	Herbicide	MoA	HRAC group	*Echinochloa* species
FR.01	Cyhalofop-butyl	ACCase	A	*E. crus-galli*
				*E. oryzicola*
FR.02	Imazamox	ALS	B	*E. crus-galli*
FR.03	Imazamox	ALS	B	*E. oryzicola*
FR.04	Cyhalofop-butyl/Profoxydim+DASH HC	ACCase	A	*E. crus-galli*
FR.05	Imazamox	ALS	B	*E. crus-galli*
				*E. oryzicola*
FR.06	Imazamox	ALS	B	*E. crus-galli*
FR.07	Imazamox	ALS	B	*E. crus-galli*
FR.08	Cyhalofop-butyl/Profoxydim+DASH HC	ACCase	A	*E. oryzicola*
FR.09	Imazamox	ALS	B	*E. oryzicola*
FR.10	Imazamox/Profoxydim	ACCase/ALS	AB	*E. crus-galli*
				*E. oryzicola*
FR.11	Imazamox/Profoxydim	ACCase/ALS	AB	*E. oryzicola*
FR.12	Imazamox/Profoxydim	ACCase/ALS	AB	*E. oryzicola*
FR.13	Profoxydim+DASH HC	ACCase	A	*E. crus-galli*
				*E. oryzicola*
FR.14	Cyhalofop-butyl/Profoxydim+DASH HC	ACCase	A	*E. oryzicola*
FR.15	Cyhalofop-butyl/Profoxydim+DASH HC	ACCase	A	*E. oryzicola*
FR.16	Cyhalofop-butyl/Profoxydim+DASH HC	ACCase	A	*E. oryzicola*
FR.17	Imazamox/Profoxydim	ACCase/ALS	AB	*E. crus-galli*
FR.18	Imazamox+DASH HC/Profoxydim+DASH HC	ACCase/ALS	AB	*E. oryzicola*
FR.19	Clethodim	ACCase	A	*E. oryzicola*
FR.20	Imazamox+DASH HC	ALS	B	*E. oryzicola*
FR.21	Cyhalofop-butyl/Profoxydim+DASH HC	ACCase	A	*E. crus-galli*
FR.22	Penoxsulam	ALS	B	*E. oryzicola*
FR.23	Bispyribac-sodium+Biopower/Clethodim	ACCase/ALS	AB	*E. oryzicola*
FR.24	Clethodim	ACCase	A	*E. oryzicola*
FR.25	Bispyribac-sodium+Biopower	ALS	B	*E. oryzicola*
FR.26	Cyhalofop-butyl	ACCase	A	*E. oryzicola*
FR.27	Imazamox	ALS	B	*E. crus-galli*
FR.28	Cyhalofop-butyl/Profoxydim+DASH HC	ACCase	A	*E. oryzicola*
FR.29	Cyhalofop-butyl/Profoxydim+DASH HC	ACCase	A	*E. crus-galli*
FR.30	Profoxydim+DASH HC	ACCase	A	*E. crus-galli*
FR.31	Cyhalofop-butyl	ACCase	A	*E. oryzicola*
FR.32	Cyhalofop-butyl	ACCase	A	*E. oryzicola*

Farm ID: identification code of each soil sample. MoA: herbicide classification in reference to mechanism of action. HRAC group: herbicide classification in reference to HRAC mode of action classification 2022 map.

### Physical and chemical properties of soil

Physical-chemical properties of soils were determined at “Minoprio Analisi e Certificazioni” (Como, Italy) according to the Italian standard protocols (DM 13/09/99)^69^. The following parameters were evaluated: soil texture (soil composition in coarse and fine sand, silt and clay as g/kg s.s.), pH of H_2_O and pH of CaCl_2_, total and active limestone (CaCO_3_) as g/kg s.s., organic matter and organic carbon (C) as g/kg s.s., total nitrogen (N) as g/kg s.s., cation exchange capacity (CexC) as meq/100g s.s., exchangeable calcium (Ca), magnesium (Mg), sodium (Na) and potassium (K) as meq/100g s.s., base saturation (BS), C/N, Ca/Mg and Mg/K ratios, exchangeable sodium percentage (ESP) and extractable phosphorus (P_ass) as mg/kg s.s.^70^.

### DNA extraction and metagenomic amplicon production

Total DNA was extracted from thawed soil samples using the NucleoSpin Soil kit (Macherey-Nagel, Düren, Germany) according to the manufacturer specifications. DNA was then quantified on a Qubit fluorometer (ThermoFisher Scientific, Waltham, MA). Metagenomic amplicons of bacterial and fungal communities were obtained following PCR amplification, using primers linked to Illumina adapters. To produce bacterial amplicons, the V3-V4 hypervariable region of the prokariotic 16S rRNA gene was targeted, using the primers designed by Takahashi et al.^71^ (Pro341_Forward: 5′-TCGTCGGCAGCGTCAGATGTGTATAAGAGACAG-CCTACGGGNBGCASCAG-3′ and Pro805_Reverse: 5′-GTCTCGTGGGCTCGGAGATGTGTATAAGAGACAG-GACTACNVGGGTATCTAATCC-3′). To obtain fungal amplicons the ribosomal ITS1 region was targeted, by using primers BITS and B58S3 designed by Bokulich and Mills^72^ (BITS_ Forward: 5′-TCGTCGGCAGCGTCAGATGTGTATAAGAGACAG-ACCTGCGGARGGATCA-3′ and B58S3_ Reverse: 5′-GTCTCGTGCTCGAGATGTGTATAAGAGACAG-GAGATCCRTTGYTRAAAGTT-3′).

### Amplicon sequencing and bioinformatics analysis

Sequencing and bioinformatic data analysis were performed at BMR Genomics srl (Padua, Italy). All amplicons were subjected to 2x250 pb pair-end sequencing method using Illumina®'s MiSeq platform following the MiSeq standard operating procedure (SOP) pipeline^73^. Bioinformatic analysis was performed by Qiime2 version 2020.2^74^. Raw bacterial sequences were analyzed through Mothur software^75^ and reads were then subjected to demultiplexing, trimming off of primers and quality control to exclude chimaeras, singletons and short sequences. High-quality reads were aligned against the SILVA database^76^ for annotation of *Bacteria* and *Archaea* and clustered into Operational Taxonomic Units (OTUs) at 99% homology. Taxonomic attribution of OTUs was obtained against the curated database RDP Bayesian Classifier^77^, using as reference the GenBank database^78^ and the Greengenes reference collection of annotated sequences^79^.

As concerns *Fungi*, data analysis was performed using the USEARCH-based ITS pipeline. De-novoUPARSE-OUT^80^ algorithm was used to pick the OTUs at 99% of similarity and to remove chimeras. OTUs were identified against UNITE database (version: 9.0; Last updated: 2023-08-01)^81^, collected in the .biom file and filtered at 0.005% abundance (Bokulich et al.^72^) to eliminate spurious OTUs that are present at low frequency.

### Statistical analysis

Relative abundance of all microbial taxa with a prevalence ≥ 5% was considered and statistical analysis was performed at the taxonomic rank of orders using R version 4.3.0 (updated in April 21th, 2023) (R Core Team^82^).

α-diversity was assessed by computing Margalef, Shannon, Simpson and Pielou indexes using the function “diversity” (vegan package)^83^.

To assess β-diversity, bootstrapped hierarchical clustering was performed for the incidence of hebicide resistance (HeR) and soil physical-chemical properties (“canberra” distance - “ward.D2” algorithm) and for HeR and soil microbial communities (“Bray-Curtis” distance - “ward.D2” algorithm) using the function “vegdist” (vegan package)^83^ and “pvclust” (pvclust package)^84^. Heatmaps were graphed using the function “heatmap.2” (gplots package)^85^.

Using the function “envfit” (vegan package)^83^, envfit analyses were performed in order to investigate whether soil microbial communities or soil physical-chemical properties significantly contributed to the clustering of farms.

Principal Coordinates Analysis (PCoA) was plotted using the functions “metaMDS” (vegan package)^83^ and “ggplot” (ggplot2 package)^86^ in order to analyze the relation between the most contributive microbial orders and incidence of HeR.

To investigate the relationships between HeR and soil microbial communities composition, between HeR and soil physical-chemical properties, between soil microbial communities composition and soil physical-chemical properties, redundancy analyses (RDA) were performed using the function “rda” (vegan package)^83^. To test significant effects of investigated variables, constrained correspondence analyses were performed using the function “anova.cca” (vegan package)^83^. RDA biplot was graphed using the function “ggord” (ggord package)^87^.

Using the function “wilcox.test” (stats package)^82^ differences in the abundance of microbial orders between H-HeR and L-HeR soils were analyzed by Mann-Whitney test (two tailed). Barplots were graphed using the function “ggplot” (ggplot2 package)^86^.

Maps were generated using QGIS software, version 3.32 Lima^88^ and consulting SIARL 2012-2019^89^, DUSAF 7.0^90^ and Geoportale Nazionale^91^ database.

All the methods were carried out in accordance with relevant Institutional guidelines and regulations.

### Supplementary Information

Supplementary Information.

## Data Availability

The datasets generated and/or analysed during the current study are openly available in the NCBI Sequence Read Archive (SRA) under the BioProject Number PRJNA1041979.
