# Supplementary material for: Incidence of resistance to ALS and ACCase inhibitors in Echinochloa species and soil microbial composition in Northern Italy
Source: Sci Rep. 2024 May 8;14:10544. doi: 10.1038/s41598-024-59856-0 (PMC11078947; doi:10.1038/s41598-024-59856-0)
Supplement: Supplementary file 1 — Supplementary Information. [file 41598_2024_59856_MOESM1_ESM.doc]

**HERBICIDE RESISTANCE AND SOIL MICROBIAL COMPOSITION**

**SUPPLEMENTARY MATERIAL**

**Carlo Maria Cusaro**^+^**, Enrica Capelli**^+^**, Anna Maria Picco**+ **and Maura Brusoni**^+*^

Department of Earth and Environmental Sciences, University of Pavia, Pavia, 27100, Italy.

*maura.brusoni@unipv.it

+these authors contributed equally to this work

| Order | FR.1 | FR.2 | FR.3 | FR.4 | FR.5 | FR.6 | FR.7 | FR.8 | FR.9 | FR.10 | FR.11 | FR.12 | FR.13 | FR.14 | FR.15 | FR.16 | FR.17 | FR.18 | FR.19 | FR.20 | FR.21 | FR.22 | FR.23 | FR.24 | FR.25 | FR.26 | FR.27 | FR.28 | FR.29 | FR.30 | FR.31 | FR.32 |
| --- | --- | --- | --- | --- | --- | --- | --- | --- | --- | --- | --- | --- | --- | --- | --- | --- | --- | --- | --- | --- | --- | --- | --- | --- | --- | --- | --- | --- | --- | --- | --- | --- |
| *Actinomycetales* | 5,717 | 1,869 | 3,048 | 8,359 | 10,113 | 7,666 | 9,382 | 10,334 | 11,943 | 7,352 | 32,179 | 13,163 | 13,065 | 8,000 | 4,718 | 13,072 | 8,187 | 7,625 | 35,464 | 12,465 | 6,925 | 6,940 | 15,577 | 8,159 | 7,176 | 7,784 | 18,431 | 16,264 | 6,984 | 4,551 | 6,916 | 17,472 |
| *Rhizobiales* | 3,677 | 6,184 | 11,491 | 6,990 | 13,703 | 4,944 | 2,686 | 7,265 | 8,431 | 3,783 | 5,886 | 10,593 | 9,087 | 8,297 | 4,950 | 8,715 | 1,754 | 4,630 | 0,498 | 7,503 | 8,963 | 7,520 | 6,899 | 5,915 | 10,025 | 13,135 | 9,207 | 11,223 | 8,125 | 9,014 | 10,781 | 8,404 |
| *N.I. Bacteria and Archaea* | 5,910 | 7,060 | 6,932 | 5,261 | 4,812 | 6,188 | 4,565 | 6,543 | 5,849 | 6,347 | 14,303 | 10,804 | 6,635 | 7,978 | 6,613 | 3,609 | 7,895 | 7,703 | 1,923 | 4,397 | 5,481 | 6,681 | 4,645 | 5,558 | 7,176 | 8,187 | 8,731 | 4,945 | 7,035 | 7,774 | 8,992 | 7,274 |
| *Methanosarcinales* | 3,850 | 10,720 | 12,057 | 1,189 | 1,723 | 3,233 | 0,185 | 0,000 | 0,650 | 0,140 | 0,234 | 10,456 | 3,120 | 0,000 | 15,073 | 0,308 | 0,000 | 1,310 | 0,000 | 9,829 | 1,290 | 6,632 | 4,203 | 8,648 | 5,475 | 2,661 | 2,459 | 0,000 | 3,475 | 4,857 | 11,441 | 1,887 |
| *Clostridiales* | 1,829 | 2,774 | 2,176 | 7,941 | 1,189 | 0,489 | 8,375 | 1,940 | 4,092 | 1,153 | 3,495 | 6,452 | 1,226 | 0,000 | 1,296 | 0,528 | 3,216 | 0,774 | 1,244 | 7,919 | 2,695 | 6,258 | 5,279 | 3,405 | 5,968 | 7,825 | 4,119 | 1,851 | 5,655 | 2,786 | 2,985 | 2,989 |
| *Bacillales* | 1,771 | 3,545 | 1,358 | 4,367 | 6,601 | 2,711 | 1,874 | 1,444 | 3,715 | 1,389 | 6,068 | 6,087 | 2,555 | 0,737 | 1,325 | 6,008 | 0,585 | 0,117 | 4,468 | 2,060 | 7,867 | 1,925 | 3,682 | 4,755 | 1,571 | 3,513 | 3,051 | 9,476 | 3,560 | 3,486 | 1,464 | 4,610 |
| *envOPS12* | 8,778 | 7,446 | 2,500 | 0,058 | 0,189 | 2,577 | 0,443 | 6,769 | 5,076 | 1,729 | 0,917 | 0,608 | 1,637 | 0,583 | 6,091 | 1,496 | 3,509 | 5,205 | 0,000 | 3,415 | 1,290 | 3,995 | 2,039 | 5,417 | 4,690 | 2,463 | 2,441 | 1,404 | 2,070 | 1,677 | 3,042 | 1,442 |
| *Nitrospirales* | 2,830 | 1,734 | 6,267 | 1,693 | 1,778 | 2,244 | 0,000 | 2,572 | 0,913 | 1,766 | 0,323 | 0,632 | 1,595 | 2,113 | 3,026 | 1,673 | 16,667 | 1,753 | 1,199 | 1,238 | 2,412 | 4,001 | 1,745 | 1,516 | 4,338 | 2,072 | 0,682 | 1,994 | 2,785 | 6,199 | 1,626 | 0,392 |
| *iii1-15* | 2,541 | 2,485 | 2,545 | 0,721 | 0,545 | 1,222 | 1,533 | 6,634 | 3,609 | 2,815 | 1,297 | 1,953 | 3,412 | 8,506 | 1,083 | 3,543 | 2,339 | 4,679 | 0,984 | 0,576 | 0,800 | 1,080 | 2,628 | 1,309 | 1,688 | 1,756 | 1,786 | 3,900 | 1,286 | 2,655 | 1,253 | 2,259 |
| *Sphingomonadales* | 1,501 | 1,397 | 0,270 | 1,030 | 2,901 | 4,300 | 0,049 | 2,437 | 3,715 | 2,638 | 0,901 | 1,029 | 0,909 | 5,612 | 1,247 | 11,466 | 0,000 | 4,075 | 0,464 | 1,622 | 2,051 | 0,495 | 3,002 | 2,775 | 1,231 | 0,461 | 1,454 | 3,278 | 1,482 | 0,438 | 0,459 | 3,873 |
| *Gaiellales* | 0,885 | 0,732 | 0,944 | 2,457 | 0,222 | 0,878 | 1,810 | 1,670 | 2,037 | 2,874 | 1,198 | 6,322 | 5,306 | 3,708 | 0,938 | 2,267 | 0,000 | 1,743 | 0,667 | 0,993 | 1,728 | 0,537 | 2,402 | 0,762 | 0,692 | 3,163 | 4,092 | 4,443 | 2,623 | 3,544 | 1,540 | 2,644 |
| *GCA004* | 2,541 | 2,899 | 3,552 | 0,094 | 0,133 | 1,655 | 0,107 | 1,038 | 3,522 | 2,268 | 1,188 | 0,324 | 2,417 | 0,000 | 1,682 | 0,242 | 6,140 | 2,887 | 1,335 | 4,280 | 0,000 | 4,496 | 2,005 | 2,212 | 3,271 | 2,807 | 3,275 | 0,838 | 2,453 | 0,496 | 2,305 | 1,681 |
| *Myxococcales* | 0,635 | 0,934 | 1,861 | 3,344 | 0,945 | 2,178 | 1,713 | 2,256 | 2,661 | 5,630 | 1,255 | 2,253 | 1,612 | 0,990 | 1,276 | 4,665 | 0,877 | 3,170 | 0,000 | 1,291 | 1,070 | 0,869 | 1,359 | 1,292 | 1,149 | 2,264 | 3,168 | 2,249 | 1,703 | 1,400 | 1,923 | 3,268 |
| *Solibacterales* | 1,501 | 2,398 | 1,717 | 5,066 | 1,434 | 4,544 | 0,273 | 0,496 | 1,300 | 4,330 | 0,396 | 0,835 | 0,952 | 1,827 | 2,649 | 3,741 | 0,000 | 1,251 | 0,611 | 1,430 | 5,481 | 1,123 | 0,861 | 2,129 | 2,579 | 1,249 | 1,364 | 1,149 | 1,473 | 3,924 | 0,650 | 2,000 |
| *Anaerolineales* | 1,347 | 2,447 | 2,041 | 3,690 | 1,089 | 3,533 | 0,146 | 1,625 | 1,660 | 3,576 | 0,750 | 0,365 | 0,952 | 0,176 | 3,162 | 0,000 | 2,047 | 1,826 | 0,000 | 1,430 | 2,192 | 2,161 | 1,042 | 3,057 | 1,348 | 2,731 | 1,732 | 0,335 | 4,403 | 0,802 | 2,937 | 2,837 |
| *Xanthomonadales* | 2,733 | 0,992 | 1,007 | 2,681 | 5,012 | 2,178 | 0,983 | 6,498 | 1,906 | 1,574 | 0,974 | 1,272 | 1,192 | 3,983 | 1,267 | 3,455 | 0,000 | 1,641 | 0,000 | 1,281 | 2,102 | 0,779 | 1,178 | 1,284 | 0,821 | 1,144 | 0,790 | 3,278 | 0,724 | 0,321 | 1,282 | 1,787 |
| *Saprospirales* | 2,214 | 1,686 | 0,890 | 1,874 | 2,945 | 6,144 | 0,000 | 1,850 | 0,975 | 1,892 | 0,766 | 0,778 | 0,111 | 2,718 | 0,986 | 3,455 | 0,000 | 2,269 | 0,679 | 2,743 | 2,192 | 1,823 | 3,444 | 3,910 | 2,544 | 0,572 | 0,817 | 1,819 | 0,750 | 0,540 | 1,157 | 1,442 |
| *Desulfurococcales* | 0,000 | 1,676 | 5,736 | 1,030 | 0,333 | 3,444 | 0,000 | 0,000 | 0,000 | 0,598 | 0,047 | 2,553 | 0,574 | 0,000 | 4,912 | 0,000 | 0,000 | 0,794 | 0,090 | 3,074 | 0,181 | 4,617 | 0,974 | 3,819 | 4,455 | 1,663 | 0,718 | 0,000 | 1,899 | 7,964 | 3,683 | 0,698 |
| *Acidobacteriales* | 0,154 | 2,302 | 0,566 | 7,206 | 0,633 | 5,588 | 0,355 | 0,542 | 0,422 | 2,557 | 0,557 | 0,875 | 0,283 | 0,033 | 2,378 | 2,883 | 0,000 | 0,282 | 0,000 | 0,683 | 7,906 | 0,531 | 0,125 | 1,193 | 1,512 | 1,354 | 1,184 | 0,654 | 0,920 | 4,638 | 1,033 | 1,548 |
| *Rhodospirillales* | 2,753 | 0,819 | 0,926 | 1,211 | 2,378 | 1,055 | 0,920 | 3,159 | 2,397 | 1,928 | 0,766 | 0,316 | 7,355 | 1,034 | 1,267 | 0,242 | 0,000 | 3,072 | 0,000 | 0,427 | 0,864 | 1,177 | 0,963 | 0,712 | 0,469 | 1,558 | 3,320 | 2,576 | 0,545 | 2,334 | 1,559 | 1,056 |
| *Bacteroidales* | 0,808 | 1,531 | 3,075 | 0,807 | 0,122 | 0,800 | 16,463 | 0,000 | 0,553 | 3,694 | 1,193 | 0,584 | 0,197 | 0,000 | 2,098 | 0,110 | 5,848 | 0,497 | 0,294 | 1,014 | 0,039 | 1,835 | 0,476 | 1,035 | 0,668 | 1,015 | 0,206 | 0,000 | 1,065 | 0,817 | 1,043 | 0,638 |
| *Acidimicrobiales* | 1,116 | 0,578 | 0,539 | 0,699 | 1,089 | 0,311 | 0,866 | 1,354 | 1,493 | 1,300 | 1,688 | 2,902 | 4,235 | 5,029 | 0,793 | 0,264 | 0,000 | 2,064 | 0,000 | 1,003 | 0,193 | 0,754 | 0,929 | 0,696 | 0,539 | 1,896 | 3,186 | 1,970 | 1,559 | 1,590 | 1,751 | 2,664 |
| *AK31* | 14,937 | 1,975 | 0,000 | 1,838 | 0,278 | 0,278 | 0,161 | 3,926 | 1,792 | 0,776 | 0,000 | 0,219 | 0,334 | 2,619 | 0,396 | 0,638 | 0,000 | 0,370 | 0,000 | 0,352 | 3,585 | 0,024 | 3,999 | 1,185 | 0,375 | 0,035 | 0,144 | 2,090 | 0,000 | 0,117 | 0,105 | 0,146 |
| *SJA-15* | 0,558 | 0,510 | 1,744 | 1,095 | 0,067 | 1,189 | 0,287 | 0,812 | 2,626 | 1,130 | 0,443 | 1,054 | 1,277 | 0,000 | 0,977 | 0,000 | 0,000 | 1,948 | 1,459 | 2,423 | 0,258 | 2,142 | 0,181 | 0,845 | 1,864 | 2,614 | 2,620 | 0,040 | 5,332 | 0,365 | 2,143 | 2,418 |
| *SC-I-84* | 0,269 | 1,194 | 1,636 | 1,838 | 0,333 | 1,244 | 0,112 | 0,812 | 1,335 | 2,564 | 0,651 | 0,689 | 1,432 | 1,574 | 1,083 | 1,144 | 0,000 | 2,284 | 0,000 | 1,996 | 1,122 | 0,827 | 1,065 | 1,127 | 0,715 | 1,791 | 1,490 | 1,260 | 1,959 | 1,211 | 2,277 | 0,731 |
| *RB41* | 0,962 | 4,855 | 0,387 | 0,440 | 0,867 | 2,055 | 0,331 | 0,587 | 1,941 | 1,367 | 0,255 | 0,227 | 0,703 | 2,498 | 2,050 | 2,487 | 1,462 | 1,539 | 0,000 | 1,217 | 0,774 | 0,549 | 1,518 | 1,516 | 1,055 | 0,158 | 0,476 | 2,313 | 0,588 | 0,219 | 0,182 | 1,428 |
| *Rickettsiales* | 0,115 | 0,000 | 0,000 | 0,000 | 0,044 | 0,000 | 0,000 | 0,271 | 0,000 | 0,059 | 0,057 | 0,138 | 0,000 | 0,000 | 0,077 | 0,000 | 0,000 | 0,000 | 35,645 | 0,000 | 0,000 | 0,000 | 0,000 | 0,000 | 0,000 | 0,012 | 0,000 | 0,000 | 0,000 | 0,219 | 0,000 | 0,000 |
| *Burkholderiales* | 1,540 | 0,250 | 0,171 | 1,203 | 2,189 | 1,455 | 1,027 | 3,791 | 1,098 | 2,298 | 0,943 | 0,365 | 1,183 | 1,430 | 1,035 | 1,629 | 0,000 | 1,383 | 0,000 | 1,622 | 0,374 | 0,543 | 1,801 | 1,044 | 0,610 | 0,280 | 0,251 | 1,276 | 0,809 | 0,248 | 0,488 | 1,561 |
| *Pseudanabaenales* | 0,808 | 0,000 | 0,081 | 0,735 | 7,113 | 0,233 | 0,170 | 0,361 | 0,211 | 1,766 | 0,250 | 0,057 | 0,540 | 0,000 | 0,309 | 5,106 | 0,000 | 0,034 | 0,000 | 0,000 | 1,780 | 2,861 | 0,034 | 1,441 | 0,317 | 0,368 | 0,269 | 0,351 | 5,672 | 0,000 | 1,808 | 0,219 |
| *Solirubrobacterales* | 0,000 | 0,106 | 0,270 | 0,886 | 0,244 | 0,222 | 1,591 | 0,000 | 1,431 | 0,842 | 0,750 | 3,047 | 4,998 | 2,168 | 0,338 | 0,000 | 0,000 | 0,940 | 0,000 | 0,491 | 0,129 | 0,706 | 0,329 | 0,306 | 0,340 | 1,447 | 2,872 | 0,949 | 0,852 | 1,269 | 1,224 | 1,980 |
| *Lactobacillales* | 0,000 | 0,039 | 0,000 | 0,029 | 0,056 | 0,056 | 6,278 | 1,354 | 0,070 | 0,000 | 3,578 | 0,146 | 0,120 | 0,242 | 0,329 | 0,154 | 3,216 | 0,175 | 4,038 | 0,171 | 0,387 | 0,066 | 0,181 | 0,000 | 0,000 | 0,163 | 0,135 | 0,136 | 0,128 | 0,058 | 0,105 | 0,106 |
| *Enterobacteriales* | 0,000 | 0,000 | 0,000 | 0,000 | 0,000 | 0,044 | 0,000 | 0,000 | 0,000 | 0,000 | 2,932 | 0,000 | 0,000 | 0,000 | 0,000 | 0,000 | 17,251 | 0,019 | 0,000 | 0,000 | 0,000 | 0,018 | 0,532 | 0,000 | 0,000 | 0,000 | 0,000 | 0,024 | 0,000 | 0,000 | 0,000 | 0,000 |
| *Neisseriales* | 0,000 | 0,000 | 0,000 | 0,000 | 0,000 | 0,000 | 15,139 | 0,090 | 0,000 | 0,074 | 0,599 | 0,000 | 0,000 | 0,000 | 0,058 | 0,000 | 0,000 | 0,000 | 0,000 | 0,000 | 0,077 | 0,000 | 0,000 | 0,000 | 0,000 | 0,064 | 0,000 | 0,000 | 0,000 | 0,000 | 0,000 | 0,000 |
| *Other Bacteria and Archaea* | 17,305 | 17,039 | 16,876 | 11,141 | 19,371 | 12,199 | 12,103 | 16,877 | 16,932 | 13,699 | 9,167 | 9,086 | 17,000 | 17,000 | 15,247 | 10,915 | 9,064 | 19,749 | 3,790 | 13,223 | 16,353 | 18,944 | 13,980 | 15,258 | 16,602 | 16,566 | 10,660 | 12,714 | 17,077 | 14,484 | 16,290 | 12,788 |
| *N.I. Fungi* | 62,868 | 70,923 | 47,805 | 16,064 | 23,778 | 60,554 | 5,446 | 2,427 | 6,141 | 18,917 | 15,836 | 4,896 | 42,392 | 27,256 | 26,996 | 27,737 | 3,984 | 4,035 | 4,237 | 24,345 | 43,210 | 22,229 | 23,099 | 22,280 | 26,681 | 10,943 | 44,452 | 4,472 | 31,082 | 31,246 | 38,095 | 21,326 |
| *Malasseziales* | 0,123 | 0,781 | 0,479 | 0,351 | 0,027 | 1,079 | 83,451 | 92,083 | 68,175 | 33,626 | 31,798 | 19,225 | 1,559 | 0,513 | 11,154 | 42,298 | 78,657 | 79,859 | 80,628 | 0,359 | 0,000 | 1,748 | 0,945 | 1,825 | 0,592 | 26,595 | 13,494 | 8,428 | 11,205 | 1,634 | 0,839 | 10,914 |
| *Agaricales* | 31,105 | 1,324 | 14,808 | 1,422 | 64,853 | 1,863 | 0,000 | 0,000 | 0,275 | 9,369 | 4,407 | 7,604 | 0,494 | 11,750 | 1,826 | 0,000 | 0,000 | 0,392 | 0,542 | 7,366 | 15,741 | 9,186 | 1,674 | 0,690 | 24,887 | 1,328 | 0,930 | 18,882 | 1,147 | 0,241 | 9,632 | 2,633 |
| *Sordariales* | 0,000 | 0,519 | 14,050 | 51,539 | 0,160 | 2,935 | 0,000 | 0,000 | 0,000 | 2,294 | 3,013 | 14,199 | 0,602 | 25,311 | 1,707 | 1,067 | 0,000 | 0,868 | 0,000 | 2,224 | 0,000 | 2,430 | 1,762 | 6,476 | 5,051 | 1,509 | 7,784 | 1,154 | 0,941 | 22,072 | 2,329 | 9,574 |
| *Pezizales* | 0,000 | 3,952 | 1,417 | 0,000 | 0,000 | 0,000 | 0,000 | 0,000 | 0,000 | 0,433 | 0,747 | 0,158 | 0,000 | 4,754 | 0,170 | 0,476 | 0,000 | 0,000 | 0,000 | 0,081 | 22,222 | 0,000 | 2,237 | 0,204 | 0,000 | 4,226 | 0,000 | 42,853 | 0,000 | 5,065 | 15,787 | 21,541 |
| *Diaporthales* | 0,035 | 0,042 | 0,000 | 0,000 | 0,000 | 0,000 | 10,738 | 4,023 | 9,599 | 5,596 | 5,005 | 1,123 | 0,108 | 0,000 | 1,758 | 3,368 | 17,133 | 10,102 | 14,428 | 0,000 | 0,000 | 0,341 | 0,108 | 0,000 | 0,000 | 5,329 | 1,265 | 1,270 | 1,545 | 0,114 | 0,000 | 1,053 |
| *Mortierellales* | 1,389 | 0,687 | 0,702 | 0,068 | 1,653 | 1,288 | 0,365 | 0,000 | 0,537 | 3,442 | 4,009 | 0,907 | 0,000 | 4,052 | 13,498 | 1,471 | 0,068 | 0,361 | 0,164 | 1,317 | 5,864 | 0,804 | 1,434 | 1,086 | 16,911 | 0,586 | 1,328 | 14,860 | 0,000 | 10,928 | 1,641 | 0,000 |
| *Eurotiales* | 0,473 | 1,016 | 0,667 | 1,074 | 0,453 | 1,484 | 0,000 | 0,000 | 0,729 | 0,000 | 2,789 | 42,180 | 4,290 | 0,000 | 0,000 | 0,620 | 0,000 | 0,000 | 0,000 | 1,192 | 0,000 | 4,005 | 2,731 | 1,495 | 2,464 | 6,364 | 3,939 | 1,016 | 0,895 | 3,121 | 1,087 | 1,436 |
| *Venturiales* | 0,000 | 0,000 | 1,118 | 25,620 | 0,053 | 0,713 | 0,000 | 0,000 | 0,000 | 0,000 | 0,000 | 0,000 | 0,000 | 0,000 | 0,501 | 0,137 | 0,000 | 0,000 | 0,000 | 1,053 | 0,000 | 0,225 | 0,142 | 1,363 | 0,932 | 0,906 | 0,000 | 0,682 | 0,054 | 1,862 | 0,575 | 0,000 |
| *Pleosporales* | 0,132 | 0,385 | 0,431 | 0,268 | 1,040 | 0,451 | 0,000 | 0,000 | 0,000 | 0,000 | 0,000 | 3,399 | 0,000 | 17,666 | 0,000 | 0,000 | 0,000 | 0,000 | 0,000 | 0,388 | 0,000 | 0,244 | 0,724 | 1,825 | 2,116 | 0,000 | 1,081 | 0,109 | 0,237 | 2,818 | 0,316 | 0,503 |
| *Helotiales* | 0,197 | 0,042 | 0,674 | 0,651 | 0,000 | 1,288 | 0,000 | 0,831 | 0,000 | 0,000 | 0,000 | 0,000 | 0,000 | 0,000 | 0,000 | 0,426 | 0,158 | 0,350 | 0,000 | 0,607 | 0,000 | 0,566 | 10,607 | 0,684 | 2,560 | 0,000 | 1,518 | 0,065 | 0,405 | 0,867 | 0,264 | 0,000 |
| *Sebacinales* | 0,000 | 0,000 | 0,000 | 0,000 | 0,000 | 0,000 | 0,000 | 0,000 | 0,000 | 0,000 | 0,000 | 0,000 | 0,000 | 0,000 | 0,000 | 0,000 | 0,000 | 0,000 | 0,000 | 0,000 | 0,000 | 0,000 | 20,186 | 0,000 | 0,000 | 0,000 | 0,000 | 0,000 | 0,000 | 0,000 | 0,000 | 0,000 |
| *Other Fungi* | 0,478 | 3,920 | 1,459 | 0,112 | 0,178 | 0,889 | 0,000 | 0,000 | 0,000 | 0,000 | 0,000 | 0,821 | 0,000 | 6,348 | 1,232 | 0,361 | 0,000 | 0,000 | 0,000 | 2,114 | 2,778 | 0,926 | 1,185 | 1,212 | 1,132 | 0,871 | 0,974 | 0,349 | 0,000 | 1,235 | 2,609 | 1,532 |

**Supplementary table 1**: relative abundance of bacterial, archaeal and fungal orders identified in the surveyed farms.

| Orders | NMDS1 | NMDS2 | R2 | P |
| --- | --- | --- | --- | --- |
| *Actinomycetales* | 0.864 | -0.503 | 0.272 | 0.011 * |
| *Rhizobiales* | -0.968 | 0.251 | 0.286 | 0.008 ** |
| *Methanosarcinales* | -0.989 | -0.148 | 0.215 | 0.018 * |
| *Clostridiales* | 0.041 | -0.999 | 0.119 | 0.149 |
| *Bacillales* | -0.381 | 0.925 | 0.022 | 0.721 |
| *envOPS12* | -0.127 | 0.992 | 0.120 | 0.137 |
| *Nitrospirales* | 0.344 | 0.939 | 0.042 | 0.522 |
| *iii1-15* | 0.397 | -0.918 | 0.015 | 0.806 |
| *Sphingomonadales* | -0.013 | 1.000 | 0.005 | 0.915 |
| *Gaiellales* | -0.090 | -0.996 | 0.232 | 0.012 * |
| *GCA004* | 0.421 | 0.907 | 0.037 | 0.592 |
| *Myxococcales* | 0.427 | -0.904 | 0.056 | 0.402 |
| *Solibacterales* | -0.959 | -0.285 | 0.220 | 0.028 * |
| *Anaerolineales* | -0.989 | -0.150 | 0.063 | 0.358 |
| *Xanthomonadales* | -0.223 | 0.975 | 0.102 | 0.220 |
| *Saprospirales* | -0.835 | 0.551 | 0.164 | 0.076 |
| *Desulfurococcales* | -0.890 | -0.457 | 0.239 | 0.021 * |
| *Acidobacteriales* | -0.775 | -0.632 | 0.162 | 0.069 |
| *Rhodospirillales* | -0.749 | 0.662 | 0.005 | 0.923 |
| *Bacteroidales* | 1.000 | -0.029 | 0.171 | 0.084 |
| *Acidimicrobiales* | -0.262 | -0.965 | 0.154 | 0.082 |
| *AK31* | -0.389 | 0.921 | 0.139 | 0.130 |
| *SJA-15* | 0.063 | -0.998 | 0.014 | 0.800 |
| *SC-I-84* | -0.638 | -0.770 | 0.152 | 0.080 |
| *RB41* | -0.567 | 0.823 | 0.053 | 0.452 |
| *Rickettsiales* | 0.995 | -0.098 | 0.187 | 0.074 |
| *Burkholderiales* | 0.167 | 0.986 | 0.036 | 0.614 |
| *Pseudanabaenales* | -0.165 | 0.986 | 0.190 | 0.05 * |
| *Solirubrobacterales* | -0.047 | -0.999 | 0.239 | 0.014 * |
| *Lactobacillales* | 0.994 | -0.106 | 0.522 | 0.001 *** |
| *Enterobacteriales* | 0.938 | 0.346 | 0.168 | 0.095 |
| *Neisseriales* | 0.971 | -0.239 | 0.140 | 0.137 |
| *Malasseziales* | 0.998 | 0.064 | 0.875 | 0.001 *** |
| *Agaricales* | -0.320 | 0.947 | 0.603 | 0.001 *** |
| *Sordariales* | -0.325 | -0.946 | 0.543 | 0.001 *** |
| *Pezizales* | -0.344 | 0.939 | 0.118 | 0.167 |
| *Diaporthales* | 0.994 | 0.107 | 0.823 | 0.001 *** |
| *Mortierellales* | -0.476 | 0.879 | 0.164 | 0.084 |
| *Eurotiales* | -0.035 | -0.999 | 0.138 | 0.136 |
| *Venturiales* | -0.251 | -0.968 | 0.238 | 0.016 * |
| *Pleosporales* | -0.571 | -0.821 | 0.188 | 0.067 |
| *Helotiales* | -0.362 | -0.932 | 0.064 | 0.393 |
| *Sebacinales* | -0.204 | -0.979 | 0.046 | 0.450 |

**Supplementary table 2**: results of the envfit analysis of bacterial, archaeal and fungal orders. Df: degrees of freedom. **: P < 0.01.

| Orders | Df | Variance | F | P |
| --- | --- | --- | --- | --- |
| *Actinomycetales* | 1 | 12.134 | 1.210 | 0.283 |
| *Rhizobiales* | 1 | 137.111 | 13.674 | 0.001 *** |
| *Methanosarcinales* | 1 | 34.053 | 3.396 | 0.061 |
| *Gaiellales* | 1 | 20.879 | 2.082 | 0.161 |
| *Solibacterales* | 1 | 68.421 | 6.824 | 0.005 ** |
| *Desulfurococcales* | 1 | 8.685 | 0.866 | 0.396 |
| *Pseudanabaenales* | 1 | 30.042 | 2.996 | 0.08 |
| *Solirubrobacterales* | 1 | 11.142 | 1.111 | 0.341 |
| *Lactobacillales* | 1 | 120.054 | 11.973 | 0.001 *** |
| *Malasseziales* | 1 | 122.125 | 12.180 | 0.003 ** |
| *Agaricales* | 1 | 9.249 | 0.922 | 0.398 |
| *Sordariales* | 1 | 12.587 | 1.255 | 0.283 |
| *Diaporthales* | 1 | 85.803 | 8.557 | 0.002 ** |
| *Venturiales* | 1 | 11.206 | 1.118 | 0.31 |
| *Residual* | 17 | 170.458 |  |  |

**Supplementary table 3**: results of the constrained correspondence analysis between HeR and bacterial, archaeal and fungal communities. Df: degrees of freedom. F: value of constrained correspondence analysis statistics. **: P < 0.01.

|  |  |  | L-HeR | | | | | | | | | |  | H-HeR | | | | | |
| --- | --- | --- | --- | --- | --- | --- | --- | --- | --- | --- | --- | --- | --- | --- | --- | --- | --- | --- | --- |
| Order | Genus | Species | FR.1 | FR.2 | FR.10 | FR.13 | FR.15 | FR.16 | FR.27 | FR.28 | FR.31 | FR.32 |  | FR.7 | FR.8 | FR.9 | FR.17 | FR.18 | FR.19 |
| *Acidimicrobiales* |  |  | 1.12 | 0.58 | 1.30 | 3.96 | 0.79 | 0.26 | 3.19 | 1.97 | 1.75 | 2.60 |  | 0.87 | 1.35 | 1.24 | 0.00 | 1.91 | 0.00 |
|  | *Iamia* |  | 0.00 | 0.00 | 0.00 | 0.27 | 0.00 | 0.00 | 0.00 | 0.00 | 0.00 | 0.07 |  | 0.00 | 0.00 | 0.25 | 0.00 | 0.15 | 0.00 |
|  | *Ilumatobacter* | *fluminis* | 0.00 | 0.00 | 0.00 | 0.00 | 0.00 | 0.00 | 0.00 | 0.00 | 0.00 | 0.00 |  | 0.00 | 0.00 | 0.00 | 0.00 | 0.00 | 0.00 |
| *Acidobacteriales* |  |  | 0.00 | 2.30 | 1.04 | 0.21 | 2.06 | 1.74 | 0.71 | 0.53 | 0.77 | 1.20 |  | 0.36 | 0.00 | 0.00 | 0.00 | 0.12 | 0.00 |
|  | *Candidatus Koribacter* |  | 0.15 | 0.00 | 1.29 | 0.07 | 0.00 | 1.14 | 0.17 | 0.12 | 0.27 | 0.35 |  | 0.00 | 0.54 | 0.23 | 0.00 | 0.17 | 0.00 |
|  | *Candidatus Koribacter* | *versatilis* | 0.00 | 0.00 | 0.22 | 0.00 | 0.32 | 0.00 | 0.31 | 0.00 | 0.00 | 0.00 |  | 0.00 | 0.00 | 0.19 | 0.00 | 0.00 | 0.00 |
|  | *Edaphobacter* |  | 0.00 | 0.00 | 0.00 | 0.00 | 0.00 | 0.00 | 0.00 | 0.00 | 0.00 | 0.00 |  | 0.00 | 0.00 | 0.00 | 0.00 | 0.00 | 0.00 |
| *Actinomycetales* |  |  | 2.68 | 0.32 | 4.35 | 8.37 | 1.96 | 7.20 | 10.57 | 6.27 | 2.54 | 7.06 |  | 0.95 | 4.60 | 6.87 | 0.00 | 4.93 | 1.49 |
|  | *Actinomadura* | *nitritigenes* | 0.00 | 0.00 | 0.00 | 0.00 | 0.00 | 0.00 | 0.00 | 0.00 | 0.00 | 0.00 |  | 0.00 | 0.00 | 0.00 | 0.00 | 0.00 | 0.00 |
|  | *Actinomadura* | *vinacea* | 0.00 | 0.00 | 0.11 | 0.00 | 0.00 | 0.00 | 0.00 | 0.00 | 0.00 | 0.00 |  | 0.00 | 0.00 | 0.00 | 0.00 | 0.00 | 0.00 |
|  | *Actinomyces* |  | 0.00 | 0.00 | 0.00 | 0.00 | 0.00 | 0.00 | 0.00 | 0.00 | 0.00 | 0.00 |  | 0.14 | 0.00 | 0.00 | 0.00 | 0.00 | 0.00 |
|  | *Actinomycetospora* |  | 0.00 | 0.00 | 0.00 | 0.00 | 0.00 | 0.00 | 0.00 | 0.00 | 0.00 | 0.00 |  | 0.00 | 0.00 | 0.00 | 0.00 | 0.00 | 0.33 |
|  | *Actinoplanes* |  | 0.00 | 0.00 | 0.00 | 0.00 | 0.00 | 0.00 | 0.00 | 0.00 | 0.00 | 0.13 |  | 0.00 | 0.00 | 0.00 | 0.00 | 0.00 | 0.00 |
|  | *Agromyces* |  | 0.25 | 0.00 | 0.00 | 0.17 | 0.00 | 0.00 | 0.00 | 0.17 | 0.00 | 0.00 |  | 0.00 | 0.00 | 0.00 | 0.00 | 0.12 | 0.00 |
|  | *Amycolatopsis* |  | 0.00 | 0.00 | 0.00 | 0.00 | 0.00 | 0.00 | 0.00 | 0.00 | 0.00 | 0.00 |  | 0.00 | 0.00 | 0.00 | 0.00 | 0.00 | 0.00 |
|  | *Arthrobacter* |  | 2.52 | 1.18 | 0.44 | 0.76 | 1.57 | 3.90 | 1.49 | 3.52 | 1.50 | 2.58 |  | 0.39 | 3.52 | 1.76 | 2.05 | 0.32 | 0.00 |
|  | *Blastococcus* | *aggregatus* | 0.00 | 0.00 | 0.00 | 0.00 | 0.00 | 0.00 | 0.00 | 0.00 | 0.00 | 0.00 |  | 0.05 | 0.00 | 0.00 | 0.00 | 0.00 | 0.00 |
|  | *Blastococcus* | *jejuensis* | 0.00 | 0.00 | 0.00 | 0.00 | 0.00 | 0.00 | 0.00 | 0.00 | 0.00 | 0.00 |  | 0.00 | 0.00 | 0.00 | 0.00 | 0.00 | 0.00 |
|  | *Brachybacterium* |  | 0.00 | 0.00 | 0.00 | 0.00 | 0.00 | 0.00 | 0.00 | 0.00 | 0.00 | 0.00 |  | 0.21 | 0.00 | 0.00 | 0.00 | 0.00 | 0.00 |
|  | *Brachybacterium* | *conglomeratum* | 0.00 | 0.00 | 0.00 | 0.00 | 0.00 | 0.00 | 0.00 | 0.00 | 0.00 | 0.00 |  | 0.00 | 0.00 | 0.00 | 0.00 | 0.00 | 0.00 |
|  | *Brevibacterium* |  | 0.00 | 0.00 | 0.00 | 0.00 | 0.00 | 0.00 | 0.00 | 0.00 | 0.00 | 0.00 |  | 0.00 | 0.00 | 0.00 | 0.00 | 0.00 | 0.70 |
|  | *Brevibacterium* | *paucivorans* | 0.00 | 0.00 | 0.00 | 0.00 | 0.00 | 0.00 | 0.00 | 0.00 | 0.00 | 0.00 |  | 0.00 | 0.00 | 0.00 | 0.00 | 0.00 | 0.92 |
|  | *Catellatospora* |  | 0.00 | 0.00 | 0.00 | 0.00 | 0.00 | 0.00 | 0.00 | 0.00 | 0.00 | 0.00 |  | 0.00 | 0.00 | 0.00 | 0.00 | 0.03 | 0.00 |
|  | *Cellulomonas* |  | 0.00 | 0.00 | 0.05 | 0.00 | 0.00 | 0.00 | 0.21 | 0.17 | 0.00 | 0.38 |  | 0.00 | 0.50 | 0.11 | 0.00 | 0.00 | 0.00 |
|  | *Corynebacterium* |  | 0.00 | 0.00 | 0.00 | 0.00 | 0.00 | 0.00 | 0.00 | 0.00 | 0.00 | 0.00 |  | 0.53 | 0.00 | 0.00 | 0.00 | 0.00 | 8.55 |
|  | *Couchioplanes* |  | 0.00 | 0.00 | 0.00 | 0.00 | 0.00 | 0.00 | 0.00 | 0.00 | 0.00 | 0.15 |  | 0.00 | 0.00 | 0.00 | 0.00 | 0.00 | 0.00 |
|  | *Cryocola* |  | 0.00 | 0.00 | 0.00 | 0.00 | 0.00 | 0.00 | 0.00 | 0.00 | 0.00 | 0.07 |  | 0.00 | 0.00 | 0.00 | 0.00 | 0.00 | 0.00 |
|  | *Dactylosporangium* |  | 0.00 | 0.00 | 0.00 | 0.00 | 0.00 | 0.00 | 0.19 | 0.22 | 0.00 | 0.12 |  | 0.00 | 0.00 | 0.11 | 0.00 | 0.00 | 0.00 |
|  | *Dermacoccus* |  | 0.00 | 0.00 | 0.00 | 0.00 | 0.00 | 0.00 | 0.00 | 0.00 | 0.00 | 0.00 |  | 0.00 | 0.00 | 0.00 | 0.00 | 0.00 | 0.46 |
|  | *Dietzia* |  | 0.00 | 0.00 | 0.00 | 0.00 | 0.00 | 0.00 | 0.00 | 0.00 | 0.00 | 0.00 |  | 0.00 | 0.00 | 0.00 | 0.00 | 0.00 | 0.00 |
|  | *Friedmanniella* |  | 0.00 | 0.00 | 0.00 | 0.00 | 0.00 | 0.00 | 0.00 | 0.00 | 0.00 | 0.00 |  | 0.00 | 0.00 | 0.00 | 0.00 | 0.00 | 0.34 |
|  | *Frigoribacterium* |  | 0.00 | 0.00 | 0.00 | 0.00 | 0.00 | 0.00 | 0.00 | 0.00 | 0.00 | 0.00 |  | 0.00 | 0.00 | 0.00 | 0.00 | 0.00 | 0.00 |
|  | *Geodermatophilus* |  | 0.00 | 0.00 | 0.00 | 0.00 | 0.00 | 0.00 | 0.08 | 0.00 | 0.00 | 0.00 |  | 0.00 | 0.00 | 0.00 | 0.00 | 0.00 | 0.00 |
|  | *Gordonia* |  | 0.00 | 0.00 | 0.00 | 0.00 | 0.00 | 0.00 | 0.00 | 0.47 | 0.00 | 0.00 |  | 0.00 | 0.00 | 0.00 | 0.00 | 0.00 | 0.00 |
|  | *Kocuria* | *palustris* | 0.00 | 0.00 | 0.00 | 0.00 | 0.00 | 0.00 | 0.00 | 0.00 | 0.00 | 0.00 |  | 0.00 | 0.00 | 0.00 | 0.00 | 0.00 | 0.44 |
|  | *Kocuria* |  | 0.00 | 0.00 | 0.00 | 0.00 | 0.00 | 0.00 | 0.00 | 0.00 | 0.00 | 0.00 |  | 0.00 | 0.00 | 0.00 | 0.00 | 0.00 | 0.00 |
|  | *Kribbella* |  | 0.00 | 0.00 | 0.20 | 0.00 | 0.00 | 0.18 | 0.00 | 0.14 | 0.00 | 0.03 |  | 0.00 | 0.00 | 0.08 | 0.00 | 0.00 | 0.00 |
|  | *Kytococcus* |  | 0.00 | 0.00 | 0.00 | 0.00 | 0.00 | 0.00 | 0.00 | 0.00 | 0.00 | 0.00 |  | 0.00 | 0.00 | 0.00 | 0.00 | 0.00 | 0.00 |
|  | *Marmoricola* | *bigeumensis* | 0.00 | 0.00 | 0.00 | 0.00 | 0.00 | 0.00 | 0.00 | 0.00 | 0.00 | 0.00 |  | 0.00 | 0.00 | 0.00 | 0.00 | 0.00 | 0.00 |
|  | *Microbacterium* | *aurum* | 0.00 | 0.00 | 0.00 | 0.00 | 0.00 | 0.00 | 0.00 | 0.00 | 0.00 | 0.00 |  | 0.00 | 0.00 | 0.00 | 0.00 | 0.00 | 0.00 |
|  | *Microbacterium* | *chocolatum* | 0.00 | 0.00 | 0.00 | 0.00 | 0.00 | 0.00 | 0.00 | 0.00 | 0.00 | 0.00 |  | 0.09 | 0.00 | 0.00 | 0.00 | 0.00 | 0.00 |
|  | *Microbacterium* | *maritypicum* | 0.00 | 0.00 | 0.00 | 0.00 | 0.00 | 0.00 | 0.00 | 0.00 | 0.00 | 0.00 |  | 0.09 | 0.00 | 0.00 | 0.00 | 0.00 | 0.00 |
|  | *Microbacterium* |  | 0.00 | 0.00 | 0.00 | 0.00 | 0.00 | 0.00 | 0.00 | 0.00 | 0.00 | 0.00 |  | 0.00 | 0.00 | 0.00 | 0.00 | 0.00 | 0.41 |
|  | *Micrococcus* |  | 0.00 | 0.00 | 0.00 | 0.00 | 0.00 | 0.00 | 0.00 | 0.00 | 0.00 | 0.00 |  | 0.24 | 0.00 | 0.21 | 0.00 | 0.00 | 10.50 |
|  | *Microlunatus* |  | 0.00 | 0.18 | 0.18 | 0.00 | 0.00 | 0.00 | 0.00 | 0.00 | 0.00 | 0.00 |  | 0.00 | 0.00 | 0.00 | 0.00 | 0.00 | 0.00 |
|  | *Micromonospora* | *coxensis* | 0.00 | 0.00 | 0.00 | 0.00 | 0.00 | 0.00 | 0.00 | 0.00 | 0.00 | 0.00 |  | 0.00 | 0.00 | 0.00 | 0.00 | 0.00 | 0.00 |
|  | *Micromonospora* |  | 0.00 | 0.00 | 0.00 | 0.00 | 0.00 | 0.00 | 0.00 | 0.00 | 0.00 | 0.21 |  | 0.00 | 0.00 | 0.00 | 0.00 | 0.00 | 0.00 |
|  | *Modestobacter* |  | 0.00 | 0.00 | 0.00 | 0.00 | 0.00 | 0.00 | 0.00 | 0.19 | 0.00 | 0.00 |  | 0.00 | 0.00 | 0.00 | 0.00 | 0.04 | 0.00 |
|  | *Mycobacterium* |  | 0.00 | 0.00 | 0.44 | 1.26 | 0.37 | 0.00 | 1.00 | 1.09 | 0.36 | 0.88 |  | 0.29 | 0.81 | 0.59 | 0.00 | 0.37 | 0.48 |
|  | *Mycobacterium* | *gordonae* | 0.00 | 0.00 | 0.00 | 0.00 | 0.00 | 0.00 | 0.00 | 0.00 | 0.09 | 0.00 |  | 0.00 | 0.00 | 0.00 | 0.00 | 0.00 | 0.00 |
|  | *Mycobacterium* | *vaccae* | 0.00 | 0.00 | 0.00 | 0.00 | 0.00 | 0.00 | 0.00 | 0.33 | 0.00 | 0.27 |  | 0.16 | 0.00 | 0.19 | 0.00 | 0.12 | 0.00 |
|  | *Nocardia* |  | 0.00 | 0.00 | 0.00 | 0.00 | 0.00 | 0.00 | 0.00 | 0.00 | 0.00 | 0.00 |  | 0.00 | 0.00 | 0.00 | 0.00 | 0.00 | 0.00 |
|  | *Nocardioides* |  | 0.00 | 0.00 | 0.18 | 0.09 | 0.15 | 0.00 | 0.73 | 0.22 | 0.76 | 1.02 |  | 0.00 | 0.45 | 0.00 | 0.00 | 0.18 | 0.00 |
|  | *Nocardioides* | *fulvus* | 0.00 | 0.00 | 0.00 | 0.00 | 0.00 | 0.00 | 0.00 | 0.21 | 0.00 | 0.15 |  | 0.00 | 0.00 | 0.00 | 0.00 | 0.00 | 0.00 |
|  | *Nocardioides* | *pyridinolyticus* | 0.00 | 0.00 | 0.00 | 0.00 | 0.00 | 0.00 | 0.00 | 0.00 | 0.00 | 0.00 |  | 0.23 | 0.00 | 0.00 | 0.00 | 0.00 | 0.00 |
|  | *Nocardioides* | *terrigena* | 0.00 | 0.00 | 0.00 | 0.00 | 0.00 | 0.00 | 0.00 | 0.17 | 0.00 | 0.21 |  | 0.00 | 0.00 | 0.26 | 0.00 | 0.04 | 0.00 |
|  | *Nocardioides* | *koreensis* | 0.00 | 0.00 | 0.00 | 0.00 | 0.15 | 0.00 | 0.00 | 0.00 | 0.00 | 0.00 |  | 0.00 | 0.00 | 0.11 | 0.00 | 0.00 | 0.00 |
|  | *Nocardioides* | *halotolerans* | 0.00 | 0.00 | 0.00 | 0.00 | 0.00 | 0.00 | 0.00 | 0.00 | 0.00 | 0.27 |  | 0.00 | 0.00 | 0.00 | 0.00 | 0.03 | 0.00 |
|  | *Nocardioides* | *maritimus* | 0.00 | 0.00 | 0.00 | 0.07 | 0.00 | 0.00 | 0.00 | 0.00 | 0.00 | 0.00 |  | 0.00 | 0.00 | 0.00 | 0.00 | 0.00 | 0.00 |
|  | *Nocardioides* | *tritolerans* | 0.00 | 0.00 | 0.00 | 0.00 | 0.00 | 0.00 | 0.00 | 0.00 | 0.00 | 0.00 |  | 0.00 | 0.00 | 0.00 | 0.00 | 0.00 | 0.00 |
|  | *Nocardioides* | *oleivorans* | 0.00 | 0.00 | 0.00 | 0.00 | 0.00 | 0.00 | 0.00 | 0.00 | 0.00 | 0.00 |  | 0.00 | 0.00 | 0.00 | 0.00 | 0.00 | 0.00 |
|  | *Oryzihumus* | *leptocrescens* | 0.00 | 0.00 | 0.09 | 0.00 | 0.08 | 0.00 | 0.45 | 0.14 | 0.34 | 0.21 |  | 0.00 | 0.00 | 0.09 | 0.00 | 0.00 | 0.00 |
|  | *Phycicoccus* |  | 0.00 | 0.00 | 0.24 | 1.19 | 0.00 | 0.00 | 1.32 | 0.61 | 0.00 | 0.94 |  | 0.00 | 0.00 | 0.21 | 0.00 | 0.65 | 0.00 |
|  | *Pilimelia* |  | 0.00 | 0.00 | 0.00 | 0.16 | 0.00 | 0.00 | 0.00 | 0.00 | 0.18 | 0.03 |  | 0.17 | 0.00 | 0.00 | 0.00 | 0.00 | 0.00 |
|  | *Propionibacterium* |  | 0.00 | 0.00 | 0.00 | 0.00 | 0.00 | 0.00 | 0.00 | 0.00 | 0.00 | 0.02 |  | 2.04 | 0.00 | 0.11 | 6.14 | 0.46 | 8.20 |
|  | *Propionibacterium* | *acnes* | 0.00 | 0.00 | 0.00 | 0.00 | 0.00 | 0.00 | 0.00 | 0.00 | 0.00 | 0.02 |  | 1.95 | 0.00 | 0.11 | 6.14 | 0.46 | 8.20 |
|  | *Propionibacterium* | *granulosum* | 0.00 | 0.00 | 0.00 | 0.00 | 0.00 | 0.00 | 0.00 | 0.00 | 0.00 | 0.00 |  | 0.10 | 0.00 | 0.00 | 0.00 | 0.00 | 0.00 |
|  | *Propionicimonas* |  | 0.00 | 0.00 | 0.05 | 0.00 | 0.00 | 0.00 | 0.00 | 0.00 | 0.00 | 0.14 |  | 0.00 | 0.00 | 0.00 | 0.00 | 0.00 | 0.00 |
|  | *Pseudonocardia* |  | 0.00 | 0.19 | 0.16 | 0.21 | 0.00 | 0.00 | 0.66 | 0.00 | 0.00 | 0.41 |  | 0.00 | 0.00 | 0.18 | 0.00 | 0.14 | 0.00 |
|  | *Rhodococcus* | *ruber* | 0.27 | 0.00 | 0.00 | 0.00 | 0.00 | 0.00 | 0.00 | 0.00 | 0.00 | 0.00 |  | 0.00 | 0.00 | 0.00 | 0.00 | 0.00 | 0.00 |
|  | *Rhodococcus* | *fascians* | 0.00 | 0.00 | 0.00 | 0.00 | 0.00 | 0.00 | 0.00 | 0.00 | 0.00 | 0.00 |  | 0.00 | 0.00 | 0.00 | 0.00 | 0.00 | 0.00 |
|  | *Rhodococcus* |  | 0.00 | 0.00 | 0.00 | 0.00 | 0.00 | 0.00 | 0.13 | 0.13 | 0.00 | 0.11 |  | 0.00 | 0.00 | 0.00 | 0.00 | 0.00 | 1.12 |
|  | *Rothia* | *aeria* | 0.00 | 0.00 | 0.04 | 0.00 | 0.00 | 0.00 | 0.00 | 0.00 | 0.00 | 0.00 |  | 1.67 | 0.00 | 0.00 | 0.00 | 0.00 | 0.00 |
|  | *Rothia* | *mucilaginosa* | 0.00 | 0.00 | 0.00 | 0.00 | 0.00 | 0.00 | 0.00 | 0.00 | 0.00 | 0.00 |  | 1.59 | 0.00 | 0.00 | 0.00 | 0.00 | 0.33 |
|  | *Saccharopolyspora* |  | 0.00 | 0.00 | 0.00 | 0.00 | 0.00 | 0.00 | 0.00 | 0.00 | 0.00 | 0.00 |  | 0.00 | 0.00 | 0.00 | 0.00 | 0.00 | 0.00 |
|  | *Saccharothrix* |  | 0.00 | 0.00 | 0.00 | 0.00 | 0.00 | 0.00 | 0.00 | 0.10 | 0.00 | 0.00 |  | 0.00 | 0.00 | 0.00 | 0.00 | 0.00 | 0.00 |
|  | *Salinibacterium* |  | 0.00 | 0.00 | 0.00 | 0.00 | 0.00 | 0.00 | 0.00 | 0.00 | 0.00 | 0.17 |  | 0.00 | 0.00 | 0.00 | 0.00 | 0.00 | 0.00 |
|  | *Serinicoccus* |  | 0.00 | 0.00 | 0.00 | 0.00 | 0.00 | 0.00 | 0.00 | 0.00 | 0.00 | 0.00 |  | 0.00 | 0.00 | 0.00 | 0.00 | 0.00 | 1.20 |
|  | *Solwaraspora* |  | 0.00 | 0.00 | 0.00 | 0.25 | 0.00 | 0.00 | 0.00 | 0.00 | 0.00 | 0.00 |  | 0.00 | 0.00 | 0.00 | 0.00 | 0.00 | 0.00 |
|  | *Sphaerisporangium* |  | 0.00 | 0.00 | 0.00 | 0.00 | 0.00 | 0.00 | 0.00 | 0.15 | 0.00 | 0.00 |  | 0.00 | 0.00 | 0.07 | 0.00 | 0.00 | 0.00 |
|  | *Streptomyces* |  | 0.00 | 0.00 | 0.00 | 0.00 | 0.00 | 0.00 | 0.22 | 0.68 | 0.15 | 0.21 |  | 0.13 | 0.45 | 0.00 | 0.00 | 0.00 | 0.00 |
|  | *Streptomyces* | *lanatus* | 0.00 | 0.00 | 0.14 | 0.00 | 0.00 | 0.00 | 0.37 | 0.21 | 0.14 | 0.29 |  | 0.21 | 0.00 | 0.09 | 0.00 | 0.00 | 0.00 |
|  | *Streptomyces* | *mirabilis* | 0.00 | 0.00 | 0.00 | 0.39 | 0.00 | 0.00 | 0.00 | 0.16 | 0.00 | 0.00 |  | 0.00 | 0.00 | 0.56 | 0.00 | 0.00 | 0.00 |
|  | *Streptomyces* | *radiopugnans* | 0.00 | 0.00 | 0.27 | 0.00 | 0.00 | 0.00 | 0.00 | 0.00 | 0.00 | 0.00 |  | 0.00 | 0.00 | 0.00 | 0.00 | 0.00 | 0.00 |
|  | *Streptomyces* | *reticuliscabiei* | 0.00 | 0.00 | 0.00 | 0.15 | 0.00 | 0.00 | 0.00 | 0.17 | 0.00 | 0.25 |  | 0.00 | 0.00 | 0.00 | 0.00 | 0.00 | 0.00 |
|  | *Streptosporangium* |  | 0.00 | 0.00 | 0.00 | 0.00 | 0.00 | 0.00 | 0.00 | 0.11 | 0.00 | 0.00 |  | 0.00 | 0.00 | 0.00 | 0.00 | 0.00 | 0.00 |
|  | *Terracoccus* |  | 0.00 | 0.00 | 0.21 | 0.00 | 0.44 | 1.80 | 1.02 | 0.52 | 0.84 | 1.18 |  | 0.18 | 0.00 | 0.36 | 0.00 | 0.15 | 0.00 |
|  | *Tetrasphaera* |  | 0.00 | 0.00 | 0.00 | 0.00 | 0.00 | 0.00 | 0.00 | 0.00 | 0.00 | 0.00 |  | 0.00 | 0.00 | 0.00 | 0.00 | 0.00 | 0.00 |
|  | *Virgisporangium* |  | 0.00 | 0.00 | 0.00 | 0.00 | 0.00 | 0.00 | 0.00 | 0.00 | 0.00 | 0.00 |  | 0.00 | 0.00 | 0.00 | 0.00 | 0.00 | 0.00 |
|  | *Virgisporangium* | *ochraceum* | 0.00 | 0.00 | 0.20 | 0.00 | 0.00 | 0.00 | 0.00 | 0.15 | 0.00 | 0.00 |  | 0.00 | 0.00 | 0.00 | 0.00 | 0.06 | 0.00 |
| *AK31* |  |  | 14.94 | 1.97 | 0.78 | 0.33 | 0.40 | 0.64 | 0.14 | 2.09 | 0.11 | 0.15 |  | 0.16 | 3.93 | 1.79 | 0.00 | 0.37 | 0.00 |
| *Anaerolineales* |  |  | 1.12 | 0.61 | 2.20 | 0.57 | 1.38 | 0.00 | 1.05 | 0.26 | 1.23 | 1.79 |  | 0.15 | 1.62 | 1.51 | 2.05 | 1.55 | 0.00 |
|  | *Anaerolinea* |  | 1.12 | 0.61 | 2.20 | 0.57 | 1.38 | 0.00 | 1.05 | 0.26 | 1.23 | 1.79 |  | 0.15 | 1.62 | 1.46 | 2.05 | 1.55 | 0.00 |
|  | *Longilinea* |  | 0.00 | 0.00 | 0.00 | 0.00 | 0.00 | 0.00 | 0.00 | 0.00 | 0.00 | 0.00 |  | 0.00 | 0.00 | 0.00 | 0.00 | 0.00 | 0.00 |
|  | *SHD-14* |  | 0.00 | 0.00 | 0.00 | 0.00 | 0.00 | 0.00 | 0.00 | 0.00 | 0.00 | 0.00 |  | 0.00 | 0.00 | 0.00 | 0.00 | 0.00 | 0.00 |
|  | *SHD-231* |  | 0.00 | 0.00 | 0.00 | 0.00 | 0.00 | 0.00 | 0.00 | 0.00 | 0.00 | 0.00 |  | 0.00 | 0.00 | 0.05 | 0.00 | 0.00 | 0.00 |
| *Bacillales* |  |  | 1.58 | 3.42 | 1.35 | 2.55 | 1.26 | 6.01 | 3.05 | 9.09 | 1.46 | 4.34 |  | 1.68 | 1.22 | 3.51 | 0.58 | 0.12 | 3.97 |
|  | *Alicyclobacillus* |  | 0.17 | 0.58 | 0.09 | 0.42 | 0.37 | 0.77 | 0.22 | 1.32 | 0.15 | 0.81 |  | 0.12 | 0.00 | 0.65 | 0.00 | 0.00 | 0.00 |
|  | *Ammoniphilus* |  | 0.00 | 0.00 | 0.00 | 0.00 | 0.00 | 0.75 | 0.24 | 0.00 | 0.00 | 0.07 |  | 0.00 | 0.00 | 0.08 | 0.00 | 0.00 | 0.00 |
|  | *Bacillus* |  | 0.58 | 0.53 | 0.39 | 0.23 | 0.24 | 1.06 | 1.34 | 3.07 | 0.24 | 1.86 |  | 0.74 | 0.36 | 0.61 | 0.00 | 0.10 | 2.05 |
|  | *Bacillus* | *muralis* | 0.40 | 0.00 | 0.00 | 0.34 | 0.00 | 0.00 | 0.00 | 1.18 | 0.24 | 0.00 |  | 0.00 | 0.00 | 0.39 | 0.58 | 0.00 | 0.00 |
|  | *Bacillus* | *flexus* | 0.37 | 0.42 | 0.20 | 0.55 | 0.24 | 0.40 | 0.54 | 0.61 | 0.19 | 0.22 |  | 0.00 | 0.86 | 0.52 | 0.00 | 0.00 | 0.00 |
|  | *Bacillus* | *fumarioli* | 0.00 | 0.43 | 0.28 | 0.33 | 0.20 | 1.94 | 0.48 | 1.48 | 0.33 | 0.70 |  | 0.00 | 0.00 | 0.62 | 0.00 | 0.00 | 0.00 |
|  | *Bacillus* | *psychrodurans* | 0.00 | 0.40 | 0.00 | 0.00 | 0.00 | 0.00 | 0.00 | 0.00 | 0.00 | 0.00 |  | 0.00 | 0.00 | 0.00 | 0.00 | 0.00 | 0.00 |
|  | *Bacillus* | *longiquaesitum* | 0.00 | 0.00 | 0.15 | 0.00 | 0.20 | 1.10 | 0.23 | 0.18 | 0.31 | 0.00 |  | 0.00 | 0.00 | 0.20 | 0.00 | 0.00 | 0.00 |
|  | *Bacillus* | *mannanilyticus* | 0.00 | 0.00 | 0.00 | 0.00 | 0.00 | 0.00 | 0.00 | 0.00 | 0.00 | 0.00 |  | 0.00 | 0.00 | 0.00 | 0.00 | 0.00 | 0.00 |
|  | *Bacillus* | *marisflavi* | 0.00 | 0.00 | 0.00 | 0.00 | 0.00 | 0.00 | 0.00 | 0.00 | 0.00 | 0.00 |  | 0.00 | 0.00 | 0.00 | 0.00 | 0.00 | 0.00 |
|  | *Bacillus* | *selenatarsenatis* | 0.00 | 0.00 | 0.09 | 0.19 | 0.00 | 0.00 | 0.00 | 0.00 | 0.00 | 0.19 |  | 0.00 | 0.00 | 0.20 | 0.00 | 0.00 | 0.00 |
|  | *Bacillus* | *humi* | 0.00 | 0.00 | 0.00 | 0.00 | 0.00 | 0.00 | 0.00 | 0.00 | 0.00 | 0.00 |  | 0.00 | 0.00 | 0.00 | 0.00 | 0.00 | 0.00 |
|  | *Bacillus* |  | 0.00 | 0.00 | 0.00 | 0.00 | 0.00 | 0.00 | 0.00 | 0.00 | 0.00 | 0.00 |  | 0.00 | 0.00 | 0.00 | 0.00 | 0.00 | 0.00 |
|  | *Brevibacillus* | *thermoruber* | 0.00 | 0.00 | 0.00 | 0.00 | 0.00 | 0.00 | 0.00 | 0.10 | 0.00 | 0.00 |  | 0.00 | 0.00 | 0.00 | 0.00 | 0.00 | 0.00 |
|  | *Geobacillus* |  | 0.00 | 0.00 | 0.00 | 0.00 | 0.00 | 0.00 | 0.00 | 0.00 | 0.00 | 0.00 |  | 0.00 | 0.00 | 0.00 | 0.00 | 0.00 | 0.00 |
|  | *Gracilibacillus* |  | 0.00 | 0.00 | 0.00 | 0.00 | 0.00 | 0.00 | 0.00 | 0.06 | 0.00 | 0.00 |  | 0.00 | 0.00 | 0.00 | 0.00 | 0.00 | 0.00 |
|  | *Jeotgalicoccus* |  | 0.00 | 0.00 | 0.00 | 0.00 | 0.00 | 0.00 | 0.00 | 0.00 | 0.00 | 0.00 |  | 0.00 | 0.00 | 0.00 | 0.00 | 0.00 | 0.00 |
|  | *Jeotgalicoccus* | *psychrophilus* | 0.00 | 0.00 | 0.00 | 0.00 | 0.00 | 0.00 | 0.00 | 0.00 | 0.00 | 0.00 |  | 0.00 | 0.00 | 0.00 | 0.00 | 0.00 | 0.00 |
|  | *Lysinibacillus* | *boronitolerans* | 0.00 | 0.00 | 0.00 | 0.00 | 0.00 | 0.00 | 0.00 | 0.00 | 0.00 | 0.00 |  | 0.00 | 0.00 | 0.00 | 0.00 | 0.00 | 0.00 |
|  | *Lysinibacillus* | *massiliensis* | 0.00 | 0.00 | 0.00 | 0.00 | 0.00 | 0.00 | 0.00 | 0.12 | 0.00 | 0.00 |  | 0.00 | 0.00 | 0.00 | 0.00 | 0.00 | 0.00 |
|  | *Marinibacillus* |  | 0.00 | 0.00 | 0.00 | 0.00 | 0.00 | 0.00 | 0.00 | 0.00 | 0.00 | 0.00 |  | 0.00 | 0.00 | 0.09 | 0.00 | 0.00 | 0.00 |
|  | *Oceanobacillus* | *caeni* | 0.00 | 0.00 | 0.00 | 0.00 | 0.00 | 0.00 | 0.00 | 0.23 | 0.00 | 0.00 |  | 0.00 | 0.00 | 0.00 | 0.00 | 0.00 | 0.00 |
|  | *Paenibacillus* |  | 0.00 | 0.32 | 0.00 | 0.11 | 0.00 | 0.00 | 0.00 | 0.44 | 0.00 | 0.09 |  | 0.00 | 0.00 | 0.11 | 0.00 | 0.00 | 0.00 |
|  | *Paenibacillus* | *chondroitinus* | 0.00 | 0.00 | 0.00 | 0.00 | 0.00 | 0.00 | 0.00 | 0.00 | 0.00 | 0.00 |  | 0.24 | 0.00 | 0.00 | 0.00 | 0.00 | 0.00 |
|  | *Paenibacillus* | *curdlanolyticus* | 0.00 | 0.00 | 0.00 | 0.00 | 0.00 | 0.00 | 0.00 | 0.00 | 0.00 | 0.00 |  | 0.00 | 0.00 | 0.05 | 0.00 | 0.00 | 0.00 |
|  | *Paenibacillus* | *amylolyticus* | 0.00 | 0.00 | 0.00 | 0.00 | 0.00 | 0.00 | 0.00 | 0.00 | 0.00 | 0.09 |  | 0.00 | 0.00 | 0.00 | 0.00 | 0.00 | 0.00 |
|  | *Paenisporosarcina* |  | 0.00 | 0.00 | 0.10 | 0.39 | 0.00 | 0.00 | 0.00 | 0.02 | 0.00 | 0.08 |  | 0.00 | 0.00 | 0.00 | 0.00 | 0.00 | 0.00 |
|  | *Paucisalibacillus* | *globulus* | 0.00 | 0.00 | 0.00 | 0.00 | 0.00 | 0.00 | 0.00 | 0.00 | 0.00 | 0.00 |  | 0.00 | 0.00 | 0.00 | 0.00 | 0.00 | 0.00 |
|  | *Planifilum* |  | 0.00 | 0.00 | 0.00 | 0.00 | 0.00 | 0.00 | 0.00 | 0.00 | 0.00 | 0.18 |  | 0.00 | 0.00 | 0.00 | 0.00 | 0.00 | 0.00 |
|  | *Rummeliibacillus* |  | 0.00 | 0.00 | 0.00 | 0.00 | 0.00 | 0.00 | 0.00 | 0.27 | 0.00 | 0.00 |  | 0.00 | 0.00 | 0.00 | 0.00 | 0.00 | 0.00 |
|  | *Shimazuella* |  | 0.06 | 0.00 | 0.06 | 0.00 | 0.00 | 0.00 | 0.00 | 0.00 | 0.00 | 0.00 |  | 0.00 | 0.00 | 0.00 | 0.00 | 0.00 | 0.00 |
|  | *Solibacillus* |  | 0.00 | 0.00 | 0.00 | 0.00 | 0.00 | 0.00 | 0.00 | 0.00 | 0.00 | 0.05 |  | 0.00 | 0.00 | 0.00 | 0.00 | 0.00 | 0.00 |
|  | *Sporosarcina* |  | 0.00 | 0.73 | 0.00 | 0.00 | 0.00 | 0.00 | 0.00 | 0.00 | 0.00 | 0.00 |  | 0.00 | 0.00 | 0.00 | 0.00 | 0.00 | 0.00 |
|  | *Staphylococcus* |  | 0.00 | 0.00 | 0.00 | 0.00 | 0.00 | 0.00 | 0.00 | 0.00 | 0.00 | 0.00 |  | 0.44 | 0.00 | 0.00 | 0.00 | 0.01 | 1.92 |
|  | *Staphylococcus* | *equorum* | 0.00 | 0.00 | 0.00 | 0.00 | 0.00 | 0.00 | 0.00 | 0.00 | 0.00 | 0.00 |  | 0.14 | 0.00 | 0.00 | 0.00 | 0.00 | 0.00 |
|  | *Thermoactinomyces* |  | 0.00 | 0.00 | 0.00 | 0.00 | 0.00 | 0.00 | 0.00 | 0.00 | 0.00 | 0.00 |  | 0.00 | 0.00 | 0.00 | 0.00 | 0.00 | 0.00 |
|  | *Ureibacillus* |  | 0.00 | 0.00 | 0.00 | 0.00 | 0.00 | 0.00 | 0.00 | 0.00 | 0.00 | 0.00 |  | 0.00 | 0.00 | 0.00 | 0.00 | 0.00 | 0.00 |
| *Bacteroidales* |  |  | 0.81 | 1.53 | 3.52 | 0.20 | 2.10 | 0.11 | 0.21 | 0.00 | 1.04 | 0.51 |  | 0.00 | 0.00 | 0.47 | 3.51 | 0.34 | 0.00 |
|  | *Bacteroides* |  | 0.00 | 0.00 | 0.06 | 0.00 | 0.00 | 0.00 | 0.00 | 0.00 | 0.00 | 0.06 |  | 0.00 | 0.00 | 0.06 | 0.00 | 0.05 | 0.00 |
|  | *Bacteroides* | *fragilis* | 0.00 | 0.00 | 0.00 | 0.00 | 0.00 | 0.00 | 0.00 | 0.00 | 0.00 | 0.00 |  | 0.00 | 0.00 | 0.00 | 0.00 | 0.00 | 0.00 |
|  | *Blvii28* |  | 0.00 | 0.00 | 0.00 | 0.00 | 0.00 | 0.00 | 0.00 | 0.00 | 0.00 | 0.00 |  | 0.00 | 0.00 | 0.00 | 0.00 | 0.00 | 0.00 |
|  | *Macellibacteroides* | *fermentans* | 0.00 | 0.00 | 0.00 | 0.00 | 0.00 | 0.00 | 0.00 | 0.00 | 0.00 | 0.00 |  | 0.00 | 0.00 | 0.00 | 0.00 | 0.00 | 0.00 |
|  | *Paludibacter* |  | 0.00 | 0.00 | 0.00 | 0.00 | 0.00 | 0.00 | 0.00 | 0.00 | 0.00 | 0.07 |  | 0.33 | 0.00 | 0.00 | 0.00 | 0.00 | 0.00 |
|  | *Porphyromonas* |  | 0.00 | 0.00 | 0.00 | 0.00 | 0.00 | 0.00 | 0.00 | 0.00 | 0.00 | 0.00 |  | 7.87 | 0.00 | 0.00 | 2.34 | 0.09 | 0.00 |
|  | *Porphyromonas* | *endodontalis* | 0.00 | 0.00 | 0.00 | 0.00 | 0.00 | 0.00 | 0.00 | 0.00 | 0.00 | 0.00 |  | 5.91 | 0.00 | 0.00 | 0.00 | 0.00 | 0.00 |
|  | *Prevotella* |  | 0.00 | 0.00 | 0.09 | 0.00 | 0.00 | 0.00 | 0.00 | 0.00 | 0.00 | 0.00 |  | 6.92 | 0.00 | 0.03 | 0.00 | 0.01 | 0.00 |
|  | *Prevotella* | *tannerae* | 0.00 | 0.00 | 0.02 | 0.00 | 0.00 | 0.00 | 0.00 | 0.00 | 0.00 | 0.00 |  | 0.35 | 0.00 | 0.00 | 0.00 | 0.00 | 0.00 |
|  | *Prevotella* | *pallens* | 0.00 | 0.00 | 0.00 | 0.00 | 0.00 | 0.00 | 0.00 | 0.00 | 0.00 | 0.00 |  | 0.26 | 0.00 | 0.00 | 0.00 | 0.00 | 0.00 |
|  | *Prevotella* | *melaninogenica* | 0.00 | 0.00 | 0.00 | 0.00 | 0.00 | 0.00 | 0.00 | 0.00 | 0.00 | 0.00 |  | 0.00 | 0.00 | 0.00 | 0.00 | 0.00 | 0.29 |
|  | *Prevotella* | *intermedia* | 0.00 | 0.00 | 0.00 | 0.00 | 0.00 | 0.00 | 0.00 | 0.00 | 0.00 | 0.00 |  | 0.35 | 0.00 | 0.00 | 0.00 | 0.00 | 0.00 |
|  | *Tannerella* |  | 0.00 | 0.00 | 0.00 | 0.00 | 0.00 | 0.00 | 0.00 | 0.00 | 0.00 | 0.00 |  | 0.39 | 0.00 | 0.00 | 0.00 | 0.00 | 0.00 |
| *Burkholderiales* |  |  | 1.54 | 0.11 | 0.64 | 0.65 | 0.53 | 0.00 | 0.00 | 0.37 | 0.22 | 0.29 |  | 0.05 | 2.44 | 0.68 | 0.00 | 0.82 | 0.00 |
|  | *Achromobacter* |  | 0.00 | 0.00 | 0.00 | 0.00 | 0.03 | 0.00 | 0.00 | 0.00 | 0.00 | 0.00 |  | 0.00 | 0.00 | 0.00 | 0.00 | 0.00 | 0.00 |
|  | *Acidovorax* |  | 0.00 | 0.00 | 0.00 | 0.00 | 0.00 | 0.00 | 0.00 | 0.00 | 0.00 | 0.00 |  | 0.00 | 0.00 | 0.00 | 0.00 | 0.00 | 0.00 |
|  | *Aquabacterium* |  | 0.00 | 0.00 | 0.00 | 0.00 | 0.00 | 0.00 | 0.00 | 0.00 | 0.00 | 0.00 |  | 0.00 | 0.00 | 0.00 | 0.00 | 0.00 | 0.00 |
|  | *Burkholderia* |  | 0.00 | 0.00 | 0.00 | 0.00 | 0.00 | 0.00 | 0.00 | 0.00 | 0.00 | 0.00 |  | 0.00 | 0.00 | 0.00 | 0.00 | 0.00 | 0.00 |
|  | *Cupriavidus* |  | 0.00 | 0.00 | 0.00 | 0.00 | 0.00 | 0.00 | 0.00 | 0.07 | 0.00 | 0.00 |  | 0.00 | 0.00 | 0.00 | 0.00 | 0.00 | 0.00 |
|  | *Delftia* |  | 0.00 | 0.00 | 0.00 | 0.00 | 0.00 | 0.00 | 0.00 | 0.00 | 0.00 | 0.00 |  | 0.24 | 0.00 | 0.00 | 0.00 | 0.00 | 0.00 |
|  | *Duganella* | *nigrescens* | 0.00 | 0.00 | 0.00 | 0.00 | 0.00 | 0.00 | 0.00 | 0.00 | 0.00 | 0.00 |  | 0.00 | 0.00 | 0.00 | 0.00 | 0.00 | 0.00 |
|  | *Herminiimonas* |  | 0.00 | 0.00 | 0.00 | 0.00 | 0.00 | 0.00 | 0.00 | 0.00 | 0.00 | 0.00 |  | 0.00 | 0.00 | 0.00 | 0.00 | 0.00 | 0.00 |
|  | *Hydrogenophaga* |  | 0.00 | 0.00 | 0.00 | 0.00 | 0.00 | 0.00 | 0.00 | 0.00 | 0.00 | 0.00 |  | 0.00 | 0.00 | 0.00 | 0.00 | 0.00 | 0.00 |
|  | *Hylemonella* |  | 0.00 | 0.00 | 0.00 | 0.00 | 0.00 | 0.00 | 0.00 | 0.00 | 0.27 | 0.00 |  | 0.00 | 0.00 | 0.00 | 0.00 | 0.00 | 0.00 |
|  | *Janthinobacterium* |  | 0.00 | 0.00 | 0.00 | 0.00 | 0.37 | 1.03 | 0.00 | 0.26 | 0.00 | 0.00 |  | 0.00 | 0.00 | 0.00 | 0.00 | 0.00 | 0.00 |
|  | *Lautropia* |  | 0.00 | 0.00 | 0.00 | 0.00 | 0.00 | 0.00 | 0.00 | 0.00 | 0.00 | 0.00 |  | 0.56 | 0.00 | 0.00 | 0.00 | 0.00 | 0.00 |
|  | *Leptothrix* |  | 0.00 | 0.00 | 0.00 | 0.40 | 0.00 | 0.00 | 0.00 | 0.00 | 0.00 | 0.00 |  | 0.00 | 0.00 | 0.00 | 0.00 | 0.00 | 0.00 |
|  | *Massilia* |  | 0.00 | 0.00 | 0.00 | 0.00 | 0.00 | 0.00 | 0.00 | 0.00 | 0.00 | 0.23 |  | 0.00 | 0.00 | 0.00 | 0.00 | 0.08 | 0.00 |
|  | *Massilia* | *albidiflava* | 0.00 | 0.00 | 0.00 | 0.00 | 0.00 | 0.00 | 0.00 | 0.00 | 0.00 | 0.17 |  | 0.00 | 0.00 | 0.00 | 0.00 | 0.00 | 0.00 |
|  | *Massilia* | *niastensis* | 0.00 | 0.00 | 0.00 | 0.00 | 0.00 | 0.00 | 0.00 | 0.00 | 0.00 | 0.17 |  | 0.00 | 0.00 | 0.00 | 0.00 | 0.00 | 0.00 |
|  | *Methylibium* |  | 0.00 | 0.00 | 0.36 | 0.00 | 0.00 | 0.00 | 0.25 | 0.13 | 0.00 | 0.48 |  | 0.18 | 0.00 | 0.41 | 0.00 | 0.26 | 0.00 |
|  | *Ralstonia* |  | 0.00 | 0.00 | 0.00 | 0.00 | 0.00 | 0.00 | 0.00 | 0.00 | 0.00 | 0.00 |  | 0.00 | 0.00 | 0.00 | 0.00 | 0.00 | 0.00 |
|  | *Ramlibacter* |  | 0.00 | 0.00 | 1.21 | 0.13 | 0.11 | 0.59 | 0.00 | 0.45 | 0.00 | 0.23 |  | 0.00 | 1.35 | 0.00 | 0.00 | 0.22 | 0.00 |
|  | *Rubrivivax* |  | 0.00 | 0.14 | 0.00 | 0.00 | 0.00 | 0.00 | 0.00 | 0.00 | 0.00 | 0.00 |  | 0.00 | 0.00 | 0.00 | 0.00 | 0.00 | 0.00 |
|  | *Rubrivivax* | *gelatinosus* | 0.00 | 0.00 | 0.09 | 0.00 | 0.00 | 0.00 | 0.00 | 0.00 | 0.00 | 0.00 |  | 0.00 | 0.00 | 0.00 | 0.00 | 0.00 | 0.00 |
|  | *Variovorax* | *paradoxus* | 0.00 | 0.00 | 0.00 | 0.00 | 0.00 | 0.00 | 0.00 | 0.00 | 0.00 | 0.00 |  | 0.00 | 0.00 | 0.00 | 0.00 | 0.00 | 0.00 |
| *Clostridiales* |  |  | 0.38 | 1.15 | 0.27 | 0.15 | 0.19 | 0.07 | 0.67 | 0.47 | 0.66 | 0.46 |  | 1.28 | 1.26 | 1.13 | 0.00 | 0.24 | 1.24 |
|  | *Acidaminobacter* |  | 0.00 | 0.00 | 0.00 | 0.00 | 0.00 | 0.00 | 0.00 | 0.00 | 0.00 | 0.00 |  | 0.00 | 0.00 | 0.00 | 0.00 | 0.00 | 0.00 |
|  | *Anaerococcus* |  | 0.00 | 0.00 | 0.00 | 0.00 | 0.00 | 0.00 | 0.00 | 0.00 | 0.00 | 0.00 |  | 0.00 | 0.00 | 0.00 | 0.00 | 0.04 | 0.00 |
|  | *Anaerovorax* |  | 0.00 | 0.00 | 0.00 | 0.00 | 0.00 | 0.00 | 0.00 | 0.00 | 0.00 | 0.00 |  | 0.04 | 0.18 | 0.00 | 0.00 | 0.00 | 0.00 |
|  | *BSV43* |  | 0.08 | 0.21 | 0.16 | 0.00 | 0.14 | 0.00 | 0.00 | 0.00 | 0.17 | 0.06 |  | 0.00 | 0.00 | 0.16 | 1.46 | 0.03 | 0.00 |
|  | *Caldicoprobacter* |  | 0.00 | 0.00 | 0.00 | 0.00 | 0.00 | 0.00 | 0.00 | 0.00 | 0.00 | 0.01 |  | 0.00 | 0.00 | 0.00 | 0.00 | 0.01 | 0.00 |
|  | *Caloramator* |  | 0.00 | 0.00 | 0.09 | 0.20 | 0.00 | 0.00 | 0.25 | 0.00 | 0.00 | 0.26 |  | 0.00 | 0.00 | 0.14 | 0.00 | 0.04 | 0.00 |
|  | *Clostridium* |  | 0.35 | 0.04 | 0.37 | 0.18 | 0.34 | 0.00 | 0.58 | 0.14 | 0.25 | 0.50 |  | 0.20 | 0.00 | 0.59 | 0.00 | 0.08 | 0.00 |
|  | *Clostridium* | *celatum* | 0.33 | 0.14 | 0.00 | 0.00 | 0.00 | 0.00 | 0.00 | 0.14 | 0.00 | 0.00 |  | 0.00 | 0.00 | 0.00 | 0.00 | 0.00 | 0.00 |
|  | *Clostridium* | *bowmanii* | 0.23 | 0.00 | 0.00 | 0.29 | 0.09 | 0.46 | 0.35 | 0.26 | 0.25 | 0.19 |  | 0.00 | 0.00 | 0.25 | 0.00 | 0.00 | 0.00 |
|  | *Clostridium* | *butyricum* | 0.37 | 0.37 | 0.17 | 0.16 | 0.24 | 0.00 | 1.05 | 0.26 | 0.44 | 1.10 |  | 0.00 | 0.00 | 0.81 | 0.00 | 0.04 | 0.00 |
|  | *Clostridium* | *tyrobutyricum* | 0.00 | 0.15 | 0.00 | 0.00 | 0.00 | 0.00 | 0.19 | 0.00 | 0.20 | 0.11 |  | 0.00 | 0.00 | 0.09 | 0.00 | 0.00 | 0.00 |
|  | *Clostridium* | *subterminale* | 0.00 | 0.10 | 0.00 | 0.00 | 0.00 | 0.00 | 0.00 | 0.00 | 0.00 | 0.00 |  | 0.00 | 0.00 | 0.00 | 0.00 | 0.00 | 0.00 |
|  | *Clostridium* |  | 0.00 | 0.19 | 0.00 | 0.00 | 0.00 | 0.00 | 0.00 | 0.00 | 0.00 | 0.00 |  | 0.00 | 0.00 | 0.15 | 0.00 | 0.04 | 0.00 |
|  | *Clostridium* | *fimetarium* | 0.00 | 0.00 | 0.00 | 0.00 | 0.00 | 0.00 | 0.00 | 0.00 | 0.00 | 0.00 |  | 0.00 | 0.00 | 0.00 | 0.00 | 0.05 | 0.00 |
|  | *Clostridium* | *hungatei* | 0.00 | 0.00 | 0.00 | 0.00 | 0.00 | 0.00 | 0.22 | 0.00 | 0.00 | 0.00 |  | 0.00 | 0.00 | 0.15 | 0.00 | 0.00 | 0.00 |
|  | *Clostridium* | *papyrosolvens* | 0.00 | 0.00 | 0.00 | 0.00 | 0.00 | 0.00 | 0.00 | 0.37 | 0.00 | 0.00 |  | 0.00 | 0.00 | 0.00 | 0.00 | 0.00 | 0.00 |
|  | *Clostridium* | *intestinale* | 0.00 | 0.00 | 0.00 | 0.00 | 0.00 | 0.00 | 0.00 | 0.00 | 0.00 | 0.00 |  | 0.00 | 0.00 | 0.00 | 0.00 | 0.00 | 0.00 |
|  | *Clostridium* | *pasteurianum* | 0.00 | 0.00 | 0.00 | 0.00 | 0.00 | 0.00 | 0.00 | 0.00 | 0.00 | 0.00 |  | 0.00 | 0.00 | 0.00 | 0.00 | 0.00 | 0.00 |
|  | *Clostridium* | *acetobutylicum* | 0.00 | 0.00 | 0.00 | 0.00 | 0.00 | 0.00 | 0.00 | 0.00 | 0.00 | 0.05 |  | 0.00 | 0.00 | 0.00 | 0.00 | 0.00 | 0.00 |
|  | *Clostridium* | *ghonii* | 0.00 | 0.00 | 0.00 | 0.00 | 0.00 | 0.00 | 0.00 | 0.00 | 0.00 | 0.00 |  | 0.00 | 0.00 | 0.00 | 0.00 | 0.00 | 0.00 |
|  | *Clostridium* | *bifermentans* | 0.00 | 0.00 | 0.00 | 0.00 | 0.00 | 0.00 | 0.00 | 0.00 | 0.00 | 0.06 |  | 0.00 | 0.00 | 0.00 | 0.00 | 0.00 | 0.00 |
|  | *Coprococcus* |  | 0.00 | 0.00 | 0.00 | 0.00 | 0.00 | 0.00 | 0.35 | 0.07 | 0.21 | 0.12 |  | 0.18 | 0.36 | 0.00 | 0.00 | 0.00 | 0.00 |
|  | *Desulfosporosinus* | *meridiei* | 0.00 | 0.00 | 0.00 | 0.00 | 0.00 | 0.00 | 0.00 | 0.00 | 0.13 | 0.00 |  | 0.00 | 0.00 | 0.06 | 0.00 | 0.00 | 0.00 |
|  | *Desulfosporosinus* |  | 0.00 | 0.00 | 0.04 | 0.00 | 0.00 | 0.00 | 0.00 | 0.00 | 0.03 | 0.00 |  | 0.00 | 0.00 | 0.00 | 0.00 | 0.03 | 0.00 |
|  | *Desulfotomaculum* | *aeronauticum* | 0.00 | 0.00 | 0.00 | 0.00 | 0.00 | 0.00 | 0.00 | 0.00 | 0.00 | 0.00 |  | 0.00 | 0.00 | 0.03 | 0.00 | 0.00 | 0.00 |
|  | *Dialister* |  | 0.00 | 0.00 | 0.00 | 0.00 | 0.00 | 0.00 | 0.00 | 0.00 | 0.00 | 0.00 |  | 0.33 | 0.00 | 0.00 | 0.00 | 0.00 | 0.00 |
|  | *Epulopiscium* |  | 0.00 | 0.00 | 0.00 | 0.00 | 0.00 | 0.00 | 0.00 | 0.00 | 0.00 | 0.01 |  | 0.00 | 0.00 | 0.00 | 0.00 | 0.00 | 0.00 |
|  | *Finegoldia* |  | 0.00 | 0.00 | 0.00 | 0.00 | 0.00 | 0.00 | 0.00 | 0.00 | 0.00 | 0.00 |  | 0.00 | 0.00 | 0.00 | 0.00 | 0.00 | 0.00 |
|  | *Fusibacter* |  | 0.00 | 0.00 | 0.00 | 0.00 | 0.00 | 0.00 | 0.00 | 0.00 | 0.00 | 0.00 |  | 0.00 | 0.00 | 0.00 | 0.00 | 0.00 | 0.00 |
|  | *G07* |  | 0.00 | 0.00 | 0.00 | 0.00 | 0.00 | 0.00 | 0.00 | 0.00 | 0.00 | 0.00 |  | 0.00 | 0.00 | 0.04 | 0.00 | 0.00 | 0.00 |
|  | *Geosporobacter_Thermotalea* |  | 0.00 | 0.00 | 0.00 | 0.00 | 0.00 | 0.00 | 0.00 | 0.00 | 0.00 | 0.00 |  | 0.00 | 0.00 | 0.00 | 0.00 | 0.00 | 0.00 |
|  | *Gracilibacter* |  | 0.00 | 0.23 | 0.00 | 0.00 | 0.00 | 0.00 | 0.00 | 0.00 | 0.00 | 0.00 |  | 0.00 | 0.00 | 0.00 | 0.00 | 0.00 | 0.00 |
|  | *Oribacterium* |  | 0.00 | 0.00 | 0.00 | 0.00 | 0.00 | 0.00 | 0.00 | 0.00 | 0.00 | 0.00 |  | 0.31 | 0.00 | 0.00 | 0.00 | 0.00 | 0.00 |
|  | *Oxobacter* |  | 0.00 | 0.00 | 0.00 | 0.00 | 0.00 | 0.00 | 0.00 | 0.00 | 0.00 | 0.00 |  | 0.00 | 0.00 | 0.00 | 0.00 | 0.00 | 0.00 |
|  | *Pelosinus* |  | 0.00 | 0.00 | 0.00 | 0.00 | 0.21 | 0.00 | 0.37 | 0.00 | 0.59 | 0.05 |  | 0.07 | 0.00 | 0.14 | 0.00 | 0.05 | 0.00 |
|  | *Pelotomaculum* |  | 0.00 | 0.00 | 0.00 | 0.00 | 0.00 | 0.00 | 0.00 | 0.00 | 0.00 | 0.00 |  | 0.00 | 0.00 | 0.00 | 0.00 | 0.00 | 0.00 |
|  | *Peptoniphilus* |  | 0.00 | 0.00 | 0.00 | 0.00 | 0.00 | 0.00 | 0.00 | 0.00 | 0.00 | 0.00 |  | 0.15 | 0.00 | 0.00 | 0.00 | 0.00 | 0.00 |
|  | *Proteiniclasticum* |  | 0.00 | 0.00 | 0.00 | 0.00 | 0.00 | 0.00 | 0.00 | 0.00 | 0.00 | 0.00 |  | 0.00 | 0.00 | 0.00 | 0.00 | 0.00 | 0.00 |
|  | *Ruminococcus* |  | 0.00 | 0.00 | 0.00 | 0.00 | 0.02 | 0.00 | 0.00 | 0.00 | 0.00 | 0.00 |  | 0.19 | 0.00 | 0.00 | 0.00 | 0.00 | 0.00 |
|  | *Ruminococcus* |  | 0.00 | 0.00 | 0.00 | 0.00 | 0.00 | 0.00 | 0.00 | 0.00 | 0.00 | 0.00 |  | 0.00 | 0.00 | 0.00 | 1.75 | 0.00 | 0.00 |
|  | *Sedimentibacter* |  | 0.00 | 0.08 | 0.00 | 0.00 | 0.00 | 0.00 | 0.00 | 0.00 | 0.00 | 0.00 |  | 0.00 | 0.00 | 0.00 | 0.00 | 0.00 | 0.00 |
|  | *Selenomonas* | *lacticifex* | 0.00 | 0.00 | 0.00 | 0.00 | 0.00 | 0.00 | 0.00 | 0.00 | 0.00 | 0.00 |  | 0.00 | 0.00 | 0.00 | 0.00 | 0.00 | 0.00 |
|  | *Sporanaerobacter* |  | 0.00 | 0.00 | 0.00 | 0.00 | 0.00 | 0.00 | 0.00 | 0.04 | 0.00 | 0.00 |  | 0.00 | 0.00 | 0.00 | 0.00 | 0.00 | 0.00 |
|  | *Sporomusa* |  | 0.00 | 0.00 | 0.00 | 0.11 | 0.00 | 0.00 | 0.00 | 0.00 | 0.00 | 0.00 |  | 0.00 | 0.00 | 0.00 | 0.00 | 0.00 | 0.00 |
|  | *Sporotomaculum* |  | 0.00 | 0.00 | 0.00 | 0.03 | 0.00 | 0.00 | 0.00 | 0.00 | 0.00 | 0.00 |  | 0.00 | 0.00 | 0.00 | 0.00 | 0.00 | 0.00 |
|  | *Symbiobacterium* |  | 0.00 | 0.00 | 0.00 | 0.11 | 0.00 | 0.00 | 0.09 | 0.00 | 0.00 | 0.00 |  | 0.00 | 0.00 | 0.05 | 0.00 | 0.07 | 0.00 |
|  | *Symbiobacterium* | *thermophilum* | 0.00 | 0.00 | 0.00 | 0.00 | 0.00 | 0.00 | 0.00 | 0.00 | 0.00 | 0.00 |  | 0.11 | 0.00 | 0.00 | 0.00 | 0.00 | 0.00 |
|  | *Syntrophomonas* |  | 0.00 | 0.00 | 0.00 | 0.00 | 0.00 | 0.00 | 0.00 | 0.00 | 0.00 | 0.00 |  | 0.00 | 0.00 | 0.06 | 0.00 | 0.00 | 0.00 |
|  | *Tepidibacter* |  | 0.10 | 0.12 | 0.07 | 0.00 | 0.07 | 0.00 | 0.00 | 0.10 | 0.05 | 0.00 |  | 0.27 | 0.00 | 0.25 | 0.00 | 0.03 | 0.00 |
|  | *Tissierella_Soehngenia* |  | 0.00 | 0.00 | 0.00 | 0.00 | 0.00 | 0.00 | 0.00 | 0.00 | 0.00 | 0.00 |  | 0.00 | 0.00 | 0.00 | 0.00 | 0.00 | 0.00 |
|  | *Veillonella* | *parvula* | 0.00 | 0.00 | 0.00 | 0.00 | 0.00 | 0.00 | 0.00 | 0.00 | 0.00 | 0.00 |  | 4.72 | 0.00 | 0.00 | 0.00 | 0.00 | 0.00 |
|  | *Veillonella* |  | 0.00 | 0.00 | 0.00 | 0.00 | 0.00 | 0.00 | 0.00 | 0.00 | 0.00 | 0.00 |  | 0.52 | 0.14 | 0.00 | 0.00 | 0.00 | 0.00 |
|  | *WAL_1855D* |  | 0.00 | 0.00 | 0.00 | 0.00 | 0.00 | 0.00 | 0.00 | 0.00 | 0.00 | 0.00 |  | 0.00 | 0.00 | 0.00 | 0.00 | 0.00 | 0.00 |
| *Desulfurococcales* |  |  | 0.00 | 1.68 | 0.60 | 0.57 | 4.91 | 0.00 | 0.72 | 0.00 | 3.68 | 0.70 |  | 0.00 | 0.00 | 0.00 | 0.00 | 0.79 | 0.09 |
|  | *Aeropyrum* |  | 0.00 | 1.14 | 0.46 | 0.57 | 4.05 | 0.00 | 0.62 | 0.00 | 3.43 | 0.70 |  | 0.00 | 0.00 | 0.00 | 0.00 | 0.63 | 0.09 |
| *Enterobacteriales* |  |  | 0.00 | 0.00 | 0.00 | 0.00 | 0.00 | 0.00 | 0.00 | 0.00 | 0.00 | 0.00 |  | 0.00 | 0.00 | 0.00 | 17.25 | 0.00 | 0.00 |
|  | *Enterobacter* |  | 0.00 | 0.00 | 0.00 | 0.00 | 0.00 | 0.00 | 0.00 | 0.02 | 0.00 | 0.00 |  | 0.00 | 0.00 | 0.00 | 0.00 | 0.00 | 0.00 |
|  | *Erwinia* |  | 0.00 | 0.00 | 0.00 | 0.00 | 0.00 | 0.00 | 0.00 | 0.00 | 0.00 | 0.00 |  | 0.00 | 0.00 | 0.00 | 0.00 | 0.00 | 0.00 |
|  | *Escherichia* | *coli* | 0.00 | 0.00 | 0.00 | 0.00 | 0.00 | 0.00 | 0.00 | 0.00 | 0.00 | 0.00 |  | 0.00 | 0.00 | 0.00 | 0.00 | 0.00 | 0.00 |
|  | *Rahnella* | *aquatilis* | 0.00 | 0.00 | 0.00 | 0.00 | 0.00 | 0.00 | 0.00 | 0.00 | 0.00 | 0.00 |  | 0.00 | 0.00 | 0.00 | 0.00 | 0.00 | 0.00 |
|  | *Yersinia* |  | 0.00 | 0.00 | 0.00 | 0.00 | 0.00 | 0.00 | 0.00 | 0.00 | 0.00 | 0.00 |  | 0.00 | 0.00 | 0.00 | 0.00 | 0.02 | 0.00 |
| *envOPS12* |  |  | 8.78 | 7.45 | 1.73 | 1.64 | 6.09 | 1.50 | 2.44 | 1.40 | 3.04 | 1.44 |  | 0.44 | 6.77 | 5.08 | 3.51 | 5.20 | 0.00 |
| *Gaiellales* |  |  | 0.89 | 0.73 | 2.87 | 5.21 | 0.94 | 2.27 | 4.09 | 4.44 | 1.54 | 2.64 |  | 1.81 | 1.58 | 2.09 | 0.00 | 1.64 | 0.67 |
|  | *Gaiella* | *occulta* | 0.00 | 0.00 | 0.00 | 0.09 | 0.00 | 0.00 | 0.00 | 0.00 | 0.00 | 0.00 |  | 0.00 | 0.09 | 0.11 | 0.00 | 0.11 | 0.00 |
| *Gallionellales* | *Gallionella* |  | 0.00 | 0.00 | 0.21 | 0.13 | 0.00 | 0.00 | 0.00 | 0.00 | 0.00 | 0.11 |  | 0.00 | 0.00 | 0.42 | 0.00 | 0.02 | 0.00 |
| *GCA004* |  |  | 2.54 | 2.90 | 2.27 | 2.42 | 1.68 | 0.24 | 3.28 | 0.84 | 2.31 | 1.68 |  | 0.11 | 1.04 | 3.52 | 6.14 | 2.89 | 1.33 |
| *iii1-15* |  |  | 2.54 | 2.49 | 2.82 | 3.41 | 1.08 | 3.54 | 1.79 | 3.90 | 1.25 | 2.26 |  | 1.53 | 6.63 | 3.61 | 2.34 | 4.68 | 0.98 |
| *Lactobacillales* |  |  | 0.00 | 0.04 | 0.00 | 0.12 | 0.33 | 0.15 | 0.13 | 0.14 | 0.11 | 0.11 |  | 6.28 | 1.35 | 0.07 | 3.22 | 0.18 | 4.04 |
|  | *Enterococcus* |  | 0.00 | 0.00 | 0.00 | 0.00 | 0.00 | 0.00 | 0.00 | 0.00 | 0.00 | 0.00 |  | 0.00 | 0.00 | 0.00 | 0.00 | 0.00 | 0.00 |
|  | *Granulicatella* |  | 0.00 | 0.00 | 0.00 | 0.00 | 0.00 | 0.00 | 0.00 | 0.00 | 0.00 | 0.00 |  | 0.00 | 0.00 | 0.00 | 0.00 | 0.00 | 0.00 |
|  | *Lactobacillus* | *helveticus* | 0.00 | 0.04 | 0.00 | 0.12 | 0.21 | 0.15 | 0.09 | 0.05 | 0.08 | 0.11 |  | 0.00 | 0.99 | 0.00 | 0.00 | 0.00 | 0.10 |
|  | *Lactobacillus* |  | 0.00 | 0.00 | 0.00 | 0.00 | 0.12 | 0.00 | 0.04 | 0.06 | 0.00 | 0.00 |  | 0.00 | 0.36 | 0.07 | 0.00 | 0.00 | 0.00 |
|  | *Lactobacillus* | *salivarius* | 0.00 | 0.00 | 0.00 | 0.00 | 0.00 | 0.00 | 0.00 | 0.00 | 0.03 | 0.00 |  | 0.00 | 0.00 | 0.00 | 0.00 | 0.00 | 0.00 |
|  | *Lactobacillus* | *zeae* | 0.00 | 0.00 | 0.00 | 0.00 | 0.00 | 0.00 | 0.00 | 0.00 | 0.00 | 0.00 |  | 0.00 | 0.00 | 0.00 | 0.88 | 0.00 | 0.00 |
|  | *Leuconostoc* |  | 0.00 | 0.00 | 0.00 | 0.00 | 0.00 | 0.00 | 0.00 | 0.00 | 0.00 | 0.00 |  | 0.00 | 0.00 | 0.00 | 0.00 | 0.00 | 0.00 |
|  | *Streptococcus* | *infantis* | 0.00 | 0.00 | 0.00 | 0.00 | 0.00 | 0.00 | 0.00 | 0.03 | 0.00 | 0.00 |  | 2.92 | 0.00 | 0.00 | 2.34 | 0.11 | 3.94 |
|  | *Streptococcus* |  | 0.00 | 0.00 | 0.00 | 0.00 | 0.00 | 0.00 | 0.00 | 0.00 | 0.00 | 0.00 |  | 3.09 | 0.00 | 0.00 | 0.00 | 0.06 | 0.00 |
| *Methanosarcinales* |  |  | 0.85 | 1.01 | 0.14 | 1.67 | 1.12 | 0.13 | 0.87 | 0.00 | 2.25 | 0.64 |  | 0.18 | 0.00 | 0.19 | 0.00 | 0.27 | 0.00 |
|  | *Methanosarcina* |  | 3.00 | 8.45 | 0.00 | 0.74 | 12.62 | 0.18 | 1.27 | 0.00 | 8.49 | 1.04 |  | 0.00 | 0.00 | 0.40 | 0.00 | 0.98 | 0.00 |
| *Myxococcales* |  |  | 0.64 | 0.49 | 1.71 | 1.61 | 1.26 | 4.67 | 1.64 | 1.93 | 1.00 | 1.94 |  | 1.30 | 1.13 | 1.70 | 0.88 | 2.50 | 0.00 |
|  | *Sorangium* | *cellulosum* | 0.00 | 0.00 | 0.00 | 0.00 | 0.00 | 0.00 | 0.00 | 0.00 | 0.00 | 0.00 |  | 0.00 | 0.00 | 0.07 | 0.00 | 0.00 | 0.00 |
|  | *Plesiocystis* |  | 0.00 | 0.00 | 0.00 | 0.00 | 0.00 | 0.00 | 0.00 | 0.00 | 0.00 | 0.00 |  | 0.00 | 0.00 | 0.00 | 0.00 | 0.04 | 0.00 |
|  | *Nannocystis* |  | 0.00 | 0.00 | 0.00 | 0.00 | 0.02 | 0.00 | 0.00 | 0.00 | 0.00 | 0.05 |  | 0.00 | 0.09 | 0.00 | 0.00 | 0.04 | 0.00 |
|  | *Myxococcus* |  | 0.00 | 0.00 | 0.00 | 0.00 | 0.00 | 0.00 | 0.00 | 0.00 | 0.00 | 0.00 |  | 0.00 | 0.00 | 0.00 | 0.00 | 0.00 | 0.00 |
|  | *Haliangium* |  | 0.00 | 0.00 | 0.00 | 0.00 | 0.00 | 0.00 | 0.00 | 0.00 | 0.00 | 0.00 |  | 0.00 | 0.00 | 0.00 | 0.00 | 0.00 | 0.00 |
|  | *Cystobacter* |  | 0.00 | 0.00 | 0.00 | 0.00 | 0.00 | 0.00 | 0.00 | 0.00 | 0.00 | 0.00 |  | 0.00 | 0.00 | 0.00 | 0.00 | 0.00 | 0.00 |
|  | *Cystobacter* | *fuscus* | 0.00 | 0.00 | 0.00 | 0.00 | 0.00 | 0.00 | 0.00 | 0.00 | 0.00 | 0.00 |  | 0.00 | 0.00 | 0.00 | 0.00 | 0.00 | 0.00 |
|  | *Corallococcus* | *exiguus* | 0.00 | 0.00 | 0.00 | 0.00 | 0.00 | 0.00 | 0.00 | 0.00 | 0.00 | 0.07 |  | 0.00 | 0.00 | 0.00 | 0.00 | 0.00 | 0.00 |
|  | *Anaeromyxobacter* |  | 0.00 | 0.44 | 3.92 | 0.00 | 0.00 | 0.00 | 1.53 | 0.15 | 0.68 | 0.99 |  | 0.33 | 0.27 | 0.65 | 0.00 | 0.39 | 0.00 |
|  | *Aetherobacter* | *fasciculatus* | 0.00 | 0.00 | 0.00 | 0.00 | 0.00 | 0.00 | 0.00 | 0.17 | 0.24 | 0.22 |  | 0.08 | 0.77 | 0.24 | 0.00 | 0.20 | 0.00 |
| *Neisseriales* |  |  | 0.00 | 0.00 | 0.15 | 0.00 | 0.06 | 0.00 | 0.00 | 0.00 | 0.00 | 0.00 |  | 30.12 | 0.09 | 0.00 | 0.00 | 0.00 | 0.00 |
| *Neisseriales* | *Chromobacterium* |  | 0.00 | 0.00 | 0.00 | 0.00 | 0.00 | 0.00 | 0.00 | 0.00 | 0.00 | 0.00 |  | 0.00 | 0.00 | 0.00 | 0.00 | 0.00 | 0.00 |
|  | *Eikenella* |  | 0.00 | 0.00 | 0.00 | 0.00 | 0.00 | 0.00 | 0.00 | 0.00 | 0.00 | 0.00 |  | 0.16 | 0.00 | 0.00 | 0.00 | 0.00 | 0.00 |
|  | *Neisseria* |  | 0.00 | 0.00 | 0.14 | 0.00 | 0.00 | 0.00 | 0.00 | 0.00 | 0.00 | 0.00 |  | 24.36 | 0.00 | 0.00 | 0.00 | 0.00 | 0.00 |
|  | *Neisseria* | *subflava* | 0.00 | 0.00 | 0.00 | 0.00 | 0.00 | 0.00 | 0.00 | 0.00 | 0.00 | 0.00 |  | 5.60 | 0.00 | 0.00 | 0.00 | 0.00 | 0.00 |
|  | *Pseudogulbenkiania* | *subflava* | 0.00 | 0.00 | 0.00 | 0.00 | 0.06 | 0.00 | 0.00 | 0.00 | 0.00 | 0.00 |  | 0.00 | 0.00 | 0.00 | 0.00 | 0.00 | 0.00 |
|  | *Snodgrassella* | *alvi* | 0.00 | 0.00 | 0.00 | 0.00 | 0.00 | 0.00 | 0.00 | 0.00 | 0.00 | 0.00 |  | 0.00 | 0.09 | 0.00 | 0.00 | 0.00 | 0.00 |
|  | *Vogesella* |  | 0.00 | 0.00 | 0.00 | 0.00 | 0.00 | 0.00 | 0.00 | 0.00 | 0.00 | 0.00 |  | 0.00 | 0.00 | 0.00 | 0.00 | 0.00 | 0.00 |
| *Nitrospirales* |  |  | 1.39 | 0.59 | 0.73 | 0.47 | 0.42 | 1.17 | 0.08 | 0.57 | 0.35 | 0.03 |  | 0.00 | 0.59 | 0.17 | 0.00 | 0.51 | 1.20 |
|  | *#47209* |  | 0.00 | 0.00 | 0.25 | 0.12 | 0.37 | 0.00 | 0.13 | 0.00 | 0.18 | 0.00 |  | 0.00 | 0.00 | 0.16 | 0.00 | 0.16 | 0.00 |
|  | *DCE29* |  | 0.00 | 0.00 | 0.00 | 0.00 | 0.04 | 0.00 | 0.00 | 0.00 | 0.00 | 0.00 |  | 0.00 | 0.00 | 0.00 | 0.00 | 0.00 | 0.00 |
|  | *GOUTA19* |  | 0.38 | 0.00 | 0.16 | 0.37 | 1.28 | 0.00 | 0.19 | 0.00 | 0.76 | 0.00 |  | 0.00 | 0.59 | 0.12 | 16.67 | 0.72 | 0.00 |
|  | *JG37-AG-70* |  | 0.10 | 0.00 | 0.00 | 0.00 | 0.00 | 0.00 | 0.00 | 0.00 | 0.00 | 0.00 |  | 0.00 | 0.00 | 0.03 | 0.00 | 0.00 | 0.00 |
|  | *LCP-6* |  | 0.00 | 0.00 | 0.00 | 0.00 | 0.00 | 0.00 | 0.00 | 0.00 | 0.00 | 0.00 |  | 0.00 | 0.00 | 0.00 | 0.00 | 0.02 | 0.00 |
|  | *Nitrospira* |  | 0.96 | 1.15 | 0.63 | 0.63 | 0.93 | 0.46 | 0.29 | 1.43 | 0.33 | 0.36 |  | 0.00 | 1.40 | 0.44 | 0.00 | 0.35 | 0.00 |
|  | *Nitrospira* | *calida* | 0.00 | 0.00 | 0.00 | 0.00 | 0.00 | 0.04 | 0.00 | 0.00 | 0.00 | 0.00 |  | 0.00 | 0.00 | 0.00 | 0.00 | 0.00 | 0.00 |
| *Pseudomonadales* |  |  | 0.00 | 0.00 | 1.66 | 0.21 | 0.06 | 3.63 | 0.00 | 0.03 | 0.00 | 0.00 |  | 0.00 | 0.00 | 0.00 | 0.00 | 0.00 | 0.00 |
|  | *Acinetobacter* |  | 0.00 | 0.00 | 0.00 | 0.00 | 0.00 | 0.00 | 0.00 | 0.00 | 0.00 | 0.00 |  | 0.00 | 0.00 | 0.00 | 0.00 | 0.00 | 0.00 |
|  | *Acinetobacter* | *rhizosphaerae* | 0.00 | 0.00 | 0.00 | 0.00 | 0.00 | 0.00 | 0.00 | 0.00 | 0.00 | 0.00 |  | 0.00 | 0.00 | 0.00 | 0.00 | 0.00 | 0.00 |
|  | *Acinetobacter* | *johnsonii* | 0.00 | 0.00 | 0.00 | 0.00 | 0.00 | 0.00 | 0.00 | 0.00 | 0.00 | 0.00 |  | 0.08 | 0.00 | 0.00 | 1.17 | 0.00 | 0.00 |
| *Pseudanabaenales* | *Arthronema* |  | 0.00 | 0.00 | 0.00 | 0.02 | 0.05 | 0.00 | 0.00 | 0.00 | 0.76 | 0.00 |  | 0.00 | 0.00 | 0.00 | 0.00 | 0.00 | 0.00 |
|  | *Enhydrobacter* |  | 0.00 | 0.00 | 0.00 | 0.00 | 0.00 | 0.00 | 0.00 | 0.00 | 0.00 | 0.00 |  | 0.41 | 1.08 | 0.07 | 0.00 | 0.00 | 1.07 |
|  | *Leptolyngbya* |  | 0.81 | 0.00 | 0.10 | 0.39 | 0.20 | 1.47 | 0.27 | 0.32 | 0.90 | 0.22 |  | 0.17 | 0.36 | 0.21 | 0.00 | 0.03 | 0.00 |
|  | *Nodosilinea* | *nodulosa* | 0.00 | 0.00 | 0.00 | 0.00 | 0.00 | 0.00 | 0.00 | 0.00 | 0.15 | 0.00 |  | 0.00 | 0.00 | 0.00 | 0.00 | 0.00 | 0.00 |
|  | *Perlucidibaca* |  | 0.00 | 0.00 | 0.00 | 0.00 | 0.00 | 0.00 | 0.00 | 0.00 | 0.00 | 0.00 |  | 0.00 | 0.00 | 0.00 | 0.00 | 0.04 | 0.00 |
|  | *Pseudomonas* |  | 0.04 | 0.00 | 0.08 | 0.00 | 0.00 | 0.00 | 0.00 | 0.00 | 0.20 | 0.00 |  | 0.00 | 0.00 | 0.00 | 0.00 | 0.00 | 0.00 |
|  | *Pseudomonas* | *alcaligenes* | 0.00 | 0.00 | 0.00 | 0.00 | 0.00 | 0.00 | 0.00 | 0.00 | 0.00 | 0.00 |  | 0.00 | 0.18 | 0.00 | 0.00 | 0.00 | 0.00 |
|  | *Pseudomonas* | *mendocina* | 0.00 | 0.00 | 0.00 | 0.00 | 0.00 | 0.00 | 0.00 | 0.00 | 0.00 | 0.00 |  | 0.00 | 0.00 | 0.00 | 0.00 | 0.00 | 0.00 |
|  | *Pseudomonas* | *umsongensis* | 0.00 | 0.00 | 0.00 | 0.00 | 0.15 | 0.00 | 0.00 | 0.00 | 0.00 | 0.09 |  | 0.00 | 0.00 | 0.00 | 0.00 | 0.00 | 0.00 |
|  | *Synechococcus* | *elongatus* | 0.00 | 0.00 | 0.00 | 0.00 | 0.00 | 0.00 | 0.00 | 0.00 | 0.00 | 0.00 |  | 0.00 | 0.00 | 0.00 | 0.00 | 0.00 | 0.00 |
| *RB41* |  |  | 0.96 | 4.85 | 1.37 | 0.70 | 2.05 | 2.49 | 0.48 | 2.31 | 0.18 | 1.43 |  | 0.33 | 0.59 | 1.94 | 1.46 | 1.54 | 0.00 |
| *Rhizobiales* |  |  | 2.95 | 4.73 | 2.80 | 5.99 | 3.89 | 6.87 | 6.10 | 8.23 | 6.81 | 5.35 |  | 1.60 | 6.09 | 5.73 | 1.75 | 3.86 | 0.50 |
|  | *Afifella* |  | 0.00 | 0.05 | 0.00 | 0.00 | 0.00 | 0.00 | 0.00 | 0.00 | 0.00 | 0.10 |  | 0.00 | 0.00 | 0.09 | 0.00 | 0.00 | 0.00 |
|  | *Afipia* |  | 0.00 | 0.00 | 0.00 | 0.00 | 0.00 | 0.00 | 0.00 | 0.00 | 0.00 | 0.00 |  | 0.00 | 0.00 | 0.00 | 0.00 | 0.00 | 0.00 |
|  | *Agrobacterium* |  | 0.15 | 0.00 | 0.00 | 0.00 | 0.00 | 0.00 | 0.00 | 0.00 | 0.00 | 0.00 |  | 0.00 | 0.00 | 0.00 | 0.00 | 0.00 | 0.00 |
|  | *Agrobacterium* | *sullae* | 0.00 | 0.00 | 0.00 | 0.00 | 0.00 | 0.00 | 0.00 | 0.33 | 0.00 | 0.00 |  | 0.00 | 0.00 | 0.00 | 0.00 | 0.00 | 0.00 |
|  | *Balneimonas* |  | 0.00 | 0.00 | 0.23 | 0.55 | 0.00 | 0.86 | 0.00 | 0.57 | 0.00 | 0.40 |  | 0.24 | 0.00 | 0.31 | 1.75 | 0.03 | 0.00 |
|  | *Beijerinckia* |  | 0.00 | 0.00 | 0.00 | 0.00 | 0.00 | 0.59 | 0.00 | 0.00 | 0.00 | 0.09 |  | 0.00 | 0.00 | 0.00 | 0.00 | 0.00 | 0.00 |
|  | *Blastobacter* | *denitrificans* | 0.00 | 0.00 | 0.00 | 0.00 | 0.00 | 0.00 | 0.00 | 0.00 | 0.00 | 0.00 |  | 0.00 | 0.00 | 0.00 | 0.00 | 0.00 | 0.00 |
|  | *Bosea* | *genosp.* | 0.00 | 0.00 | 0.00 | 0.00 | 0.00 | 0.00 | 0.00 | 0.00 | 0.16 | 0.25 |  | 0.00 | 0.00 | 0.26 | 0.00 | 0.10 | 0.00 |
|  | *Bradyrhizobium* |  | 1.02 | 0.72 | 0.92 | 0.81 | 0.77 | 1.19 | 1.18 | 1.56 | 1.08 | 0.71 |  | 0.36 | 2.80 | 1.64 | 0.00 | 0.55 | 0.00 |
|  | *Devosia* |  | 0.00 | 0.11 | 0.14 | 0.18 | 0.26 | 0.00 | 0.22 | 0.52 | 0.19 | 1.04 |  | 0.00 | 0.54 | 0.36 | 0.00 | 0.54 | 0.00 |
|  | *Ensifer* | *adhaerens* | 0.00 | 0.00 | 0.00 | 0.00 | 0.00 | 0.00 | 0.00 | 0.00 | 0.00 | 0.00 |  | 0.00 | 0.00 | 0.00 | 0.00 | 0.00 | 0.00 |
|  | *Hyphomicrobium* |  | 0.38 | 0.50 | 0.00 | 0.59 | 0.22 | 0.35 | 0.48 | 0.35 | 0.57 | 0.43 |  | 0.07 | 0.00 | 0.38 | 0.00 | 0.17 | 0.00 |
|  | *Kaistia* |  | 0.00 | 0.00 | 0.00 | 0.00 | 0.10 | 0.00 | 0.05 | 0.00 | 0.00 | 0.21 |  | 0.00 | 0.18 | 0.00 | 0.00 | 0.00 | 0.00 |
|  | *Mesorhizobium* |  | 0.00 | 0.00 | 0.00 | 0.00 | 0.00 | 0.00 | 0.10 | 0.29 | 0.00 | 0.11 |  | 0.00 | 0.00 | 0.00 | 0.00 | 0.00 | 0.00 |
|  | *Methylobacterium* |  | 0.00 | 0.00 | 0.00 | 0.00 | 0.00 | 0.00 | 0.15 | 0.00 | 0.00 | 0.09 |  | 0.00 | 0.00 | 0.00 | 0.00 | 0.00 | 0.00 |
|  | *Methylobacterium* | *organophilum* | 0.00 | 0.00 | 0.00 | 0.00 | 0.00 | 0.00 | 0.00 | 0.00 | 0.00 | 0.00 |  | 0.00 | 0.00 | 0.00 | 0.00 | 0.00 | 0.00 |
|  | *Methylosinus* |  | 0.00 | 0.41 | 0.00 | 0.39 | 0.33 | 0.00 | 0.84 | 0.00 | 0.80 | 0.45 |  | 0.16 | 0.00 | 0.26 | 0.00 | 0.26 | 0.00 |
|  | *Pedomicrobium* |  | 0.00 | 0.14 | 0.00 | 0.12 | 0.00 | 0.00 | 0.00 | 0.00 | 0.00 | 0.00 |  | 0.16 | 0.00 | 0.00 | 0.00 | 0.00 | 0.00 |
|  | *Pleomorphomonas* |  | 0.00 | 0.00 | 0.00 | 0.00 | 0.00 | 0.00 | 0.00 | 0.00 | 0.00 | 0.00 |  | 0.00 | 0.00 | 0.00 | 0.00 | 0.00 | 0.00 |
|  | *Pleomorphomonas* | *oryzae* | 0.00 | 0.00 | 0.00 | 0.00 | 0.00 | 0.00 | 0.04 | 0.00 | 0.00 | 0.05 |  | 0.00 | 0.00 | 0.00 | 0.00 | 0.00 | 0.00 |
|  | *Rhizobium* |  | 0.00 | 0.00 | 0.00 | 0.00 | 0.24 | 0.00 | 0.00 | 0.00 | 0.00 | 0.00 |  | 0.00 | 0.00 | 0.00 | 0.00 | 0.00 | 0.00 |
|  | *Rhodoblastus* | *acidophilus* | 0.00 | 0.00 | 0.00 | 0.00 | 0.00 | 0.00 | 0.19 | 0.00 | 0.00 | 0.13 |  | 0.25 | 0.00 | 0.00 | 0.00 | 0.00 | 0.00 |
|  | *Rhodomicrobium* | *vannielii* | 0.00 | 0.12 | 0.00 | 0.00 | 0.00 | 0.29 | 0.12 | 0.08 | 0.00 | 0.00 |  | 0.14 | 0.00 | 0.00 | 0.00 | 0.00 | 0.00 |
|  | *Rhodoplanes* |  | 1.39 | 2.68 | 1.51 | 3.35 | 1.96 | 3.59 | 2.72 | 4.53 | 4.00 | 1.32 |  | 0.23 | 2.57 | 2.43 | 0.00 | 2.21 | 0.50 |
| *Rhodospirillales* |  |  | 1.81 | 0.49 | 1.07 | 6.58 | 0.77 | 0.24 | 2.84 | 1.97 | 0.68 | 0.56 |  | 0.73 | 2.44 | 1.65 | 0.00 | 2.54 | 0.00 |
|  | *Azospirillum* |  | 0.00 | 0.00 | 0.04 | 0.00 | 0.00 | 0.00 | 0.00 | 0.00 | 0.00 | 0.09 |  | 0.00 | 0.00 | 0.00 | 0.00 | 0.00 | 0.00 |
|  | *Azospirillum* | *brasilense* | 0.00 | 0.00 | 0.00 | 0.00 | 0.00 | 0.00 | 0.00 | 0.00 | 0.00 | 0.00 |  | 0.00 | 0.00 | 0.00 | 0.00 | 0.00 | 0.00 |
|  | *Defluviicoccus* | *vanus* | 0.00 | 0.00 | 0.00 | 0.14 | 0.07 | 0.00 | 0.00 | 0.00 | 0.00 | 0.00 |  | 0.00 | 0.00 | 0.00 | 0.00 | 0.00 | 0.00 |
|  | *Dongia* | *mobilis* | 0.00 | 0.07 | 0.00 | 0.00 | 0.17 | 0.00 | 0.00 | 0.00 | 0.00 | 0.00 |  | 0.00 | 0.36 | 0.00 | 0.00 | 0.10 | 0.00 |
|  | *Magnetospirillum* |  | 0.00 | 0.00 | 0.10 | 0.00 | 0.00 | 0.00 | 0.00 | 0.00 | 0.00 | 0.00 |  | 0.00 | 0.00 | 0.00 | 0.00 | 0.02 | 0.00 |
|  | *Reyranella* | *massiliensis* | 0.00 | 0.16 | 0.19 | 0.21 | 0.18 | 0.00 | 0.27 | 0.48 | 0.88 | 0.17 |  | 0.00 | 0.36 | 0.17 | 0.00 | 0.40 | 0.00 |
|  | *Roseococcus* |  | 0.00 | 0.00 | 0.00 | 0.35 | 0.00 | 0.00 | 0.00 | 0.00 | 0.00 | 0.00 |  | 0.00 | 0.00 | 0.00 | 0.00 | 0.00 | 0.00 |
|  | *Roseomonas* | *lacus* | 0.00 | 0.00 | 0.00 | 0.09 | 0.00 | 0.00 | 0.00 | 0.00 | 0.00 | 0.00 |  | 0.00 | 0.00 | 0.00 | 0.00 | 0.00 | 0.00 |
|  | *Roseomonas* |  | 0.00 | 0.00 | 0.20 | 0.00 | 0.00 | 0.00 | 0.22 | 0.13 | 0.00 | 0.16 |  | 0.00 | 0.00 | 0.00 | 0.00 | 0.00 | 0.00 |
|  | *Skermanella* |  | 0.94 | 0.10 | 0.33 | 0.00 | 0.07 | 0.00 | 0.00 | 0.00 | 0.00 | 0.07 |  | 0.19 | 0.00 | 0.58 | 0.00 | 0.00 | 0.00 |
| *Rickettsiales* |  |  | 0.00 | 0.00 | 0.06 | 0.00 | 0.08 | 0.00 | 0.00 | 0.00 | 0.00 | 0.00 |  | 0.00 | 0.27 | 0.00 | 0.00 | 0.00 | 35.64 |
|  | *Acanthamoeba* | *tubiashi* | 0.04 | 0.00 | 0.00 | 0.00 | 0.00 | 0.00 | 0.00 | 0.00 | 0.00 | 0.00 |  | 0.00 | 0.00 | 0.00 | 0.00 | 0.00 | 0.00 |
|  | *Prototheca* | *wickerhamii* | 0.08 | 0.00 | 0.00 | 0.00 | 0.00 | 0.00 | 0.00 | 0.00 | 0.00 | 0.00 |  | 0.00 | 0.00 | 0.00 | 0.00 | 0.00 | 0.00 |
| *Saprospirales* |  |  | 1.62 | 1.55 | 0.27 | 0.11 | 0.79 | 0.97 | 0.30 | 0.94 | 0.82 | 0.48 |  | 0.00 | 1.85 | 0.57 | 0.00 | 1.10 | 0.00 |
|  | *Chitinophaga* |  | 0.00 | 0.00 | 0.00 | 0.00 | 0.00 | 0.00 | 0.00 | 0.00 | 0.00 | 0.00 |  | 0.00 | 0.00 | 0.00 | 0.00 | 0.00 | 0.00 |
|  | *Flavihumibacter* |  | 0.00 | 0.00 | 0.00 | 0.00 | 0.00 | 0.00 | 0.00 | 0.00 | 0.00 | 0.00 |  | 0.00 | 0.00 | 0.00 | 0.00 | 0.00 | 0.00 |
|  | *Flavisolibacter* |  | 0.52 | 0.13 | 1.49 | 0.00 | 0.12 | 2.38 | 0.52 | 0.65 | 0.16 | 0.67 |  | 0.00 | 0.00 | 0.32 | 0.00 | 1.12 | 0.00 |
|  | *Haliscomenobacter* |  | 0.00 | 0.00 | 0.00 | 0.00 | 0.00 | 0.00 | 0.00 | 0.00 | 0.00 | 0.00 |  | 0.00 | 0.00 | 0.00 | 0.00 | 0.00 | 0.00 |
|  | *Lacibacter* | *cauensis* | 0.08 | 0.00 | 0.00 | 0.00 | 0.00 | 0.00 | 0.00 | 0.00 | 0.00 | 0.00 |  | 0.00 | 0.00 | 0.00 | 0.00 | 0.00 | 0.00 |
|  | *Niabella* |  | 0.00 | 0.00 | 0.00 | 0.00 | 0.00 | 0.00 | 0.00 | 0.00 | 0.00 | 0.11 |  | 0.00 | 0.00 | 0.00 | 0.00 | 0.00 | 0.00 |
|  | *Niastella* |  | 0.00 | 0.00 | 0.00 | 0.00 | 0.08 | 0.00 | 0.00 | 0.14 | 0.07 | 0.00 |  | 0.00 | 0.00 | 0.09 | 0.00 | 0.00 | 0.00 |
|  | *Parasegitibacter* | *luojiensis* | 0.00 | 0.00 | 0.00 | 0.00 | 0.00 | 0.00 | 0.00 | 0.00 | 0.00 | 0.08 |  | 0.00 | 0.00 | 0.00 | 0.00 | 0.00 | 0.00 |
|  | *Sediminibacterium* |  | 0.00 | 0.00 | 0.00 | 0.00 | 0.00 | 0.00 | 0.00 | 0.00 | 0.00 | 0.00 |  | 0.00 | 0.00 | 0.00 | 0.00 | 0.04 | 0.00 |
|  | *Segetibacter* |  | 0.00 | 0.00 | 0.13 | 0.00 | 0.00 | 0.11 | 0.00 | 0.10 | 0.11 | 0.10 |  | 0.00 | 0.00 | 0.00 | 0.00 | 0.00 | 0.68 |
|  | *Trachelomonas* | *volvocinopsis* | 0.00 | 0.00 | 0.00 | 0.00 | 0.00 | 0.00 | 0.00 | 0.00 | 0.00 | 0.00 |  | 0.00 | 0.00 | 0.00 | 0.00 | 0.00 | 0.00 |
| *SC-I-84* |  |  | 0.27 | 1.19 | 2.56 | 1.43 | 1.08 | 1.14 | 1.49 | 1.26 | 2.28 | 0.73 |  | 0.11 | 0.81 | 1.33 | 0.00 | 2.28 | 0.00 |
| *SJA-15* |  |  | 0.56 | 0.51 | 1.13 | 1.28 | 0.98 | 0.00 | 2.62 | 0.04 | 2.14 | 2.42 |  | 0.29 | 0.81 | 2.63 | 0.00 | 1.95 | 1.46 |
| *Solibacterales* |  |  | 1.50 | 2.40 | 4.33 | 0.95 | 2.65 | 3.74 | 1.36 | 1.15 | 0.65 | 2.00 |  | 0.27 | 0.50 | 1.30 | 0.00 | 1.25 | 0.61 |
|  | *Candidatus Solibacter* |  | 0.98 | 1.22 | 1.15 | 0.15 | 1.40 | 1.34 | 0.30 | 0.05 | 0.15 | 0.46 |  | 0.04 | 0.50 | 0.54 | 0.00 | 0.43 | 0.61 |
| *Solirubrobacterales* |  |  | 0.00 | 0.11 | 0.84 | 5.00 | 0.34 | 0.00 | 2.87 | 0.95 | 1.22 | 1.98 |  | 1.59 | 0.00 | 1.43 | 0.00 | 0.94 | 0.00 |
|  | *Conexibacter* |  | 0.00 | 0.00 | 0.00 | 0.00 | 0.00 | 0.00 | 0.00 | 0.00 | 0.00 | 0.00 |  | 0.00 | 0.00 | 0.00 | 0.00 | 0.00 | 0.00 |
|  | *Solirubrobacter* |  | 0.00 | 0.00 | 0.00 | 0.00 | 0.00 | 0.00 | 0.00 | 0.00 | 0.00 | 0.13 |  | 0.00 | 0.00 | 0.00 | 0.00 | 0.00 | 0.00 |
| *Sphingomonadales* |  |  | 1.50 | 1.40 | 2.64 | 0.91 | 1.25 | 11.47 | 1.45 | 3.28 | 0.46 | 3.87 |  | 0.05 | 2.44 | 3.71 | 0.00 | 4.08 | 0.46 |
|  | *Erythromicrobium* |  | 0.00 | 0.00 | 0.00 | 0.00 | 0.00 | 0.00 | 0.00 | 0.00 | 0.00 | 0.00 |  | 0.00 | 0.00 | 0.00 | 0.00 | 0.00 | 0.00 |
|  | *Kaistobacter* |  | 1.25 | 1.40 | 2.64 | 0.80 | 1.25 | 11.47 | 1.33 | 3.17 | 0.38 | 3.01 |  | 0.05 | 2.44 | 3.30 | 0.00 | 3.74 | 0.00 |
|  | *Novosphingobium* |  | 0.00 | 0.00 | 0.00 | 0.00 | 0.00 | 0.00 | 0.00 | 0.00 | 0.00 | 0.00 |  | 0.00 | 0.00 | 0.00 | 0.00 | 0.00 | 0.00 |
|  | *Sandaracinobacter* | *sibiricus* | 0.00 | 0.00 | 0.00 | 0.00 | 0.00 | 0.00 | 0.00 | 0.00 | 0.08 | 0.00 |  | 0.00 | 0.00 | 0.00 | 0.00 | 0.00 | 0.00 |
|  | *Sphingobium* |  | 0.00 | 0.00 | 0.00 | 0.00 | 0.00 | 0.00 | 0.00 | 0.00 | 0.00 | 0.11 |  | 0.00 | 0.00 | 0.00 | 0.00 | 0.00 | 0.00 |
|  | *Sphingomonas* |  | 0.00 | 0.00 | 0.00 | 0.00 | 0.00 | 0.00 | 0.13 | 0.00 | 0.00 | 0.37 |  | 0.00 | 0.00 | 0.06 | 0.00 | 0.00 | 0.00 |
|  | *Sphingopyxis* |  | 0.00 | 0.00 | 0.00 | 0.00 | 0.00 | 0.00 | 0.00 | 0.00 | 0.00 | 0.00 |  | 0.00 | 0.00 | 0.00 | 0.00 | 0.00 | 0.00 |
|  | *Sphingopyxis* | *alaskensis* | 0.00 | 0.00 | 0.00 | 0.00 | 0.00 | 0.00 | 0.00 | 0.00 | 0.00 | 0.00 |  | 0.00 | 0.00 | 0.00 | 0.00 | 0.00 | 0.00 |
|  | *Zymomonas* |  | 0.00 | 0.00 | 0.00 | 0.11 | 0.00 | 0.00 | 0.00 | 0.00 | 0.00 | 0.00 |  | 0.00 | 0.00 | 0.00 | 0.00 | 0.03 | 0.00 |

**Supplementary table 4**: relative abundance of *Bacteria* and *Archaea* in L-HeR and H-HeR soils at the taxonomic ranks of order, genus and species.

|  |  |  | L-HeR | | | | | | | | | |  | H-HeR | | | | | |
| --- | --- | --- | --- | --- | --- | --- | --- | --- | --- | --- | --- | --- | --- | --- | --- | --- | --- | --- | --- |
| Order | Genus | species | FR.1 | FR.2 | FR.10 | FR.13 | FR.15 | FR.16 | FR.27 | FR.28 | FR.31 | FR.32 |  | FR.7 | FR.8 | FR.9 | FR.17 | FR.18 | FR.19 |
| *Agaricales* | *Agaricus* |  | 0.00 | 0.00 | 11.87 | 0.00 | 0.00 | 0.00 | 0.00 | 0.00 | 0.00 | 0.00 |  | 0.00 | 0.00 | 0.00 | 0.00 | 0.00 | 0.00 |
|  |  | *flavicentrus* | 0.00 | 0.00 | 11.87 | 0.00 | 0.00 | 0.00 | 0.00 | 0.00 | 0.00 | 0.00 |  | 0.00 | 0.00 | 0.00 | 0.00 | 0.00 | 0.00 |
|  | *Bolbitius* |  | 1.82 | 0.22 | 0.00 | 0.00 | 1.04 | 0.00 | 0.00 | 0.00 | 0.40 | 0.00 |  | 0.00 | 0.00 | 0.00 | 0.00 | 0.00 | 0.00 |
|  |  | *coprophilus* | 1.45 | 0.22 | 0.00 | 0.00 | 0.85 | 0.00 | 0.00 | 0.00 | 0.40 | 0.00 |  | 0.00 | 0.00 | 0.00 | 0.00 | 0.00 | 0.00 |
|  | *Clitopilus* |  | 0.00 | 0.00 | 0.00 | 0.00 | 0.00 | 0.00 | 0.00 | 0.00 | 0.22 | 0.00 |  | 0.00 | 0.00 | 0.00 | 0.00 | 0.00 | 0.00 |
|  |  | *passeckerianus* | 0.00 | 0.00 | 0.00 | 0.00 | 0.00 | 0.00 | 0.00 | 0.00 | 0.22 | 0.00 |  | 0.00 | 0.00 | 0.00 | 0.00 | 0.00 | 0.00 |
|  | *Conocybe* |  | 0.00 | 0.00 | 0.00 | 0.00 | 0.00 | 0.00 | 0.00 | 19.39 | 0.00 | 0.00 |  | 0.00 | 0.00 | 0.00 | 0.00 | 0.00 | 0.00 |
|  |  | *apala* | 0.00 | 0.00 | 0.00 | 0.00 | 0.00 | 0.00 | 0.00 | 9.54 | 0.00 | 0.00 |  | 0.00 | 0.00 | 0.00 | 0.00 | 0.00 | 0.00 |
|  | *Coprinellus* |  | 1.83 | 0.00 | 0.00 | 0.00 | 0.00 | 0.00 | 0.00 | 0.00 | 0.00 | 0.00 |  | 0.00 | 0.00 | 0.00 | 0.00 | 0.00 | 0.54 |
|  |  | *verrucispermus* | 1.83 | 0.00 | 0.00 | 0.00 | 0.00 | 0.00 | 0.00 | 0.00 | 0.00 | 0.00 |  | 0.00 | 0.00 | 0.00 | 0.00 | 0.00 | 0.54 |
|  | *Entoloma* |  | 0.00 | 0.00 | 0.00 | 0.00 | 0.00 | 0.00 | 0.00 | 0.00 | 0.00 | 0.00 |  | 0.00 | 0.00 | 0.00 | 0.00 | 0.00 | 0.00 |
|  |  | *graphitipes f. cystidiatum* | 0.00 | 0.00 | 0.00 | 0.00 | 0.00 | 0.00 | 0.00 | 0.00 | 0.00 | 0.00 |  | 0.00 | 0.00 | 0.00 | 0.00 | 0.00 | 0.00 |
|  | *Lycoperdon* |  | 0.04 | 0.20 | 0.07 | 0.00 | 0.07 | 0.00 | 0.28 | 0.08 | 0.17 | 0.67 |  | 0.00 | 0.00 | 0.00 | 0.00 | 0.00 | 0.00 |
|  |  | *pratense* | 0.04 | 0.20 | 0.07 | 0.00 | 0.07 | 0.00 | 0.28 | 0.08 | 0.17 | 0.67 |  | 0.00 | 0.00 | 0.00 | 0.00 | 0.00 | 0.00 |
|  | *Psilocybe* |  | 28.37 | 1.20 | 0.78 | 1.02 | 1.08 | 0.00 | 0.39 | 0.25 | 10.37 | 1.77 |  | 0.00 | 0.00 | 0.00 | 0.00 | 0.41 | 0.00 |
|  |  | *coprophila* | 28.37 | 1.20 | 0.78 | 1.02 | 1.08 | 0.00 | 0.39 | 0.25 | 10.37 | 1.77 |  | 0.00 | 0.00 | 0.00 | 0.00 | 0.41 | 0.00 |
| *Diaporthales* | *unidentified* |  | 0.04 | 0.05 | 7.6 | 0.22 | 3.03 | 4.32 | 1.66 | 1.35 | 0 | 1.47 |  | 10.74 | 4.05 | 11.23 | 17.13 | 10.53 | 14.43 |
| *Malasseziales* | *Malassezia* |  | 0.13 | 0.95 | 45.64 | 3.21 | 19.25 | 54.26 | 17.74 | 8.95 | 1.10 | 15.25 |  | 83.45 | 92.67 | 79.78 | 78.66 | 83.22 | 80.63 |
|  |  | *globosa* | 0.06 | 0.11 | 6.11 | 0.00 | 9.37 | 14.58 | 7.28 | 2.21 | 0.14 | 4.15 |  | 31.96 | 34.29 | 32.20 | 33.68 | 26.33 | 29.84 |
|  |  | *restricta* | 0.06 | 0.84 | 39.53 | 3.21 | 9.88 | 39.68 | 10.46 | 6.75 | 0.95 | 11.10 |  | 51.49 | 58.39 | 47.58 | 44.98 | 56.89 | 50.79 |
| *Sordariales* | *Botryotrichum* |  | 0.00 | 0.00 | 0.00 | 0.00 | 0.00 | 1.12 | 0.00 | 0.61 | 0.00 | 5.28 |  | 0.00 | 0.00 | 0.00 | 0.00 | 0.00 | 0.00 |
|  |  | *atrogriseum* | 0.00 | 0.00 | 0.00 | 0.00 | 0.00 | 1.12 | 0.00 | 0.61 | 0.00 | 5.28 |  | 0.00 | 0.00 | 0.00 | 0.00 | 0.00 | 0.00 |
|  | *Chaetomium* |  | 0.00 | 0.00 | 0.00 | 0.00 | 0.00 | 0.00 | 0.00 | 0.00 | 0.00 | 0.00 |  | 0.00 | 0.00 | 0.00 | 0.00 | 0.00 | 0.00 |
|  | *Humicola* |  | 0.00 | 0.00 | 0.00 | 0.00 | 0.00 | 0.15 | 0.00 | 0.16 | 0.00 | 0.00 |  | 0.00 | 0.00 | 0.00 | 0.00 | 0.00 | 0.00 |
|  |  | *olivacea* | 0.00 | 0.00 | 0.00 | 0.00 | 0.00 | 0.15 | 0.00 | 0.16 | 0.00 | 0.00 |  | 0.00 | 0.00 | 0.00 | 0.00 | 0.00 | 0.00 |
|  | *Neurospora* |  | 0.00 | 0.00 | 1.23 | 1.24 | 0.00 | 0.10 | 0.42 | 0.00 | 0.00 | 1.81 |  | 0.00 | 0.00 | 0.00 | 0.00 | 0.90 | 0.00 |
|  |  | *terricola* | 0.00 | 0.00 | 1.23 | 1.24 | 0.00 | 0.10 | 0.42 | 0.00 | 0.00 | 1.81 |  | 0.00 | 0.00 | 0.00 | 0.00 | 0.90 | 0.00 |
|  | *Remersonia* |  | 0.00 | 0.00 | 0.00 | 0.00 | 0.00 | 0.00 | 0.00 | 0.00 | 0.00 | 0.00 |  | 0.00 | 0.00 | 0.00 | 0.00 | 0.00 | 0.00 |
|  |  | *thermophila* | 0.00 | 0.00 | 0.00 | 0.00 | 0.00 | 0.00 | 0.00 | 0.00 | 0.00 | 0.00 |  | 0.00 | 0.00 | 0.00 | 0.00 | 0.00 | 0.00 |
|  | *Schizothecium* |  | 0.00 | 0.00 | 0.00 | 0.00 | 0.89 | 0.00 | 1.88 | 0.00 | 0.40 | 1.77 |  | 0.00 | 0.00 | 0.00 | 0.00 | 0.00 | 0.00 |
| *Venturiales* | *Ochroconis* |  | 0.00 | 0.00 | 0.00 | 0.00 | 0.54 | 0.18 | 0.00 | 0.40 | 0.07 | 0.00 |  | 0.00 | 0.00 | 0.00 | 0.00 | 0.00 | 0.00 |
|  |  | *ramosa* | 0.00 | 0.00 | 0.00 | 0.00 | 0.54 | 0.18 | 0.00 | 0.40 | 0.07 | 0.00 |  | 0.00 | 0.00 | 0.00 | 0.00 | 0.00 | 0.00 |
|  | *Scolecobasidium* |  | 0.00 | 0.00 | 0.00 | 0.00 | 0.32 | 0.00 | 0.00 | 0.32 | 0.68 | 0.00 |  | 0.00 | 0.00 | 0.00 | 0.00 | 0.00 | 0.00 |

**Supplementary table 5**: relative abundance of *Fungi* in L-HeR and H-HeR soils at the taxonomic ranks of order, genus and species.

| Kingdom | Order | H-HeR | L-HeR | W | P |
| --- | --- | --- | --- | --- | --- |
| *Bacteria* | *Actinomycetales* | 13.82 ± 4.37 | 10.49 ± 1.86 | 35 | 0.587 |
| *Bacteria* | *Desulfurococcales* | 0.15 ± 0.13 | 1.29 ± 0.53 | 16 | 0.112 |
| *Bacteria* | *Gaiellales* | 1.32 ± 0.33 | 2.57 ± 0.51 | 16 | 0.128 |
| *Bacteria* | *Lactobacillales* | 2.52 ± 1.00 | 0.11 ± 0.03 | 52 | 0.016 * |
| *Archaea* | *Methanosarcinales* | 0.36 ± 0.22 | 4.9 ± 1.72 | 9.5 | 0.025 * |
| *Bacteria* | *Pseudanabaenales* | 0.13 ± 0.06 | 1.12 ± 0.49 | 9 | 0.022 |
| *Bacteria* | *Rhizobiales* | 4.21 ± 1.28 | 10.3 ± 2.84 | 11 | 0.039 * |
| *Bacteria* | *Solibacterales* | 0.66 ± 0.21 | 2.07 ± 0.38 | 6 | 0.009 ** |
| *Bacteria* | *Solirubrobacterales* | 0.66 ± 0.31 | 1.33 ± 0.5 | 22 | 0.378 |
| *Fungi* | *Agaricales* | 0.2 ± 0.1 | 7.62 ± 3.23 | 5.50 | 0.007 ** |
| *Fungi* | *Diaporthales* | 11 ± 1.83 | 1.45 ± 0.57 | 59.00 | 0.001 ** |
| *Fungi* | *Malasseziales* | 80.4 ± 3.15 | 12.3 ± 4.6 | 60.00 | 0.001 ** |
| *Fungi* | *Sordariales* | 0.14 ± 0.14 | 2.7 ± 1.03 | 5.50 | 0.006 ** |
| *Fungi* | *Venturiales* | 0 ± 0 | 0.19 ± 0.09 | 18.00 | 0.08 |

**Supplementary table 6**: Microbial orders with different abundance (mean values ± standard error values) in H-HeR and L-HeR paddies.

Mann-Whitney test (two tailed). W: value of Mann-Whitney statistics *: P < 0.05; **: P < 0.01.

| Physical-chemical properties | FR.1 | FR.2 | FR.3 | FR.4 | FR.5 | FR.6 | FR.7 | FR.8 | FR.9 | FR.10 | FR.11 | FR.12 | FR.13 | FR.14 | FR.15 | FR.16 | FR.17 | FR.18 | FR.19 | FR.20 | FR.21 | FR.22 | FR.23 | FR.24 | FR.25 | FR.26 | FR.27 | FR.28 | FR.29 | FR.30 | FR.31 | FR.32 |
| --- | --- | --- | --- | --- | --- | --- | --- | --- | --- | --- | --- | --- | --- | --- | --- | --- | --- | --- | --- | --- | --- | --- | --- | --- | --- | --- | --- | --- | --- | --- | --- | --- |
| coarse_sand | 4.00 | 0.00 | 0.00 | 0.00 | 10.00 | 6.00 | 8.00 | 158.00 | 0.00 | 36.00 | 88.00 | 128.00 | 6.00 | 30.00 | 4.00 | 78.00 | 0.00 | 10.00 | 6.00 | 4.00 | 4.00 | 176.00 | 60.00 | 0.00 | 0.00 | 0.00 | 44.00 | 12.00 | 14.00 | 4.00 | 4.00 | 4.00 |
| fine_sand | 300.00 | 830.00 | 371.00 | 620.00 | 720.00 | 361.00 | 120.00 | 451.00 | 420.00 | 380.00 | 631.00 | 711.00 | 411.00 | 500.00 | 650.00 | 471.00 | 220.00 | 281.00 | 471.00 | 491.00 | 531.00 | 551.00 | 531.00 | 591.00 | 411.00 | 560.00 | 470.00 | 580.00 | 520.00 | 590.00 | 410.00 | 660.00 |
| silt | 588.00 | 108.00 | 535.00 | 288.00 | 198.00 | 545.00 | 698.00 | 425.00 | 438.00 | 518.00 | 315.00 | 235.00 | 535.00 | 408.00 | 268.00 | 445.00 | 588.00 | 595.00 | 425.00 | 405.00 | 395.00 | 375.00 | 375.00 | 345.00 | 465.00 | 358.00 | 438.00 | 328.00 | 378.00 | 348.00 | 508.00 | 258.00 |
| clay | 112.00 | 62.00 | 94.00 | 92.00 | 82.00 | 94.00 | 182.00 | 124.00 | 142.00 | 102.00 | 54.00 | 54.00 | 54.00 | 92.00 | 82.00 | 84.00 | 192.00 | 124.00 | 104.00 | 104.00 | 74.00 | 74.00 | 94.00 | 64.00 | 124.00 | 82.00 | 92.00 | 92.00 | 102.00 | 62.00 | 82.00 | 82.00 |
| pH_H2O | 8.20 | 6.20 | 5.90 | 5.60 | 6.20 | 5.40 | 7.10 | 6.00 | 6.40 | 6.60 | 6.60 | 6.10 | 6.70 | 7.40 | 6.50 | 5.10 | 6.70 | 7.00 | 5.70 | 6.20 | 5.60 | 6.00 | 7.10 | 5.70 | 5.50 | 5.90 | 6.10 | 6.40 | 5.80 | 5.70 | 6.10 | 6.10 |
| pH_CaCl2 | 7.30 | 5.70 | 5.50 | 4.90 | 5.60 | 5.00 | 6.70 | 5.50 | 5.90 | 5.80 | 6.00 | 5.60 | 6.10 | 6.70 | 5.70 | 5.70 | 6.10 | 6.60 | 5.30 | 5.60 | 5.20 | 5.40 | 6.70 | 5.30 | 5.00 | 5.10 | 5.40 | 5.70 | 5.40 | 5.10 | 5.50 | 5.80 |
| S_org | 18.00 | 23.00 | 26.00 | 11.00 | 25.00 | 14.00 | 30.00 | 14.00 | 19.00 | 27.00 | 31.00 | 26.00 | 27.00 | 53.00 | 20.00 | 18.00 | 23.00 | 28.00 | 18.00 | 17.00 | 13.00 | 33.00 | 21.00 | 25.00 | 26.00 | 15.00 | 29.00 | 16.00 | 16.00 | 17.00 | 14.00 | 24.00 |
| C_org | 10.54 | 13.20 | 15.20 | 6.40 | 14.30 | 8.30 | 17.40 | 8.20 | 10.90 | 15.50 | 17.90 | 15.30 | 15.60 | 25.24 | 11.70 | 10.40 | 13.10 | 16.40 | 10.60 | 9.90 | 7.80 | 18.90 | 12.00 | 14.60 | 15.20 | 8.80 | 17.10 | 9.50 | 9.20 | 9.90 | 8.10 | 13.90 |
| C/N | 11.70 | 13.20 | 9.50 | 10.70 | 13.00 | 8.30 | 10.90 | 8.20 | 12.10 | 11.10 | 9.40 | 9.00 | 9.20 | 11.00 | 13.00 | 8.70 | 14.60 | 9.60 | 8.80 | 8.30 | 7.10 | 9.00 | 8.60 | 9.10 | 8.90 | 12.60 | 12.20 | 11.90 | 11.50 | 12.40 | 13.50 | 12.60 |
| CaCO3 | 43.00 | 0.00 | 0.00 | 0.00 | 0.00 | 0.00 | 0.00 | 0.00 | 0.00 | 0.00 | 0.00 | 0.00 | 0.00 | 48.00 | 0.00 | 0.00 | 0.00 | 0.00 | 0.00 | 0.00 | 0.00 | 0.00 | 0.00 | 0.00 | 0.00 | 0.00 | 0.00 | 0.00 | 0.00 | 0.00 | 0.00 | 0.00 |
| CaCO3_active | 6.00 | 0.00 | 0.00 | 0.00 | 0.00 | 0.00 | 0.00 | 0.00 | 0.00 | 0.00 | 0.00 | 0.00 | 0.00 | 5.00 | 0.00 | 0.00 | 0.00 | 0.00 | 0.00 | 0.00 | 0.00 | 0.00 | 0.00 | 0.00 | 0.00 | 0.00 | 0.00 | 0.00 | 0.00 | 0.00 | 0.00 | 0.00 |
| N_tot | 0.90 | 1.00 | 1.60 | 0.60 | 1.10 | 1.00 | 1.60 | 1.00 | 0.90 | 1.40 | 1.90 | 1.70 | 1.70 | 2.30 | 0.90 | 1.20 | 0.90 | 1.70 | 1.20 | 1.20 | 1.10 | 2.10 | 1.40 | 1.60 | 1.70 | 0.70 | 1.40 | 0.80 | 0.80 | 0.80 | 0.60 | 1.10 |
| CexC | 11.00 | 9.10 | 12.90 | 8.90 | 9.80 | 9.90 | 20.50 | 11.30 | 12.70 | 14.40 | 11.80 | 10.30 | 11.40 | 18.80 | 8.00 | 10.60 | 16.70 | 15.00 | 10.90 | 10.90 | 7.20 | 13.40 | 11.80 | 11.40 | 14.50 | 8.50 | 12.20 | 9.60 | 9.80 | 8.50 | 8.80 | 11.20 |
| Ca | 7.01 | 2.24 | 4.31 | 4.84 | 2.21 | 2.92 | 23.98 | 4.94 | 4.89 | 8.06 | 5.09 | 3.51 | 6.34 | 8.56 | 2.80 | 4.23 | 8.75 | 8.66 | 2.94 | 3.42 | 1.69 | 3.54 | 7.73 | 3.38 | 4.39 | 1.77 | 4.28 | 3.95 | 3.02 | 2.32 | 2.84 | 3.41 |
| Mg | 0.89 | 0.36 | 0.93 | 1.03 | 0.44 | 0.72 | 2.27 | 1.00 | 1.02 | 1.46 | 0.83 | 0.49 | 0.83 | 2.28 | 0.68 | 0.57 | 2.00 | 1.66 | 0.72 | 0.94 | 0.20 | 0.91 | 1.07 | 0.70 | 1.08 | 0.31 | 0.67 | 0.71 | 0.54 | 0.51 | 0.82 | 0.71 |
| K | 0.22 | 0.03 | 0.37 | 0.19 | 0.10 | 0.21 | 0.20 | 0.14 | 0.19 | 0.16 | 0.21 | 0.06 | 0.13 | 1.42 | 0.12 | 0.19 | 0.22 | 0.16 | 0.10 | 0.14 | 0.08 | 0.15 | 0.25 | 0.21 | 0.25 | 0.04 | 0.11 | 0.34 | 0.23 | 0.11 | 0.12 | 0.27 |
| Na | 0.04 | 0.02 | 0.10 | 0.26 | 0.03 | 0.05 | 0.10 | 0.07 | 0.25 | 0.10 | 0.10 | 0.06 | 0.30 | 0.05 | 0.03 | 0.05 | 0.10 | 0.09 | 0.06 | 0.03 | 0.02 | 0.08 | 0.13 | 0.06 | 0.10 | 0.04 | 0.08 | 0.03 | 0.07 | 0.04 | 0.04 | 0.08 |
| BS | 74.18 | 29.12 | 44.26 | 71.01 | 28.37 | 39.39 | 129.51 | 54.42 | 50.00 | 67.92 | 52.80 | 40.00 | 66.67 | 65.48 | 45.38 | 47.55 | 66.29 | 70.46 | 35.05 | 41.56 | 27.64 | 34.93 | 77.79 | 38.16 | 40.14 | 25.41 | 42.13 | 52.40 | 39.39 | 35.06 | 43.41 | 39.91 |
| Ca/Mg | 7.90 | 6.20 | 4.60 | 4.70 | 5.00 | 4.10 | 10.60 | 4.90 | 4.80 | 5.50 | 6.10 | 7.20 | 7.60 | 3.80 | 4.10 | 7.40 | 4.40 | 5.20 | 4.10 | 3.60 | 8.50 | 3.90 | 7.20 | 4.80 | 4.10 | 5.70 | 6.40 | 5.60 | 5.60 | 4.50 | 3.50 | 4.80 |
| Mg/K | 4.00 | 12.00 | 2.50 | 5.40 | 4.40 | 3.40 | 11.40 | 7.10 | 5.40 | 9.10 | 4.00 | 8.20 | 6.40 | 1.60 | 5.70 | 3.00 | 9.10 | 10.40 | 7.20 | 6.70 | 2.50 | 6.10 | 4.30 | 3.30 | 4.30 | 7.80 | 6.10 | 2.10 | 2.30 | 4.60 | 6.80 | 2.60 |
| ESP | 0.36 | 0.22 | 0.78 | 2.92 | 0.31 | 0.51 | 0.49 | 0.62 | 1.97 | 0.69 | 0.85 | 0.58 | 2.63 | 0.27 | 0.38 | 0.47 | 0.60 | 0.60 | 0.55 | 0.28 | 0.28 | 0.60 | 1.10 | 0.53 | 0.69 | 0.47 | 0.66 | 0.31 | 0.71 | 0.47 | 0.45 | 0.71 |
| P_ass | 60.00 | 31.00 | 65.00 | 78.00 | 10.00 | 62.00 | 8.00 | 24.00 | 20.00 | 29.00 | 46.00 | 9.00 | 56.00 | 107.00 | 8.00 | 38.00 | 9.00 | 30.00 | 52.00 | 43.00 | 47.00 | 36.00 | 100.00 | 53.00 | 30.00 | 15.00 | 35.00 | 45.00 | 32.00 | 28.00 | 33.00 | 71.00 |

**Supplementary table 7**: physical-chemical characters of the paddy soils.

S_org: organic matter. C_org: organic carbon. C/N: carbon/ nitrogen ratio. N_tot: total Nitrogen. CexC: Cation exchange Capacity. BS: Base Saturation. Mg/K: Magnesium/Potassium ratio. ESP: Exchangeable Sodium Percentage. P_ass: assimilable phosphorus.

| physical-chemical properties | Df | Variance | F | P |
| --- | --- | --- | --- | --- |
| coarse_sand | 1 | 55.777 | 0.779 | 0.481 |
| fine_sand | 1 | 56.842 | 0.794 | 0.481 |
| silt | 1 | 110.316 | 1.541 | 0.196 |
| pH_H2O | 1 | 171.329 | 2.393 | 0.071 |
| pH_CaCl2 | 1 | 107.748 | 1.505 | 0.202 |
| S_org | 1 | 19.097 | 0.267 | 0.920 |
| C_org | 1 | 59.842 | 0.836 | 0.430 |
| C_N_ratio | 1 | 32.007 | 0.447 | 0.752 |
| CaCO3 | 1 | 18.122 | 0.253 | 0.925 |
| CaCO3_active | 1 | 26.444 | 0.369 | 0.861 |
| N_tot | 1 | 196.847 | 2.750 | 0.058 |
| CexC | 1 | 54.890 | 0.767 | 0.466 |
| Ca | 1 | 25.380 | 0.355 | 0.849 |
| Mg | 1 | 52.999 | 0.740 | 0.500 |
| K | 1 | 54.161 | 0.757 | 0.502 |
| Na | 1 | 28.619 | 0.400 | 0.798 |
| BS | 1 | 119.968 | 1.676 | 0.171 |
| Ca_Mg_ratio | 1 | 27.532 | 0.385 | 0.829 |
| Mg_K_ratio | 1 | 40.427 | 0.565 | 0.644 |
| ESP | 1 | 29.143 | 0.407 | 0.812 |
| P_ass | 1 | 27.509 | 0.384 | 0.832 |
| Residual | 10 | 715.912 |  |  |

**Supplementary table 8**: results of the constrained correspondence analysis between bacterial, archaeal and fungal composition and soil physical-chemical properties. Df: degrees of freedom. F: value of constrained correspondence analysis statistics. **: P < 0.01.

| physical-chemical properties | NMDS1 | NMDS2 | R2 | P |
| --- | --- | --- | --- | --- |
| coarse_sand | -0.406 | 0.914 | 0.013 | 0.812 |
| fine_sand | 0.993 | -0.116 | 0.514 | 0.001 *** |
| silt | -1.000 | -0.013 | 0.494 | 0.001 *** |
| clay | -0.787 | 0.617 | 0.394 | 0.004 ** |
| pH_H2O | -0.816 | -0.578 | 0.423 | 0.003 ** |
| pH_CaCl2 | -0.845 | -0.535 | 0.513 | 0.002 ** |
| S_org | -0.959 | -0.282 | 0.354 | 0.004 ** |
| C_org | -0.995 | -0.102 | 0.319 | 0.004 ** |
| C/N | 0.776 | 0.631 | 0.023 | 0.724 |
| CaCO3 | -0.437 | -0.899 | 0.566 | 0.001 *** |
| CaCO3_active | -0.413 | -0.911 | 0.530 | 0.004 ** |
| N_tot | -0.994 | -0.113 | 0.290 | 0.004 ** |
| CexC | -0.964 | 0.266 | 0.789 | 0.001 *** |
| Ca | -0.956 | 0.293 | 0.646 | 0.001 *** |
| Mg | -0.980 | 0.201 | 0.803 | 0.001*** |
| K | -0.702 | -0.712 | 0.489 | 0.001*** |
| Na | -0.472 | 0.882 | 0.401 | 0.003 ** |
| BS | -0.971 | 0.239 | 0.672 | 0.001 *** |
| Ca/Mg | -0.922 | -0.387 | 0.034 | 0.586 |
| Mg/K | -0.049 | 0.999 | 0.207 | 0.037 * |
| ESP | -0.282 | 0.959 | 0.262 | 0.018 * |
| P_ass | -0.520 | -0.854 | 0.233 | 0.028 * |

**Supplementary table 9**: results of the envfit analysis of soil physical-chemical properties. Df: degrees of freedom. **: P < 0.01.

| physical-chemical properties | Df | Variance | F | P |
| --- | --- | --- | --- | --- |
| fine_sand | 1 | 69.457 | 2.376 | 0.120 |
| silt | 1 | 53.368 | 1.826 | 0.197 |
| pH_H2O | 1 | 35.134 | 1.202 | 0.297 |
| pH_CaCl2 | 1 | 3.653 | 0.125 | 0.855 |
| S_org | 1 | 8.097 | 0.277 | 0.759 |
| C_org | 1 | 3.914 | 0.134 | 0.879 |
| CaCO3 | 1 | 5.752 | 0.197 | 0.769 |
| CaCO3_active | 1 | 9.668 | 0.331 | 0.695 |
| N_tot | 1 | 44.421 | 1.520 | 0.274 |
| CexC | 1 | 21.514 | 0.736 | 0.477 |
| Ca | 1 | 86.911 | 2.973 | 0.072 |
| Mg | 1 | 1.977 | 0.068 | 0.942 |
| K | 1 | 8.882 | 0.304 | 0.734 |
| Na | 1 | 13.590 | 0.465 | 0.619 |
| BS | 1 | 21.035 | 0.720 | 0.499 |
| Mg/K | 1 | 4.196 | 0.144 | 0.865 |
| ESP | 1 | 8.389 | 0.287 | 0.764 |
| P_ass | 1 | 74.011 | 2.532 | 0.113 |
| Residual | 13 | 379.980 |  |  |

**Supplementary table 10**: results of the constrained correspondence analysis between HeR and soil physical-chemical properties. Df: degrees of freedom. F: value of constrained correspondence analysis statistics. **: P < 0.01.


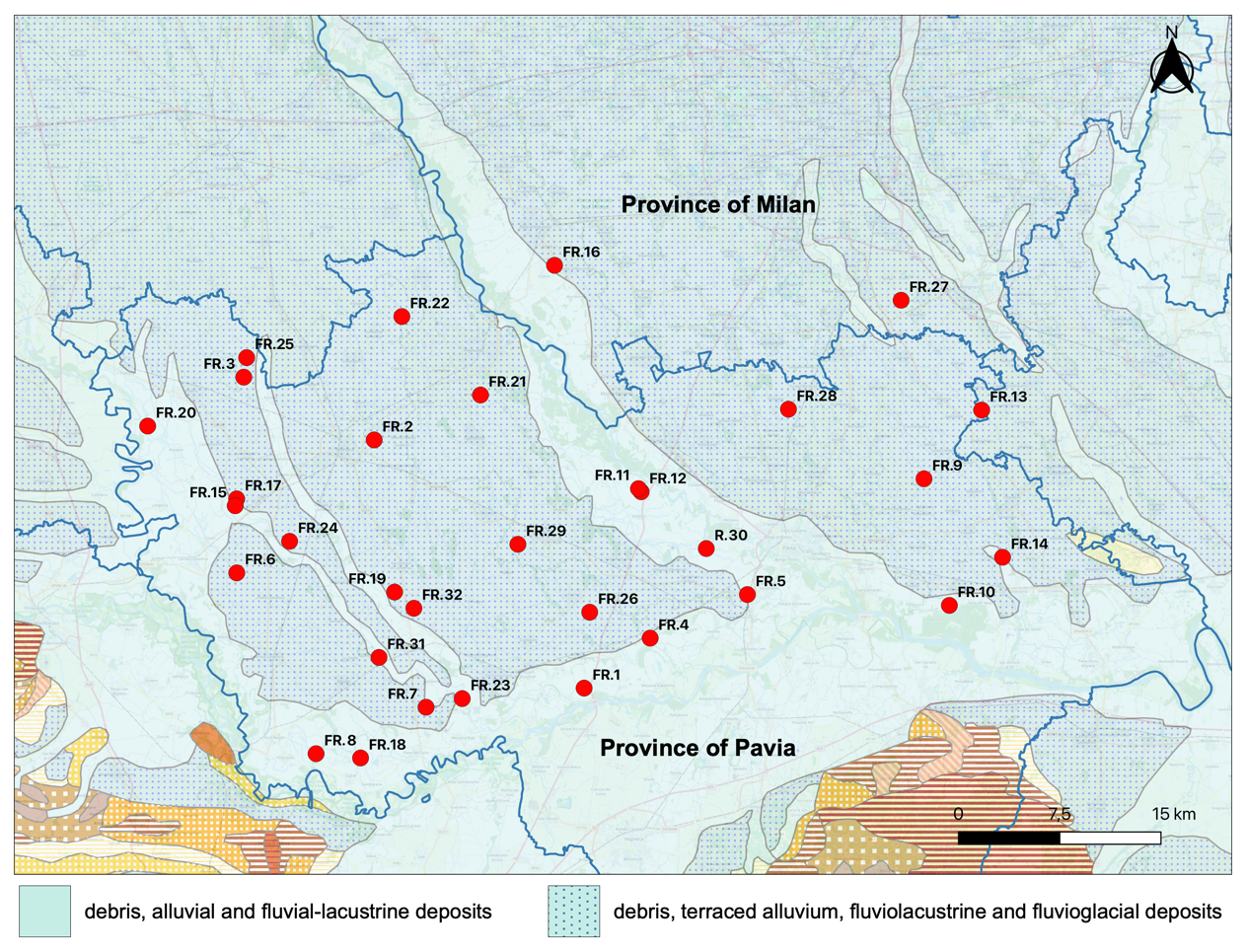


**Supplementary figure 1:** pedological map of the provinces of Milan e and Pavia. Geoportale Nazionale (<http://www.pcn.minambiente.it/mattm/>)
